# Preoperative transcranial direct current stimulation: Exploration of a novel strategy to enhance neuroplasticity before surgery to control postoperative pain. A randomized sham-controlled study

**DOI:** 10.1371/journal.pone.0187013

**Published:** 2017-11-30

**Authors:** Hugo Ribeiro, Ricardo Bertol Sesterhenn, Andressa de Souza, Ana Claudia de Souza, Monique Alves, Jessica Catarina Machado, Nathalia Bofill Burger, Iraci Lucena da Silva Torres, Luciana Cadore Stefani, Felipe Fregni, Wolnei Caumo

**Affiliations:** 1 Department of Clinical Research Center, Laboratory of Pain & Neuromodulation, Hospital de Clínicas de Porto Alegre, Universidade Federal do Rio Grande do Sul, Porto Alegre, Rio Grande do Sul, Brazil; 2 Surgery Department, Hospital Independência, Porto Alegre, Rio Grande do Sul, Brazil; 3 Postgraduate Program in Health and Human Development, La Salle University Center, Canoas, Rio Grande do Sul, Brazil; 4 Pharmacology Department, Instituto de Ciências Básicas da Saúde, Universidade Federal do Rio Grande do Sul, Porto Alegre, Rio Grande do Sul, Brazil; 5 Surgery Department, Anesthesia and Perioperative Medicine, Hospital de Clínicas de Porto Alegre, Universidade Federal do Rio Grande do Sul, Porto Alegre, Rio Grande do Sul, Brazil; 6 Physical Medicine & Rehabilitation Department, Center of Neuromodulation & Center for Clinical Research Learning, Spaulding Rehabilitation Hospital, Harvard Medical School, Boston, Massachusetts, United States of America; 7 Pain and Palliative Care Service, Laboratory of Pain & Neuromodulation, Hospital de Clínicas de Porto Alegre, Universidade Federal do Rio Grande do Sul, Porto Alegre, Rio Grande do Sul, Brazil; University of Bologna, ITALY

## Abstract

**Background:**

An imbalance in the excitatory/inhibitory systems in the pain networks may explain the persistent chronic pain after hallux valgus surgery. Thus, to contra-regulate this dysfunction, the use of transcranial direct current stimulation (tDCS) becomes attractive.

**Objective:**

We tested the hypothesis that two preoperative active(a)-tDCS sessions compared with sham(s)-tDCS could improve the postoperative pain [as indexed by Visual Analogue Scale (VAS) at rest and during walking (primary outcomes)]. To assess their effect on the change in the Numerical Pain Scale (NPS0-10) during Conditioned Pain Modulation (CPM-task), disability related to pain (DRP) and analgesic consumption (secondary outcomes). Also, we assessed if the brain derived neurotrophic factor (BDNF) in the cerebral spinal fluid (CSF) after tDCS could predict the intervention’s effect on the DRP.

**Methods:**

It is a prospective, double blind, sham-controlled, randomized single center, 40 women (18–70 years-old) who had undergone hallux valgus surgery were randomized to receive two sessions (20 minutes each) of anodal a-tDCS or s-tDCS on the primary motor cortex at night and in the morning before the surgery. To assess the DRP was used the Brazilian Profile of Chronic Pain: Screen (B-PCP:S).

**Results:**

A-tDCS group showed lower scores on VAS at rest and during walking (P<0.001). At rest, the difference between groups was 2.13cm (95%CI = 1.59 to 2.68) while during walking was 1.67cm (95%CI = 1.05 to 2.28). A-tDCS, when compared to s-tDCS reduced analgesic doses in 73.25% (P<0.001), produced a greater reduction in B-PCP:S (mean difference of 9.41 points, 95%CI = 0.63 to 18.21) and higher function of descending pain modulatory system (DPMS) during CPM-task.

**Conclusion:**

A-tDCS improves postoperative pain, the DRP and the function of DPMS. Also, the CSF BDNF after a-tDCS predicted the improvement in the DRP. In overall, these findings suggest that a-tDCS effects may be mediated by top-down regulatory mechanisms associated with the inhibitory cortical control.

**Trial registration:**

ClinicalTrials.gov NCT02360462

## Introduction

Foot pain affects 17 to 42% of the adult population [1] while hallux valgus is responsible for 28% of all causes [1,2]. Surgery is the only treatment to improve disability related to pain for patient refractory to conservative treatment [3]. However, one year after hallux valgus surgery, moderate-to-severe chronic pain persists in 21% at rest and 43% during walking [4]. This persistent pain after corrective surgery irrespective of visible injury is due to an abnormal responsiveness state to nociceptive stimuli, with a disproportionate pain perception to noxious stimulation magnitude. The imbalance between excitatory and inhibitory system explain the hyperexcitability in the pain networks, which has been postulated as a central process in chronic pain, and also they are part of the mechanisms that constitute central sensitization syndrome [5].

Central sensitization syndrome can explain the negative impact on quality of life associated with hallux valgus chronic pain [6]. There is a need therefore for the development approaches to improve the imbalance between the excitatory and inhibitory in pain pathways. Thus, the improvement in the disinhibited state may prevent the persistent pain after surgery [7]. Among potential alternatives, emerge the transcranial direct current stimulation (tDCS) as a targeted central technique to modulate cortical excitability in chronic pain. Robust and extensive basic science research over the past 40 years has demonstrated the effects of tDCS [8]. Conversely, recent human studies have confirmed that anodal stimulation increases cortical excitability while cathodal stimulation decreases it [9]. Importantly, consistent evidence has shown that chronic pain is associated with a disinhibited state of cortical neural circuits [5,10] that are reverted after anodal tDCS of primary motor cortex [11]. In addition, this neural disinhibited state may be related to the level of brain-derived neurotrophic factor (BDNF), which is a protein known to regulate the development of plasticity [12]. BDNF has important synaptic effects: increases spontaneous frequency of neuronal action potentials, potentiate inhibitory and excitatory circuits, interferes in neuromodulation of cholinergic, dopaminergic, noradrenergic and GABAergic inter-neurons [12]. Therefore, the relationship between BDNF levels and pain can confirm the influence of this biomarker in sustained pain conditions.

Cumulative evidence suggests that tDCS is a therapeutic tool that is relatively inexpensive, non-invasive, painless, safe, and used efficiently among double-blinded studies [13]. Also, it has revealed effectiveness in relieving chronic pain in a meta-analysis [14,15] as well as acute pain [16]. In the present study we hypothesized that active (a)-tDCS would improve the pain measurements (i.e. score in the Visual Analogue Pain Scale [VAS(0-10cm)], analgesic consumption and disability related to pain (DRP). Also, we hypothesized that a-tDCS effect could be related to brain-derived neurotrophic factor (BDNF) in the cerebral spinal fluid (CSF). In order to test our main hypothesis we compared the effect of a-tDCS with s-tDCS in the VAS at rest and during walking (primary outcome). To test the secondary hypothesis we compared their effect in the variation on Numerical Pain Scale [NPS(0–10)] scores during the conditioned pain modulation (CPM)-task, in the analgesic consumption and in the DRP. Also, we examined the relationship between the preoperative CSF BDNF level after two sessions of tDCS on the DRP at the follow up end. We hypothesize that the a-tDCS is better than s-tDCS to improve the postoperative pain and correlated measures and that its effect on pain could be related to BDNF.

## Material and method

### Design overview, setting, and participants

**Fig 1:**
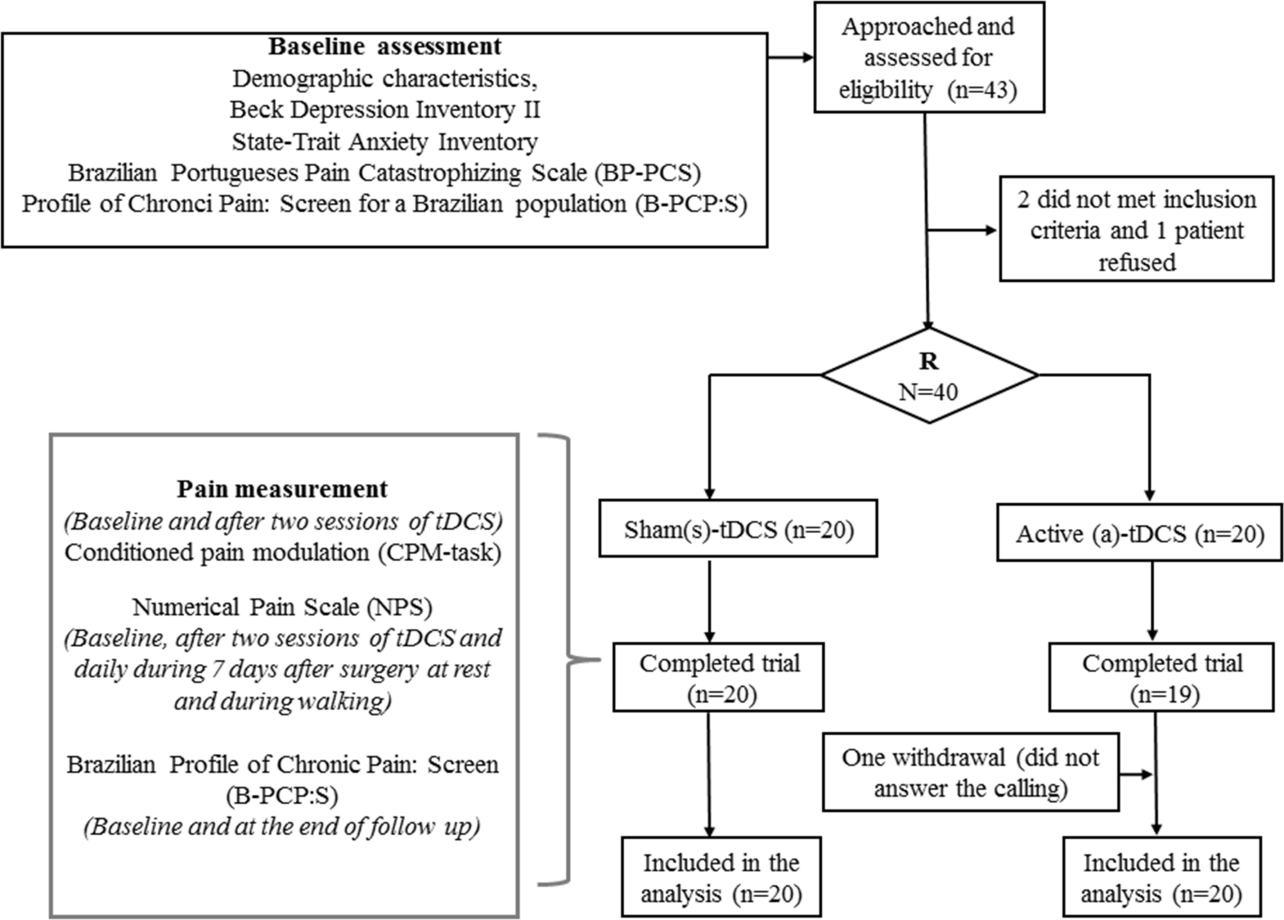
Randomization and follow-up of study subjects.

This double-blinded, in parallel, sham-controlled study was approved by the IRB of the Hospital de Clínicas de Porto Alegre (HCPA/UFRGS) (approval number: 14–0643). This study was registered in Clinical Trials: *https://clinicaltrials.gov*; the registration number: NCT02360462; the principal investigator's name: Wolnei Caumo and the date of registration: January 22, 2015. All participants gave written informed consent. We included 40 right-handed women between 18 and 70 years old, who were candidates to hallux valgus surgical treatment due to chronic foot pain. All surgical procedures were performed in Hospital Independência and all laboratory tests were done in HCPA. We excluded patients who have received previous tDCS treatment, and those with regional anesthesia contraindications, language or communication difficulties, mental impairment, diabetes, history of congestive heart failure, valvular, oncological, renal or hepatic disease. Patients with tDCS contra-indications [9] were excluded. No changes in trial methods were made after trial commencement. The flow chart of the study is presented in Fig 1. Study protocol is available at https://dorecuidadospaliativos.com.br/wp-content/uploads/2017/07/ProtocolHalluxValgus.pdf.

### Intervention

**Fig 2:**
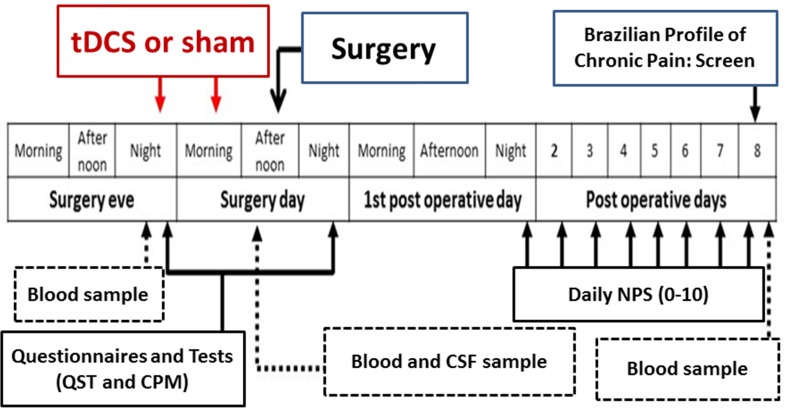
Experimental design—Assessments and interventions on each patient.

The anode was positioned over the left M1, and the cathode was placed over the right supraorbital region. Rubber electrodes were inserted into a 35cm2 sponge (moistened with NaCl). Current was two mA, and an elastic band maintained electrodes attached to the scalp. For sham conditions, the device was set to automatically turn off after 30 seconds of initial stimulation, which is a reliable method of mimicking real stimulation [17], keeping patients and investigators blinded. All patients underwent two sessions of active(a)-tDCS or sham(s)-tDCS applied during 20 minutes from 4:00 to 8:00 P.M., on the day before surgery and from 8:00 to 10:00 A.M. on the day of surgery. Experimental design and assessments are presented in Fig 2.

### Outcomes and assessments

The primary outcome was the pain score on the VAS(0–10)–scores ranged from no pain (zero) to worst possible pain (ten)–for the first 48 postoperative hours. Patients were asked daily to rate their surgical site pain during the last 24 hours in two conditions: the cumulative mean of the daily pain at rest and worst pain during walking. Secondary outcomes included daily analgesic drug consumption and score variation on the NPS(0–10) during CPM-task, as well as the score of the Brazilian Profile of Chronic Pain: Screen (B-PCP:S) [18] for quick identification of individual’s multidimensional pain experience (severity, interference in daily activity and emotional burden) [19]. DRP was based on the restriction related to chronic or recurrent pain with concurrent discomfort [20]. Analgesic use during patients’ hospital stay was registered and analgesic doses used after discharge was reviewed by a daily phone call. Total postoperative analgesic consumption was considered for analysis.

To measure the CPM-task we evaluated the pain intensity in two tonics heat pain threshold test stimuli followed by a CPM-task. The heat pain threshold was induced by the Quantitative Sensory Testing (QST) using the method of limits with a computer Peltier-based device thermode (30x30mm) [21]. A thermode was attached to the dominant ventral mid-forearm skin with a temperature set at 32°C increasing 1°C/s until maximum 52°C. We used the heat pain threshold as conditioning pain stimulus to elicit a prolonged pain sensation to trigger CPM. CPM-task consisted of non-dominant hand immersion to cold water (0−1°C) for 1min. A thermostat maintained water temperature. The QST procedure started after 30s of cold-water immersion. To determine the CPM, we used the difference between the pain score on NPS(0–10) QST and during cold water immersion (QST+CPM) at the temperature point in which subjects felt 6/10 pain on NPS(0–10) (during the initial period).

### Other assessments

Blood samples were collected in three moments: baseline, moment of spinal anesthesia and last day of follow-up. 1mL of CSF was collected during spinal anesthesia. CSF and blood samples were centrifuged in plastic tubes, for 10min, at 4500rpm in 4°C and stored in -80°C. Serum and CSF BDNF were determined by Enzyme-Linked Immunosorbent Assay (ELISA) using a ChemiKine BDNF Sandwich ELISA Kit, CYT306 (Chemicon/Millipore, Billerica, MA, USA) (BDNF lowest detection limit = 7.8pg/mL).

Demographic data and medical comorbidities were registered using structured questionnaire. Psychological tests used were validated for the Brazilian population. Patients’ baseline depressive symptoms were assessed by the Beck Depression Inventory-II [22]. Anxiety was measured using the State-Trait Anxiety Inventory (STAI) refined version of Rash analysis [23]. Pain-related catastrophic thinking was evaluated using the Brazilian Portuguese Catastrophizing Scale (B-PCS) [24]. To assess safety, we used the Systematic Assessment for tDCS Treatment questionnaire based on previously reported adverse events [8].

### Sample size justification

The sample size was estimated considering, as primary outcome, the cumulative mean of the worst daily pain scores in the VAS during the first 48 postoperative hours, in order to detect a difference between the means of 1.5 cm (SD 0.9) divided into two balanced groups (n = 7) in 1:1 ratio to reject the null hypothesis for an error type I equal 5% and error type II of 20. This difference between means has been considered clinically relevant in the context of treatment of acute postoperative pain in different scenarios [25]. Although the sample size estimation was 14, we defined a sample size of 28 patients, allocated randomly in 2 groups of 14 patients. Predicting possible losses and multiple outcomes, the final sample size was increased for 40 patients (20 per group). There were no interim analysis.

### Randomization

A researcher (A.S.) who was not involved in recruitment generated a permuted block randomization with block size four in appropriate software (www.randomization.com). Before recruitment phase, opaque envelopes containing patient allocation group were sealed and numbered sequentially. Patients’ sequence followed the surgeon (R.B.) agenda and the anesthesiologist (H.R.) was responsible for patient enrollment. After each patient agreed to participate in the treat and baseline evaluation was performed, the sequential envelope was opened and destroyed after programming tDCS device, by a researcher (M.A., J.C.M., or N.B.B.), who was not involved in direct patient evaluation.

### Blinding

Patients, surgical staff, study investigators and researchers who followed-up patients (H.R., R.B.), who entered and analyzed data (H.R., W.C.) were masked to allocation. Only the researcher responsible for programing tDCS device (M.A., J.C.M., or N.B.B.) was unmasked to group allocation. Further, to assess whether blinding was adequate, participants were asked to guess if they received active or sham tDCS and to rate their answer’s confidence with a five category Likert scale (no confidence to completely confident) upon experiment completion. The complete randomized allocation sequence list was kept in secret by its generator (A.S.) until all data was collected and analyzed. After all data collection, group allocation data were separated into group 0 and 1 for statistical analyses and its identity (intervention and control group) was revealed only after all analyses were completed.

### Anesthesia, surgery, and analgesia

The night prior to surgery, the same anesthetist evaluated participants and provided information of their perioperative course and postoperative pain management. Upon surgical room arrival, patients underwent standard monitoring. All patients underwent spinal anesthesia at lumbar segments (L3/L4 or L4/L5) with a 25G Quincke spinal needle. Spinal anesthesia and sedation is the most common anesthesia technique for this type of surgery in the referred hospital. After CSF collection, hyperbaric bupivacaine 12,5mg and morphine 80μg was administered. Intravenous fentanyl 50μg, midazolam 1 to 5mg and continuous propofol (0.08–0.1mg.kg.min-1) were applied to maintain unconsciousness.

In the recovery room, supplementary analgesia was administered intravenously as follows: dipyrone 1000mg 6/6h if the VAS(0–10) score was lower than three (weak pain), tramadol 50mg 6/6h if the VAS(0–10) score was higher than three (moderate to intense pain). If the pain persisted, 3mg of intravenous morphine was administered every 3h until achieve pain relief. On the next day, 24h after surgery, patients were discharged with oral analgesic prescription: dipyrone 1000mg 6/6h if weak pain, and tramadol 50mg 6/6h if moderate to intense pain. Analgesic consumption was registered in pain diaries. For analytical purposes, the number of analgesic doses of the seven postoperative days was considered.

All anesthesia procedures were performed by the same anesthesiologist, and all surgeries were performed by the same surgeon using the same technique (Chevron + Akin osteotomy).

### Statistical analysis

Categorical variables were summarized using conventional descriptive statistics. T-test for independent sample, chi-square or Fisher’s exact tests were used to compare continuous and categorical variables, respectively, for the main: post-operative pain differences between two groups. For non-parametric distributions, group comparisons were performed using the Wilcoxon-Mann-Whitney test.

Treatment effects were estimated through a Mixed Model for Repeated Measurements (MMRM), assuming an unstructured correlation matrix [26]. Time of measurement was coded to contrast mean baseline scores to a mean scores of seven-days follow-up. Separate analysis were conducted on data from treatment (a-tDCS or s-tDCS) and each outcome measurement (pain score in the VAS at rest and during walking, and analgesic doses). The models included fixed effects for treatment group. We tested the interaction between treatment group and time, and subject identification. The main effect of the predicted marginal mean differences between interventions was calculated by pairwise comparisons with an adjustment to account for multiple comparisons by the Bonferroni’s Test. Missing values were not a significant problem in the analysis of the data set, because we lost only four daily assessments in one patient (5%). To analyze the correlation between the CSF BDNF and Δ-B-PCP:S score value (i.e., follow-up end minus baseline level), the Spearman’s correlation analysis was used. The effect size of Wilcoxon Signed-rank test was assessed by z value, such as the r proposed by Cohen’s guidelines in which a large effect is .5, a medium effect is .3, and a small effect is .1. Whereas within groups, the standardized mean difference (SMD) was computed in terms of the ratio between the mean change and the pool of baseline standard deviation (SD). The SMD was interpreted as follows: small, 0.20 to 0.4; moderate, 0.50 to 0.70 and large, 0.80 or higher. All analyses were adjusted for multiple comparisons using the Bonferroni Test. All analyses were performed with two-tailed tests at the 5% significance level. Data were analyzed using SPSS, version 22.0 (SPSS, Chicago, IL).

## Results

### Demographic and clinical characteristics

Recruitment and follow-up period were held between December 2014 and December 2015. Forty patients were randomized into two groups (s-tDCS or a-tDCS). Thirty-nine patients completed the study; there was only one dropout in the a-tDCS group on the fifth postoperative day because the subject stopped answering phone calls (lost to follow-up). For this dropout, there were missing observations on the NPS scores starting at the fifth postoperative day, the last B-PCP:S and missing values for the third blood sample (serum BDNF), which was not collected. We included all 40 patients though in the analysis using intention-to-treat. Demographic and clinical characteristics are presented in Table 1. Baseline features were balanced between groups (p values >0.05). Severe or moderate side effects to intervention was not observed. Twenty-six patients of the sample, 13 of each group (a-tDCS and s-tDCS) (65%) underwent left foot surgery.

**Table 1:** Baseline epidemiological and clinical characteristics according to treatment group. Values are given as mean (SD) or frequency (percentage) (n = 40).

	s-tDCS (n = 20)	a-tDCS (n = 20)
Age (years)^*€*^	46.00 (13.55)	48.36 (10.97)
Education (years)^*€*^	13.40 (3.48)	12.18 (3.60)
Smoking (yes)^*¥*^	2 (16.67%)	1 (8.33%)
Alcohol (yes)^*¥*^	10 (50%)	10 (50%)
Clinical Comorbidity (yes)^*¥*^	7 (58.33%)	9 (75%)
Hypertension	4 (33.33%)	2 (16.67%)
Hypothyroidism	1 (8.33%)	—-
Other	2 (16.67%)	3 (25%)
History of psychiatric disease (Yes)^*¥*^	6 (50%)	7 (58.33%)
Mean pain in Rest on Visual Analogue Scale (VAS 0–10)^*€*^	5.89 (3.20)	5.70 (3.49)
Beck Depression Inventory II^*€*^	13.43 (8.63)	16.82 (10.90)
State-Anxiety on STAI^*€*^	23.80 (18.35)	22.82 (7.47)
Trait-A anxiety on STAI^*€*^	26.80 (8.35)	27.73 (8.93)
Brazilian Portuguese Catastrophizing Scale (B-PCS)^*€*^	29.00 (15.43)	28.45 (11.86)
Change on NPS (0–10) during CPM-task at the baseline^π^	-0.65 (2.27)	-0.40 2.09)
Profile of Chronic Pain: Screen for Brazilian population (B-PCP:S)^*€*^	60.58 (14.96)	60.78 (11.39)
Pain intensity reported on B-PCP:S^*€*^	24.75 (3.05)	23.65 (3.80)
Interference with activities reported on B-PCP:S ^*€*^	19.08 (7.26)	21.70 (8.37)
Emotional burden due pain reported on B-PCP:S^*€*^	16.10 (7.61)	16.08 (5.97)
Serum BDNF before tDCS (n/gml)^π^	10.98 (5.79)	10.39 (5.45)
Serum BDNF after two sessions of tDCS before surgery (n/gml) ^π^	6.40 (4.60)	8.06 (6.13)
CSF BDNF after two sessions of tDCS before surgery (n/gml)^*€*^	11.30 (3.26)	12.18 (3.07)

Active(a)-tDCS and sham(s)-tDCS.

€ Compared using t-test for independent samples;

Π Compared using Wilcoxon-Mann Whitney;

¥ compared using Pearson Chi-Square or Fisher's Exact Test.

### Primary outcomes analysis

#### Effect of treatment during the first 48 hours after surgery: Cumulative worst daily pain scores in the VAS scores (D0-D1)

**Fig 3:**
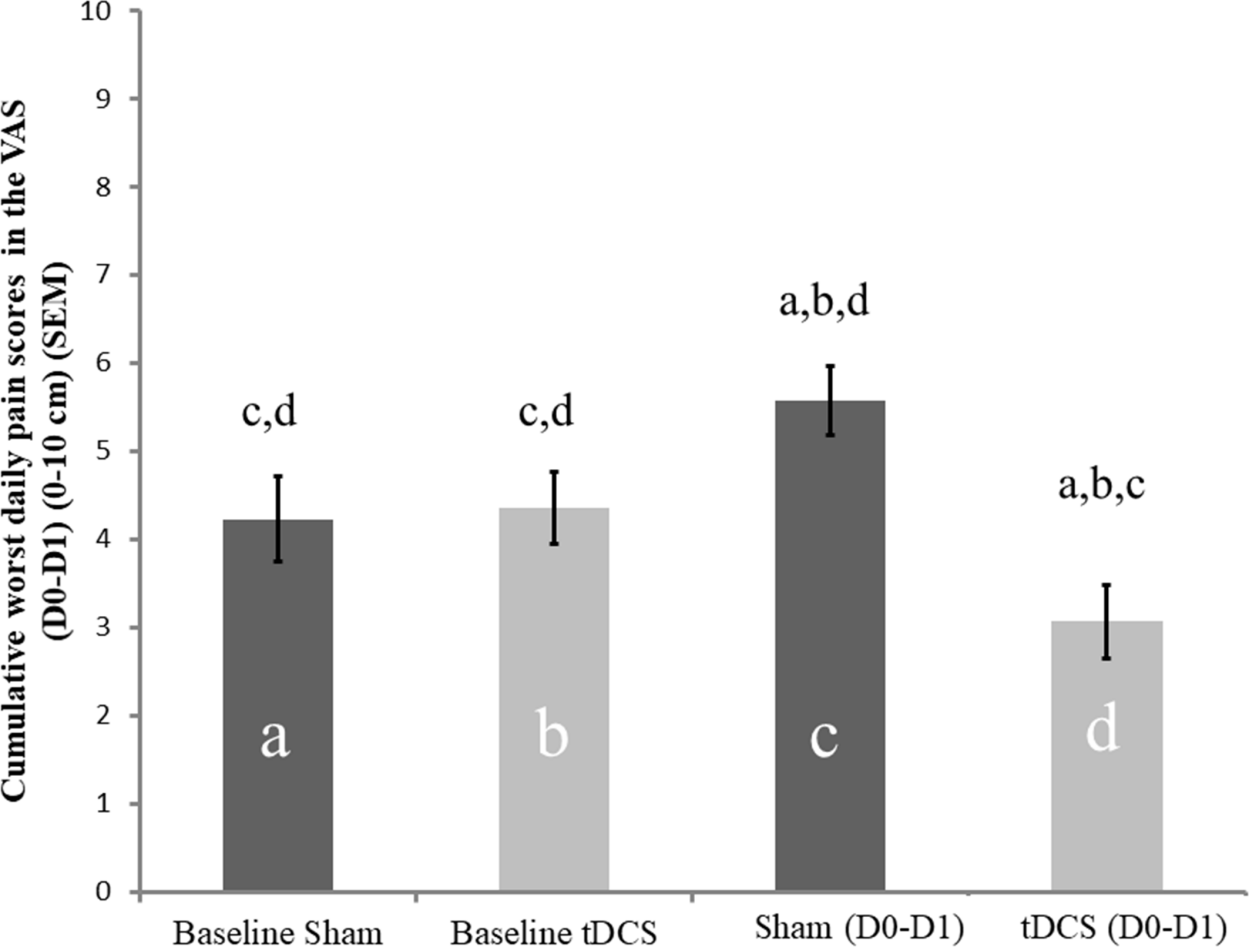
Comparisons of the pain scores in the Visual Analogue Scale (VAS) between s-tDCS and a-tDCS groups pre-treatment and in the postoperative cumulative pain scores (VAS D0-D1). Error bars indicate standard error of the mean (S.E.M.). Letters indicates a significant difference between the sham group and tDCS group (P < 0.05) assessed by t-test for independent sample.

To compare the average on VAS pain scores before and after treatment, the t-test was used for the independent sample. The average in the pain score pre-treatment in the s-tDCS and a-tDCS groups was 4.232 (1.78) vs. 4.461 (1.95), respectively. In the baseline, there was no statistically significant difference between interventions groups in the pain score [t = -0.255, df = 38.93; P = 0.823]. The cumulative mean (SD) of the worst daily pain scores during the first 48 hours after surgery in the s-tDCS group and in the a-tDCS group was 5.582 (2.17) vs. 3.070 (1.86), respectively. The cumulative average in the worst daily pain scores increased 31.89% from pre to postoperative in the s-tDCS whereas it decreased 31.18% in the a-tDCS group. The t-test for independent sample revealed a statistically significant difference between treatment groups [t = 3.55, df = 33.49; P = 0.001]. The average difference in the worst daily pain scores in the postoperative period between s-tDCS and a-tDCS groups was 2.361, and the SMD was 1.269 [2.361/1.865 (pooled of SD)]. The treatment effect assessed by the SMD produced a large size effect. The mean in the VAS pain score in the pre intervention and the cumulative mean of pain score during the first 48 postoperative hours is presented in Fig 3.

#### Effect of treatment throughout the postoperative period (D0-7): VAS scores at rest and during walking

**Fig 4:**
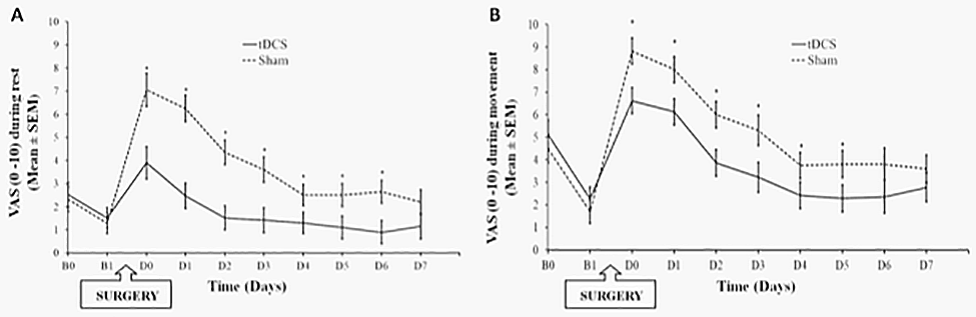
Visual Analogue Scale (VAS 0–10) scores at baseline and during follow-up. Fig A presents pain on VAS during rest and Fig B, pain on VAS during walking. Error bars indicate standard error of the mean (SEM). Asterisks (*) positioned above symbols indicate significant differences (p<0.01) at those time points using Bonferroni’s test. B0 = baseline. B1 = moment before surgery. D (0–7) = postoperative days.

A mixed model was used to compare the treatment effect across all seven days in the pain scores (at rest and during walking). The mixed model revealed that the a-tDCS group had significantly lower VAS scores for pain during rest [F = 44.14; P<0.001] throughout the postoperative period (Fig 4A). There is a significant effect of time [F = 9.45; P< 0.001], and an interaction between group and time [F = 3.26; P = 0.003]. The a-tDCS group’s marginal mean pain had a significant reduction of 2.15cm (95% CI, 1.59 to 2.68) compared to s-tDCS.

The mixed model also revealed that the a-tDCS group had significantly lower VAS scores for worst pain during walking [F = 19.34; P<0.001] throughout the postoperative period (Fig 4B). There is a significant effect of time [F = 20.29; P< 0.001], and there was no interaction between group and time [F = 1.59; P = 0.13]. The a-tDCS group’s marginal mean pain had a significant reduction in pain of 1.67cm (95% CI, 1.05 to 2.28) compared to s-tDCS.

### Secondary outcomes analysis: Analgesic consumption, change on NPS (0–10) during CPM-task and B-PCP:S score at follow-up

#### Analgesic consumption

The estimated marginal means of daily analgesic doses assessed using the mixed model was 1.37 (0.63) vs. 1.81 (0.64) for a-tDCS and s-tDCS, respectively. The a-tDCS group had a significant reduction in daily analgesic doses [F = 9.31; P<0.001) in all postoperative days (D0-D7). The a-tDCS group reduced analgesic doses by 73.25% (P<0.001) compared with the placebo-sham group. There is a significant effect of time [F = 43.13]; P<0.001, and there was no interaction between group and time [F = 1.25; P = 0.281].

#### Assessment of the treatment effect in the descending pain modulatory system as assessed by the change in the NPS (0–10) during the CPM-task, B-PCP:S score at follow-up end

The mean standard deviation (SD), the median interquartile (Q25-75) at baseline (A) and after intervention or at the follow-up end (B) are presented in Table 2. The intervention’s effect between groups (a-tDCS or s-tDCS) were compared by the Wilcoxon-Mann Whitney test. We observed that the a-tDCS improved the function of the descending pain modulatory system as assessed by the change on the NPS(0–10) during CPM-task. Also, it improved the DRP at the follow up end. The intervention’s effect in the CPM-task, estimated by the effect size of Wilcoxon Signed-rank test, showed a medium size effect [(z value (2.40)/ (6.32, value of N root square), the effect size equal to 0.38]. The intervention’s effect in the B-PCP:S score, estimated by the effect size of Wilcoxon Signed-rank test, showed a medium size effect [(z value (2.36)/ (6.32, value of N root square), the effect size equal to 0.37].

**Table 2:** Change on NPS(0–10) during CPM-task and B-PCP:S score. Data area presented as median and interquartile (Q)_25-75,_ mean (SD) and Δ-value (n = 40).

*Secondary outcomes*						
Groups	Median (Q)_25-75_	Mean (SD)	Δ-value	Z	P	P*
	B vs. A	B vs. A	(B minus A)			
**Change on NPS (0–10) during CPM-task**						
a-tDCS (n = 20)	-2.5(-5; 1) vs. 2 (-4; 2)	-2.63 (1.36) vs. -1.80 (2.04)	-0.8 (1.89)	-2.40	0.01	0.021
s- tDCS (n = 20)	-1.5 (-5.67; 3) vs. -2 (-4; 1)	-1.30 (2.38) vs.-1.95 (1.31)	0.65 (1.77)			
**Assessment of the disability related to pain by the B-PCP:S score at follow-up end**						
a-tDCS (n = 20)	16 (10.50; 28.50) vs. 57(46.50; 67.50)	17.71(13.0) vs. 60.78(11.39)	- 43.07 (17.72)	-2.36	0.01	0.024
s- tDCS (n = 20)	27 (20.5; 33.25) vs. 58 (48; 68)	27.13(14.33) vs. 60.58 (14.96)	- 33.35 (16.41)			

Wilcoxon-Mann Whitney test. Δ-B-PCP:S score (at baseline and the end of follow-up); Δ-CPM-task (at baseline and after intervention). The baseline value is indicated by (A) and after intervention or at the follow-up end (B).

*P-value adjusted by multiple comparisons by Bonferroni Test considering the three secondary outcomes (Δ-value CPM-task; Δ-value B-PCP:S score; analgesic consumption; after the adjustment all P values were <0.05).

#### Secondary analysis: Relationship between CSF BDNF and disability related to pain at follow-up end

The mean of CSF BDNF in the a-tDCS group after the second session was 12.65 (2.86) and the median and interquartile (Q25-75) was 11.15 (6.95; 18.26). Whereas, in the s-tDCS, the mean (SD) after the second session was 11.30 (3.27) and the median and interquartile (Q25-75) was 12.57 (6.16; 16.95). The difference across groups in the CSF BDNF was compared using the Wilcoxon-Mann Whitney test (z = -1.42; P = 0.152).

**Fig 5:**
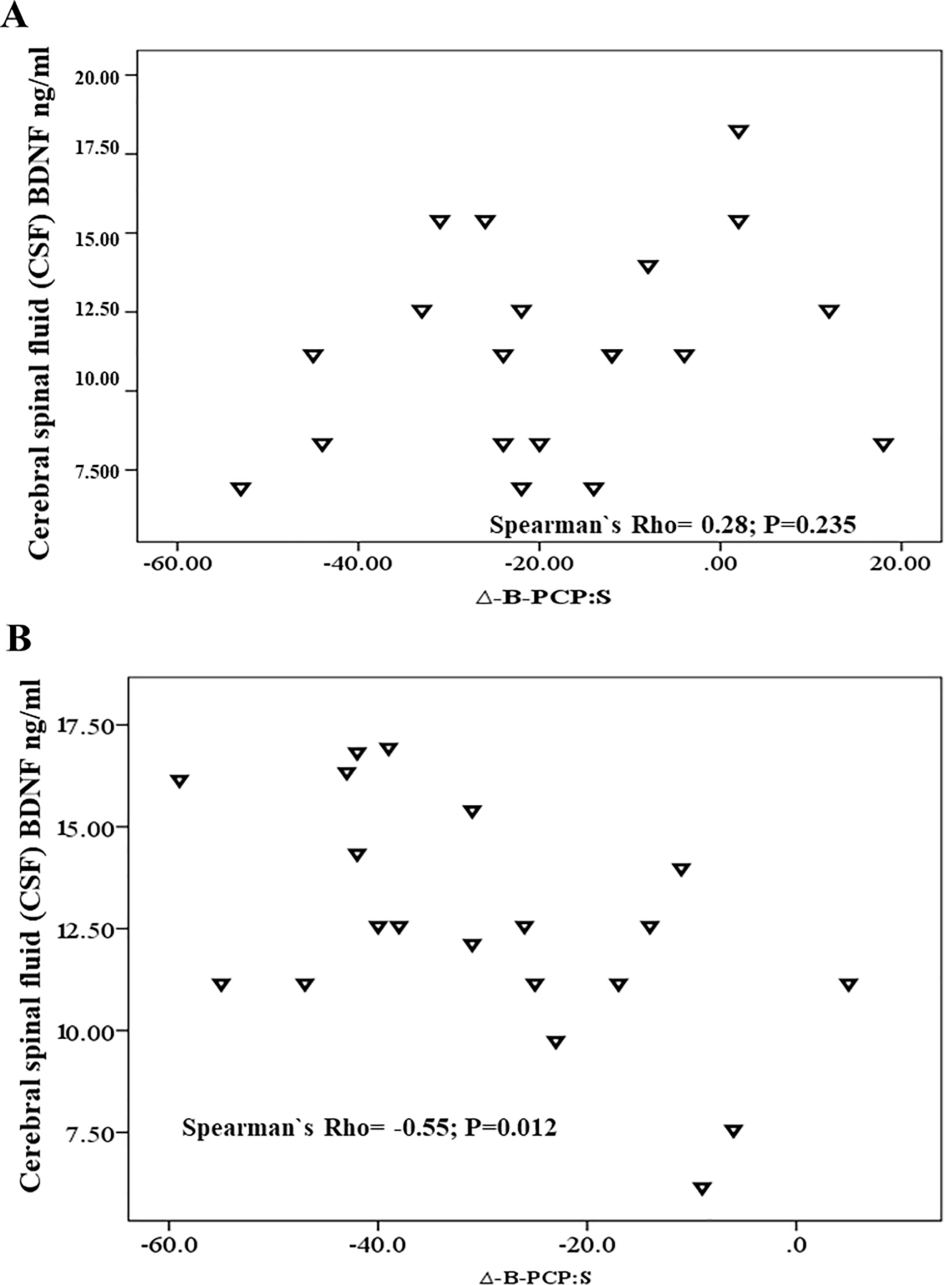
A and B. Scatter plots of CSF BDNF and Δ-B-PCP:S score value (i.e., follow-up end minus baseline level) according to s-tDCS (5A) and a-tDCS (5B).

The Scatter plots of the raw Δ-B-PCP:S and the CSF BDNF according to s-tDCS and a-tDCS group is presented in Fig 5A and 5B, respectively. The Δ-B-PCP:S and the CSF BDNF in the a-tDCS showed a positive non-parametric correlation between them. Such non-parametric correlation means that patients that received a-tDCS showed a greater Δ-B-PCP:S of CSF BDNF or vice-versa. The correlation coefficient between the CSF BDNF and the Δ-B-PCP:S score in the s-tDCS was Spearman`s Rho = 0.28, and its confidence interval (CI) 95% was (-18 to 0.64); P = 0.234. The correlation coefficient between the CSF BDNF and the Δ-B-PCP:S score in the a-tDCS was Spearman`s Rho = -0.55, and its confidence interval (CI) 95% was (-0.82 to -0.20); P = 0.012.

## Discussion

This study demonstrated that a-tDCS was superior to s-tDCS and improved postoperative pain and disability during the hallux valgus postoperative period. In addition, a-tDCS improved the corticospinal inhibitory system function and possibly its effect may influence BDNF secretion, which is associated with better recovery as assessed by DRP.

The tDCS effect on pain was demonstrated in the present study by different measures (i.e. reduction in VAS scores, lower analgesic consumption, improvement in descending pain modulatory system and DRP). It induced a large size effect on postoperative pain score in both moments, at rest and during walking. Although the exact mechanism of the tDCS effect involved in our outcomes is not clear, this investigation adds to the current literature, by providing evidence of the benefits of preoperative tDCS as an approach with potential clinical relevance for improving postoperative pain management and also by opening up the door to the investigation of other methods to enhance brain plasticity before surgery as a tool to improve post-operative outcomes.

Indeed, the effect observed in acute postoperative pain is corroborated by measurements related to neuroplasticity, which is less prone to assessment bias (i.e. CSF BDNF). Accordingly, this effect of tDCS can be extrapolated to patients with persistent preoperative pain. The clinical effect observed in this study is similar to results previously reported in several chronic pain conditions (i.e. fibromyalgia [27], neuropathic pain [28], etc.), as well as acute postoperative pain [16]. Despite the clinical efficacy of non-invasive methods of neuromodulation on pain has not been settled yet, as explored in several studies; according to a recent meta-analysis, the results of these studies are mixed and the level of evidence limited by the heterogeneity, small sample size and short follow-up [29].

In addition, the tDCS effect enhanced corticospinal inhibitory system as accessed by the NPS variation during the CPM-task, concurrent with a greater CSF BDNF level. The correlation between the a-tDCS effect and improvement in endogenous pain modulatory system dysfunction might provide insight to identify patients prone to develop increased postsurgical pain [30], and perhaps with a higher propensity for chronic postoperative pain. This finding is supported by convincing evidence that chronic pain is a maladaptive state of facilitation that involves disruption of descending modulatory systems [31]. Although we observed a lack of a statistically significant difference in BDNF means, either in the serum or in the CSF approximately four hours after the second session of tDCS, its effect was sustained beyond real intervention periods. The absence of difference between groups in CSF BDNF may be explained by a lack of power in this secondary analysis, because in the stratified analysis, an effect according to the group was demonstrated in pain and disability at follow-up end. Although we cannot determine that there is a cause-effect relationship based on this association between BDNF and DRP, we can interpret this as a gradient relationship, where the neuroplasticity process induced by preoperative tDCS continue throughout the postoperative period.

Whereas the underlying mechanisms of tDCS on DRP has not been fully elucidated, it is plausible that the relationship between CSF BDNF levels, postoperative pain and disability could be an indirect measurement of neuroplasticity changes induced by a-tDCS. In fact, present findings suggest that the direction of the modulation on pain and BDNF persisted beyond tDCS effects. Hence, the correlation between CSF BDNF and clinical effect provide some evidence that tDCS can induce changes in the excitatory/inhibitory balance in the central nervous system, which modulates the transmission of nociceptive inputs [32].

However, we need to interpret the a-tDCS effect with parsimony before claiming its effect on acute postoperative pain, because this sample comprises patients with persistent chronic pain associated with some disability, which may be more prone to its therapeutic effect. Accordingly, early evidence in animal and human studies suggest that tDCS downregulates facilitation of pain from sensitized neurons in the thalamus and brainstem nuclei structures [33] and so, interrupts disinhibition processes of the motor cortex excitability [5]. In fact, the tDCS effect depends on neural networks’ physiological state [34]. However, this issue needs to be better studied to define possible tDCS benefits, and if its impact can change according to chronic preoperative pain type (inflammatory, neuropathic, etc.).

One strength of this study is the internal validity of its design, in which all patients were submitted to the same type of stimulus (all patients were submitted to the same technique by the same surgeon) and evaluated by the same masked person (the anesthesiologist) and that tDCS is a non-invasive brain stimulation method that permits an appropriate masking, which was observed in the current study because all patients from the s-tDCS group guessed to be in the a-tDCS group. Another positive aspect is that we measured CSF BDNF, which permits an assessment to neurons and neuroglia secretion without accounting for possible interference by blood-brain barrier crossing [35]. However, we need to address several methodological limitations related to the study design. First, BDNF polymorphisms’ potential influence was not assessed, which might have an impact in neuronal plasticity. Secondly, study results may be relevant only to the investigated population, which excludes patients with American Society of Anesthesiologists Status higher than II. While homogeneous study population is methodologically advantageous, external validity issues arise. That is, hallux valgus may be associated with a particular psychological and behavioral state that is not common in other surgical populations since preoperative anxiety level is related to gender, age and surgery type [36]. Third, the heat pain threshold as conditioning pain stimulus to elicit a prolonged pain sensation to trigger CPM was distant from the source of ongoing pain because the persistent chronic pain activates the systems involved in pain processing. It induces a widespread central sensitization that explains the generalized hyperalgesia outside the pain source site, as observed in both animal [37] and humans, in many pain conditions (i.e. whiplash, fibromyalgia, low back pain, rheumatoid arthritis, endometriosis) [38]. In addition, the CPM-task is an element ascribed to processes within the central nervous system to assess the disruption of the balance of descending modulatory circuits to favor facilitation, which promotes and maintain the chronic pain [39]. Fourth, another factor to account in the interpretation of our findings is electrode positioning over the left M1 and the cathode over the right supra-orbital area independently to the side of the operated foot [11]. In our study, the anodal electrode was applied homolateral to the surgery side in 14 patients, who were equally distributed between the groups. However, we observed a clinical effect of the intervention indubitable significant, which makes the specification of the pain side not determinant to the tDCS effect. Taken together, these findings suggest that tDCS can cause changes in functional networks across the brain both within and beyond the motor system [33]. However, we cannot affirm if the stimulation of the contralateral hemisphere of the foot pain could change the effect size on pain. Finally, in the interpretation of these findings, we need to consider two important factors related to applying a sample size larger than the estimated for the primary outcomes’ analysis: a) this statistical approach was pre-planned to have a sufficient power to compensate for repeated observations; b) the multiple statistical tests may lead to inflation of the overall significance level [40]. Hence, the secondary outcomes would need confirmation to establish their validity in future trials.

In conclusion, according to this protocol, two preoperative anodal tDCS sessions improve postoperative pain control, as demonstrated by a reduction in pain scores, in analgesic use, in the disability related to pain and an enhancement in the function of the descending pain modulatory system. Also, these findings suggest that BDNF level in the CSF after the a-tDCS predicted the DRP at the end of one week after surgery. In overall, these results suggest that a-tDCS effects may be mediated by top-down regulatory mechanisms associated with the inhibitory cortical control.

## Supporting information

S1 File Database with main outcomes. (PDF)

S2 File CONSORT 2010 checklist. (PDF)

S3 File Protocol in English. (PDF)

S4 File Protocol in Portuguese. (PDF)
