# Supplementary material for: Preoperative transcranial direct current stimulation: Exploration of a novel strategy to enhance neuroplasticity before surgery to control postoperative pain. A randomized sham-controlled study
Source: PLoS One. 2017 Nov 30;12(11):e0187013. doi: 10.1371/journal.pone.0187013 (PMC5708693; doi:10.1371/journal.pone.0187013)
Supplement: S1 File — (PDF) [file pone.0187013.s001.pdf]

Patient	time	group	RA	ANALG	WB
1.00	0.00	1.00	2.00	3.00	6.00
2.00	0.00	0.00	8.00	3.00	5.00
3.00	0.00	0.00	3.00	2.00	2.00
4.00	0.00	0.00	7.00	4.00	0.00
5.00	0.00	1.00	3.00	4.00	7.00
6.00	0.00	0.00	6.00	2.00	7.00
7.00	0.00	1.00	3.00	3.00	9.00
8.00	0.00	0.00	0.00	4.00	0.00
9.00	0.00	1.00	3.00	4.00	8.00
10.00	0.00	1.00	0.00	2.00	4.00
11.00	0.00	0.00	5.00	3.00	5.00
12.00	0.00	0.00	2.00	3.00	6.00
13.00	0.00	0.00	2.00	0.00	2.00
14.00	0.00	1.00	0.00	2.00	5.00
15.00	0.00	1.00	5.00	3.00	5.00
16.00	0.00	0.00	2.00	4.00	4.00
17.00	0.00	0.00	3.00	0.00	7.00
18.00	0.00	1.00	0.00	4.00	7.00
19.00	0.00	1.00	3.00	4.00	7.00
20.00	0.00	0.00	8.00	3.00	8.00
21.00	0.00	#NULL!	2.00	3.00	4.00
22.00	0.00	1.00	0.00	3.00	0.00
23.00	0.00	0.00	2.00	3.00	5.00
24.00	0.00	1.00	0.00	0.00	2.00
25.00	0.00	0.00	0.00	4.00	0.00
26.00	0.00	1.00	5.00	0.00	8.00
27.00	0.00	1.00	0.00	0.00	0.00
28.00	0.00	0.00	3.00	3.00	2.00
29.00	0.00	0.00	5.00	3.00	7.00
30.00	0.00	0.00	2.00	4.00	6.00
31.00	0.00	1.00	5.00	4.00	10.00
32.00	0.00	0.00	0.00	3.00	8.00
33.00	0.00	1.00	0.00	4.00	3.00
34.00	0.00	0.00	3.00	4.00	7.00
35.00	0.00	1.00	3.00	3.00	4.00
36.00	0.00	1.00	4.00	3.00	7.00
37.00	0.00	0.00	0.00	0.00	3.00
38.00	0.00	1.00	0.00	4.00	0.00
39.00	0.00	0.00	3.00	2.00	5.00
40.00	0.00	1.00	5.00	2.00	6.00
1.00	1.00	1.00	0.00	4.00	4.00
2.00	1.00	0.00	3.00	0.00	3.00
3.00	1.00	0.00	0.00	4.00	0.00
4.00	1.00	0.00	4.00	4.00	6.00
5.00	1.00	1.00	5.00	3.00	6.00
6.00	1.00	0.00	0.00	3.00	5.00

7.00	1.00	1.00	4.00	3.00	7.00
8.00	1.00	0.00	0.00	3.00	0.00
9.00	1.00	1.00	0.00	0.00	0.00
10.00	1.00	1.00	0.00	4.00	0.00
11.00	1.00	0.00	2.00	0.00	3.00
12.00	1.00	0.00	1.00	0.00	1.00
13.00	1.00	0.00	2.00	3.00	2.00
14.00	1.00	1.00	0.00	3.00	0.00
15.00	1.00	1.00	4.00	4.00	4.00
16.00	1.00	0.00	0.00	4.00	0.00
17.00	1.00	0.00	0.00	3.00	0.00
18.00	1.00	1.00	0.00	4.00	0.00
19.00	1.00	1.00	0.00	4.00	0.00
20.00	1.00	0.00	0.00	3.00	0.00
21.00	1.00	1.00	1.00	3.00	3.00
22.00	1.00	1.00	0.00	0.00	0.00
23.00	1.00	0.00	5.00	4.00	5.00
24.00	1.00	1.00	0.00	2.00	0.00
25.00	1.00	0.00	3.00	0.00	0.00
26.00	1.00	1.00	5.00	1.00	5.00
27.00	1.00	1.00	0.00	2.00	0.00
28.00	1.00	0.00	0.00	4.00	0.00
29.00	1.00	0.00	0.00	3.00	0.00
30.00	1.00	0.00	0.00	1.00	0.00
31.00	1.00	1.00	4.00	2.00	7.00
32.00	1.00	0.00	4.00	0.00	4.00
33.00	1.00	1.00	0.00	0.00	0.00
34.00	1.00	0.00	3.00	1.00	4.00
35.00	1.00	1.00	0.00	2.00	3.00
36.00	1.00	1.00	2.00	2.00	2.00
37.00	1.00	0.00	0.00	3.00	1.00
38.00	1.00	1.00	0.00	2.00	0.00
39.00	1.00	0.00	0.00	1.00	0.00
40.00	1.00	1.00	4.00	1.00	4.00
1.00	2.00	1.00	3.00	3.00	10.00
2.00	2.00	0.00	4.00	3.00	8.00
3.00	2.00	0.00	4.00	4.00	8.00
4.00	2.00	0.00	2.00	2.00	10.00
5.00	2.00	1.00	0.00	0.00	0.00
6.00	2.00	0.00	6.00	5.00	10.00
7.00	2.00	1.00	4.00	4.00	10.00
8.00	2.00	0.00	6.00	3.00	10.00
9.00	2.00	1.00	0.00	0.00	0.00
10.00	2.00	1.00	3.00	5.00	8.00
11.00	2.00	0.00	7.00	3.00	7.00
12.00	2.00	0.00	6.00	3.00	10.00
13.00	2.00	0.00	3.00	1.00	8.00

14.00	2.00	1.00	0.00	1.00	6.00
15.00	2.00	1.00	5.00	6.00	6.00
16.00	2.00	0.00	6.00	1.00	6.00
17.00	2.00	0.00	6.00	2.00	8.00
18.00	2.00	1.00	0.00	1.00	4.00
19.00	2.00	1.00	3.00	0.00	5.00
20.00	2.00	0.00	6.00	2.00	10.00
21.00	2.00	1.00	3.00	3.00	7.00
22.00	2.00	1.00	5.00	4.00	10.00
23.00	2.00	0.00	8.00	3.00	10.00
24.00	2.00	1.00	0.00	0.00	8.00
25.00	2.00	0.00	7.00	4.00	8.00
26.00	2.00	1.00	3.00	4.00	10.00
27.00	2.00	1.00	2.00	2.00	8.00
28.00	2.00	0.00	6.00	4.00	10.00
29.00	2.00	0.00	6.00	5.00	10.00
30.00	2.00	0.00	8.00	5.00	10.00
31.00	2.00	1.00	3.00	3.00	7.00
32.00	2.00	0.00	7.00	3.00	10.00
33.00	2.00	1.00	3.00	3.00	3.00
34.00	2.00	0.00	7.00	5.00	10.00
35.00	2.00	1.00	4.00	7.00	10.00
36.00	2.00	1.00	2.00	3.00	5.00
37.00	2.00	0.00	3.00	3.00	4.00
38.00	2.00	1.00	0.00	3.00	3.00
39.00	2.00	0.00	5.00	1.00	9.00
40.00	2.00	1.00	4.00	3.00	10.00
1.00	3.00	1.00	3.00	2.00	8.00
2.00	3.00	0.00	7.00	6.00	10.00
3.00	3.00	0.00	4.00	4.00	10.00
4.00	3.00	0.00	8.00	4.00	10.00
5.00	3.00	1.00	3.00	4.00	6.00
6.00	3.00	0.00	9.00	6.00	10.00
7.00	3.00	1.00	4.00	4.00	10.00
8.00	3.00	0.00	4.00	6.00	9.00
9.00	3.00	1.00	3.00	5.00	9.00
10.00	3.00	1.00	4.00	5.00	5.00
11.00	3.00	0.00	1.00	1.00	2.00
12.00	3.00	0.00	3.00	3.00	7.00
13.00	3.00	0.00	8.00	3.00	9.00
14.00	3.00	1.00	0.00	4.00	5.00
15.00	3.00	1.00	4.00	5.00	6.00
16.00	3.00	0.00	7.00	4.00	9.00
17.00	3.00	0.00	7.00	5.00	10.00
18.00	3.00	1.00	3.00	0.00	0.00
19.00	3.00	1.00	3.00	1.00	4.00
20.00	3.00	0.00	4.00	5.00	6.00

21.00	3.00	1.00	3.00	2.00	4.00
22.00	3.00	1.00	3.00	4.00	6.00
23.00	3.00	0.00	8.00	7.00	9.00
24.00	3.00	1.00	0.00	2.00	5.00
25.00	3.00	0.00	6.00	3.00	8.00
26.00	3.00	1.00	4.00	4.00	7.00
27.00	3.00	1.00	5.00	4.00	2.00
28.00	3.00	0.00	7.00	5.00	10.00
29.00	3.00	0.00	4.00	6.00	6.00
30.00	3.00	0.00	5.00	3.00	6.00
31.00	3.00	1.00	6.00	1.00	7.00
32.00	3.00	0.00	9.00	5.00	9.00
33.00	3.00	1.00	3.00	4.00	8.00
34.00	3.00	0.00	5.00	5.00	7.00
35.00	3.00	1.00	3.00	7.00	8.00
36.00	3.00	1.00	2.00	4.00	9.00
37.00	3.00	0.00	4.00	4.00	5.00
38.00	3.00	1.00	0.00	3.00	0.00
39.00	3.00	0.00	6.00	2.00	8.00
40.00	3.00	1.00	5.00	3.00	8.00
1.00	4.00	1.00	2.00	1.00	5.00
2.00	4.00	0.00	4.00	5.00	8.00
3.00	4.00	0.00	4.00	1.00	6.00
4.00	4.00	0.00	7.00	5.00	10.00
5.00	4.00	1.00	0.00	0.00	6.00
6.00	4.00	0.00	8.00	4.00	9.00
7.00	4.00	1.00	0.00	4.00	4.00
8.00	4.00	0.00	4.00	5.00	4.00
9.00	4.00	1.00	0.00	0.00	0.00
10.00	4.00	1.00	0.00	5.00	0.00
11.00	4.00	0.00	1.00	0.00	2.00
12.00	4.00	0.00	3.00	4.00	4.00
13.00	4.00	0.00	3.00	1.00	5.00
14.00	4.00	1.00	0.00	1.00	3.00
15.00	4.00	1.00	4.00	2.00	6.00
16.00	4.00	0.00	0.00	4.00	2.00
17.00	4.00	0.00	5.00	4.00	7.00
18.00	4.00	1.00	0.00	0.00	0.00
19.00	4.00	1.00	0.00	0.00	1.00
20.00	4.00	0.00	4.00	4.00	6.00
21.00	4.00	1.00	0.00	0.00	2.00
22.00	4.00	1.00	3.00	2.00	5.00
23.00	4.00	0.00	4.00	4.00	7.00
24.00	4.00	1.00	0.00	0.00	4.00
25.00	4.00	0.00	4.00	1.00	7.00
26.00	4.00	1.00	3.00	3.00	8.00
27.00	4.00	1.00	0.00	0.00	0.00

28.00	4.00	0.00	7.00	5.00	8.00
29.00	4.00	0.00	5.00	1.00	7.00
30.00	4.00	0.00	5.00	1.00	7.00
31.00	4.00	1.00	3.00	3.00	6.00
32.00	4.00	0.00	7.00	1.00	8.00
33.00	4.00	1.00	3.00	4.00	4.00
34.00	4.00	0.00	3.00	1.00	4.00
35.00	4.00	1.00	4.00	4.00	7.00
36.00	4.00	1.00	2.00	1.00	3.00
37.00	4.00	0.00	2.00	4.00	2.00
38.00	4.00	1.00	1.00	2.00	2.00
39.00	4.00	0.00	5.00	2.00	7.00
40.00	4.00	1.00	0.00	1.00	7.00
1.00	5.00	1.00	2.00	0.00	5.00
2.00	5.00	0.00	2.00	2.00	5.00
3.00	5.00	0.00	0.00	5.00	0.00
4.00	5.00	0.00	7.00	8.00	10.00
5.00	5.00	1.00	2.00	2.00	2.00
6.00	5.00	0.00	6.00	1.00	8.00
7.00	5.00	1.00	4.00	4.00	5.00
8.00	5.00	0.00	4.00	4.00	5.00
9.00	5.00	1.00	0.00	1.00	1.00
10.00	5.00	1.00	1.00	0.00	2.00
11.00	5.00	0.00	0.00	0.00	1.00
12.00	5.00	0.00	3.00	3.00	6.00
13.00	5.00	0.00	6.00	3.00	8.00
14.00	5.00	1.00	0.00	1.00	0.00
15.00	5.00	1.00	3.00	4.00	4.00
16.00	5.00	0.00	0.00	3.00	2.00
17.00	5.00	0.00	5.00	3.00	7.00
18.00	5.00	1.00	0.00	0.00	0.00
19.00	5.00	1.00	3.00	0.00	0.00
20.00	5.00	0.00	8.00	5.00	8.00
21.00	5.00	1.00	0.00	0.00	2.00
22.00	5.00	1.00	3.00	3.00	3.00
23.00	5.00	0.00	6.00	3.00	4.00
24.00	5.00	1.00	0.00	0.00	1.00
25.00	5.00	0.00	5.00	1.00	4.00
26.00	5.00	1.00	3.00	4.00	2.00
27.00	5.00	1.00	0.00	0.00	0.00
28.00	5.00	0.00	6.00	0.00	5.00
29.00	5.00	0.00	4.00	2.00	6.00
30.00	5.00	0.00	5.00	1.00	7.00
31.00	5.00	1.00	4.00	2.00	4.00
32.00	5.00	0.00	7.00	1.00	8.00
33.00	5.00	1.00	0.00	2.00	3.00
34.00	5.00	0.00	3.00	0.00	3.00

35.00	5.00	1.00	3.00	3.00	3.00
36.00	5.00	1.00	0.00	1.00	4.00
37.00	5.00	0.00	5.00	4.00	5.00
38.00	5.00	1.00	4.00	2.00	2.00
39.00	5.00	0.00	6.00	2.00	5.00
40.00	5.00	1.00	0.00	0.00	3.00
1.00	6.00	1.00	1.00	0.00	2.00
2.00	6.00	0.00	5.00	5.00	5.00
3.00	6.00	0.00	5.00	0.00	4.00
4.00	6.00	0.00	5.00	6.00	2.00
5.00	6.00	1.00	2.00	5.00	3.00
6.00	6.00	0.00	3.00	0.00	4.00
7.00	6.00	1.00	0.00	4.00	2.00
8.00	6.00	0.00	0.00	4.00	0.00
9.00	6.00	1.00	3.00	2.00	1.00
10.00	6.00	1.00	0.00	0.00	0.00
11.00	6.00	0.00	5.00	0.00	1.00
12.00	6.00	0.00	7.00	4.00	7.00
13.00	6.00	0.00	5.00	1.00	5.00
14.00	6.00	1.00	0.00	0.00	0.00
15.00	6.00	1.00	4.00	0.00	5.00
16.00	6.00	0.00	0.00	2.00	2.00
17.00	6.00	0.00	3.00	3.00	5.00
18.00	6.00	1.00	0.00	0.00	0.00
19.00	6.00	1.00	0.00	0.00	0.00
20.00	6.00	0.00	5.00	0.00	0.00
21.00	6.00	1.00	0.00	0.00	1.00
22.00	6.00	1.00	2.00	1.00	3.00
23.00	6.00	0.00	1.00	2.00	2.00
24.00	6.00	1.00	0.00	0.00	1.00
25.00	6.00	0.00	5.00	1.00	7.00
26.00	6.00	1.00	3.00	2.00	5.00
27.00	6.00	1.00	0.00	0.00	0.00
28.00	6.00	0.00	4.00	0.00	4.00
29.00	6.00	0.00	4.00	3.00	7.00
30.00	6.00	0.00	5.00	0.00	7.00
31.00	6.00	1.00	0.00	3.00	3.00
32.00	6.00	0.00	0.00	0.00	0.00
33.00	6.00	1.00	0.00	4.00	3.00
34.00	6.00	0.00	5.00	1.00	6.00
35.00	6.00	1.00	3.00	3.00	5.00
36.00	6.00	1.00	0.00	0.00	1.00
37.00	6.00	0.00	2.00	3.00	3.00
38.00	6.00	1.00	0.00	2.00	0.00
39.00	6.00	0.00	5.00	1.00	8.00
40.00	6.00	1.00	3.00	1.00	5.00
1.00	7.00	1.00	1.00	0.00	3.00

2.00	7.00	0.00	1.00	3.00	3.00
3.00	7.00	0.00	0.00	0.00	0.00
4.00	7.00	0.00	6.00	7.00	6.00
5.00	7.00	1.00	2.00	1.00	2.00
6.00	7.00	0.00	3.00	0.00	3.00
7.00	7.00	1.00	0.00	0.00	2.00
8.00	7.00	0.00	0.00	2.00	2.00
9.00	7.00	1.00	5.00	6.00	5.00
10.00	7.00	1.00	0.00	0.00	0.00
11.00	7.00	0.00	0.00	0.00	3.00
12.00	7.00	0.00	3.00	3.00	5.00
13.00	7.00	0.00	2.00	0.00	4.00
14.00	7.00	1.00	0.00	0.00	0.00
15.00	7.00	1.00	3.00	1.00	4.00
16.00	7.00	0.00	0.00	0.00	3.00
17.00	7.00	0.00	9.00	2.00	10.00
18.00	7.00	1.00	0.00	0.00	0.00
19.00	7.00	1.00	0.00	0.00	0.00
20.00	7.00	0.00	0.00	0.00	0.00
21.00	7.00	1.00	0.00	0.00	2.00
22.00	7.00	1.00	1.00	0.00	3.00
23.00	7.00	0.00	1.00	3.00	2.00
24.00	7.00	1.00	0.00	0.00	0.00
25.00	7.00	0.00	3.00	1.00	5.00
26.00	7.00	1.00	3.00	3.00	4.00
27.00	7.00	1.00	0.00	0.00	0.00
28.00	7.00	0.00	3.00	0.00	5.00
29.00	7.00	0.00	4.00	3.00	8.00
30.00	7.00	0.00	3.00	0.00	7.00
31.00	7.00	1.00	3.00	0.00	0.00
32.00	7.00	0.00	0.00	0.00	4.00
33.00	7.00	1.00	0.00	1.00	2.00
34.00	7.00	0.00	6.00	2.00	7.00
35.00	7.00	1.00	3.00	4.00	5.00
36.00	7.00	1.00	0.00	1.00	3.00
37.00	7.00	0.00	2.00	3.00	5.00
38.00	7.00	1.00	0.00	1.00	1.00
39.00	7.00	0.00	4.00	1.00	7.00
40.00	7.00	1.00	0.00	1.00	4.00
