# Supplementary material for: Preoperative transcranial direct current stimulation: Exploration of a novel strategy to enhance neuroplasticity before surgery to control postoperative pain. A randomized sham-controlled study
Source: PLoS One. 2017 Nov 30;12(11):e0187013. doi: 10.1371/journal.pone.0187013 (PMC5708693; doi:10.1371/journal.pone.0187013)
Supplement: S3 File — (PDF) [file pone.0187013.s003.pdf]

FEDERAL UNIVERSITY OF RIO GRANDE DO SUL

COLLEGE OF MEDICINE

POSTGRADUATE MEDICINE PROGRAM: MEDICAL SCIENCES

PROJECT

THE EFFECT OF TRANSCRANIAL DIRECT CURRENT STIMULATION (TDCS) IN THE PRE-OPERATIVE PERIOD OF SURGICAL TREATMENT OF HALLUX VALGUS

HUGO DANIEL WELTER RIBEIRO

Doctor graduated from the Federal University of Paraná - UFPR. Anesthesiologist by the Center of Education in Anesthesiology and Perioperative Medicine of the Hospital of Clinics of Porto Alegre

MENTOR

Prof. Wolnei Caumo, M.D., PhD.

Porto Alegre - 2014

FIGURE LIST

Figure 1 - Strategy for searching for bibliographic references of the bases of the study objectives.. 08

Figure 2. Chevron Technique Illustration..09

Figure 3. Illustration of Akin's technique..10

ABBREVIATIONS LIST

1st MPJ = first metatarsal phalangeal joint

AMP = Adenosine Monophosphate

BDNF = brain derived neurotrophic factor

B-PCP:S = Brazilian Profile of Chronic Pain: Screen

CONSORT = Consolidated Standards of Reporting Trials

CPM = conditioned pain modulation

HCPA = Hospital de Clínicas of Porto Alegre

LTP = long-term potentiation

LTD = long-term depression

NHC = National Health Council

NGF = Neural Growth Factor

NPS = numerical pain scale

QST = quantitative sensory test

rTMS = repeated transcranial magnetic stimulation

STAI = state and trait anxiety index

tDCS = transcranial Direct Current Stimulation

ABSTRACT

Introduction: Forefoot deformities are important cause of feet pain and discomfort and affect around 80% of general population, predominantly in female population. Among teenagers and adults´ common forefoot deformities is hallux valgus. To achieve the deformity heal, surgical treatment is needed. The transcranial direct current stimulation (tDCS) is a non invasive technic, that aims for the central nervous system modulation for pain control, which can become a therapeutic option for postoperative pain. Experimental studies have shown that tDCS has reverted the hyperalgesia induced by chronic stress and articular inflammatory pain, has reduced total knee arthroplasty postoperative opioid consumption and a single 20 minutes session has shown reduction in endoscopic procedures opioid consumption, safety and minimal adverse effects. However, the tDCS effect in preoperative hyperalgesia has not been explored yet in sensitized patients, neither in anxiety and postoperative rehabilitation of patients submitted to hallux valgus surgical correction. In this study, it was chosen to use tDCS to stimulate cerebral cortex due to its efficacy in painful syndromes, for being a non invasive, low cost and easy-to-apply technic in comparison to other neurostimulation technics and especially, for its potential to counter-regulate the mal adaptive neuroplastic alterations associated to chronic pain. **Objective:** to evaluate the effect of tDCS compared to tDCS-sham in the pain control (visual analog scale score, pain threshold and the descendent modulator system), perioperative anxiety, postoperative analgesic drug consumption and in the rehabilitation of patients with arthralgia of the first metatarsophalangeal articulation submitted to hallux valgus surgical correction. **Method:** it is a randomized, blinded, placebo-sham controlled clinical trial which includes 40 female patients, between 18 and 70 years old, candidates to hallux valgus surgical treatment by combined Chevron + Akin osteotomy due to arthralgia of the first metatarsophalangeal articulation. The patients will be randomized and divided into two groups that will be treated with two tDCS or tDCS-sham sessions of 20 minutes each in preoperative period. **Expected results:** This study will evaluate the effect of tDCS as a treatment option to postoperative pain and perioperative anxiety of patients submitted to hallux valgus surgical correction. In case of proven efficacy, this technic can become a low cost, easy access, safe and effective treatment option for these patients.

1. Introduction

Forefoot deformities are important causes of pain and discomfort for the feet. About 80% of the population has some form of pain or callosity during their lives [1, 2] and the incidence is markedly higher in the female population, suggesting a possible relationship with the type of female footwear [1]. When there is a failure in the anatomical and functional balance established between the various static and dynamic structures of the foot, metatarsalgia occurs, the etiology of which is related to the biomechanical changes of the foot in approximately 92% of the cases [1, 2].

During walking, the load on the foot is mainly concentrated in the three medial metatarsal bones. Based on that, the study by Cavanagh et al.[3] used computed electronic methods, which in fact confirmed that the load distributions under static conditions have a clear predominance in the central metatarsals, especially in the 2nd and 3rd rays [2, 3].

Among the common forefoot deformities of adolescents and adults is the hallux valgus. This abnormality can cause pain on the medial eminence of the head of the first metatarsus, usually related to the use of constrictive shoes, which induce the formation of bursitis due to excessive friction with eminence. In this type of pathology, the deformity may initially be flexible, becoming progressively more rigid and painful over time. Some patients with deformity may adapt their shoes to a more favorable shape, reducing the symptoms and thus, making possible the conservative approach of the hallux valgus. For this purpose, shoes with a wide and high anterior chamber may be the most effective. In the market there are numerous devices with the intention to protect the bunion, but none of them is able to correct the deformity in a definitive way. Thus, in order to achieve the cure of the deformity, surgical treatment is necessary.

One of the major components of a hallux valgus deformity is the size of the medial eminence, often reported as the focus of pain and intolerance to wearing shoes. It is suggested that the medial eminence is not a new growth, but rather a portion of the metatarsus that becomes exposed with the lateral deviation of the proximal phalanx of the affected foot. [4]. Cases of more severe deformities of the hallux valgus may result in pain and additional deformities in the lateral rays of the affected one. Thus, there may be pain and plantar callosity on the heads of the 2nd and 3rd metatarsals, which are generated by the functional loss of the first metatarsophalangeal joint [4]. After a long period of overloading, the plantar plaque complex overlying the metatarsal heads may collapse,

leading to imbalance between the flexor and extensor forces of the fingers and the consequent formation of claw toes.

The arthralgia of the first metatarsophalangeal joint (1st MPJ) is characterized by great suffering and by an expressive emotional load. Considering that chronic pain is defined as a condition associated with maladaptive neuroplasticity and not a simple reaction to certain types of stimuli, we treat it as a complex experience, a multidimensional phenomenon that includes cognitive, sensory-discriminative, emotional and motivational components, mediated by different mechanisms and processed in an extensive cortical network [5, 6]. The circuits involved in the processing of pain at cortical and subcortical levels, as well as the cellular and molecular mechanisms underlying this processing have made substantial progress in the last decades. This evolution of the neurobiological mechanisms' knowledge, combined with the concept that chronic pain is a process that involves maladaptive neuroplasticity, allowed the brain together with the spinal cord to be considered an active system that filters, selects and modulates the nociceptive afferents. In addition, with the improvement in imaging technology, it was possible to identify regions that are constantly activated in the acute nociceptive process, such as primary and secondary somatosensory cortex, insular cortex, prefrontal cortex, anterior cingulate, and thalamus [7-9].

Based on this knowledge, therapeutic options that aim on modulating the central nervous system to control pain have been more frequently studied. One of the techniques that has been studied for the treatment of chronic pain is transcranial direct current stimulation (tDCS) [10-17]. This technique uses low intensity continuous current (1-2 mA) (1-2 mA) [18] directed to the scalp via cathode and anode electrodes, capable of producing changes in cortical excitability, with longevity of effects depending on the duration of stimulation. It produces changes both in areas close to the electrodes, and in distant areas that have connection to the primarily stimulated area. [19] Its effects are dependent on polarity: anodic stimulation induces cortical excitability and cathodic stimulation reduces it. The effects are explained by the modulation of the neuronal membrane potential bound to the stimulated area [20] and the reduction of neuronal excitability occurs by the reduction of spontaneous cellular activity [21]. This effect lasts even after the end of the stimulation. The duration of the changes depends on the time and intensity of stimulation [22] and is thought to be additive to the repetition of stimulation courses. The long-term effect of tDCS is due to increased pre-synaptic input

and up-regulation of the synaptic tone mediated by NMDA receptor and dependent on protein synthesis, changes in intracellular cyclic AMP concentration and intracellular calcium current influx. These processes are part of the phenomena of long-term potentiation (LTP) and long-term depression (LTD) [19, 21-24]. LTP corresponds to a process of facilitation of the nervous system while the LTD weakens the synaptic transmission. Long-term changes in excitability resemble long-term potentiation or depression of the intensity of glutamatergic synapses [10, 16, 17, 21, 25].

Evidence suggests that the application of tDCS in the primary motor cortex modulates pain through direct cortical effects in the anterior and ventro-lateral thalamic nuclei, medial thalamus, anterior cingulate gyrus and trunk [26, 27]. Preliminary clinical trials have suggested that anodal tDCS of the primary motor cortex may be effective in the treatment of chronic pain syndromes such as fibromyalgia [28], spinal cord injury [12] and chronic pelvic pain [29, 30]. In addition, the tDCS produced an increase in pain thresholds in an experimental pain model [31]. In experimental studies with animals conducted by our group, it was observed that tDCS reversed the hyperalgesia induced by chronic stress and joint inflammatory pain [32]. However, the effect of tDCS on the hyperalgesic state of patients with chronic pain, as well as its potential effect on postoperative anxiety and rehabilitation were not explored.

In the perioperative context, small studies have demonstrated the potential benefit of neurostimulation techniques. As an example, there is a clinical trial that evaluated the effect of a single repetitive transcranial magnetic stimulation (rTMS) session in patients undergoing gastric bypass, which showed that treated patients had reduction in opioid consumption in the postoperative period[33]. The reduction in the consumption of opioids with the use of tDCS has been demonstrated in lumbar spine surgeries [34] and in the postoperative period of total knee arthroplasty [35]. A single 20-minute session had an impact on the reduction of opioid consumption in the postoperative period of endoscopic procedures [36]. The incidence of adverse effects of tDCS is low, and includes: headache, dizziness, nausea, itching sensation, erythema or cutaneous irritation at the electrode application site [18]. However, the effect of tDCS on preoperative hyperalgesia of sensitized patients, on anxiety and on postoperative rehabilitation of patients undergoing surgery for correction of halux valgus has not yet been explored. In this study we chose the use of tDCS to stimulate the cerebral cortex because of its demonstrated efficacy in pain syndromes, because it is a non-invasive technique, low cost, easy to apply technic

compared to other neurostimulation techniques and, above all, due to its potential to counter-regulate the maladaptive neuroplastic changes associated with chronic pain.

Considering the accumulation of evidence about the benefits of this technique and the low potential for adverse effects, new researches should be supported in order to obtain data that provide its correct indication, especially in patients sensitized by sustained pain. Our hypothesis is that the tDCS can help in the treatment of postoperative pain (VAS, pain threshold and CPM) of surgical correction of hallux valgus, in the reduction of preoperative anxiety and in the course of postoperative rehabilitation.

2. Literature review

2.1 Strategies for locating and selecting information

In this literature review, we intend to address some aspects about the relationship between chronic pain in patients with hallux valgus, its surgical treatment, and tDCS. The search strategy involved the following databases: MEDLINE (PubMed site), LILACS, SCIELO, and the Brazilian Digital Library of Theses and Dissertations. Orthopedic textbooks were also consulted.

The bibliographic references of the identified articles were revised to locate others not contemplated in the search. In the PubMed, LILACS and SCIELO sites we searched the terms hallux valgus, bunion pain, metatarsal head osteotomy, post operative pain, pre operative period and tDCS. In relation to the term hallux valgus, 3074 articles were found in PUBMED, 108 in LILACS and 21 in SCIELO. Using the term bunion pain was found 4 articles in PUBMED, 9 in LILACS and none in SCIELO; the term metatarsal head osteotomy, 321 articles in PUBMED, none in LILACS or SCIELO; the term post operative pain, 72313 articles in PUBMED, none in LILACS and 166 in SCIELO; the term pre operative period, 5156 articles in PUBMED, none in LILACS and 206 in SCIELO and in relation to tDCS were found 1277 in PUBMED, 10 in LILACS and 07 in SCIELO.

Refining the search, matching the keywords hallux valgus and metatarsal head osteotomy, a pool of 223 articles was generated in PUBMED, 1 article in LILACS and none in SCIELO. From the matching of the terms tDCS and bunion pain, from the terms tDCS and metatarsal head osteotomy, and from the terms tDCS and preoperative period, no articles were found in any of the databases. Matching tDCS and post operative pain we

got 6 articles in PUBMED and none in the other databases. From the thesis database, 19 theses with the tDCS theme were located. The systematic review was outlined in figure 1.

Figure 1 - Strategy for searching for bibliographic references of the bases of the study objectives.

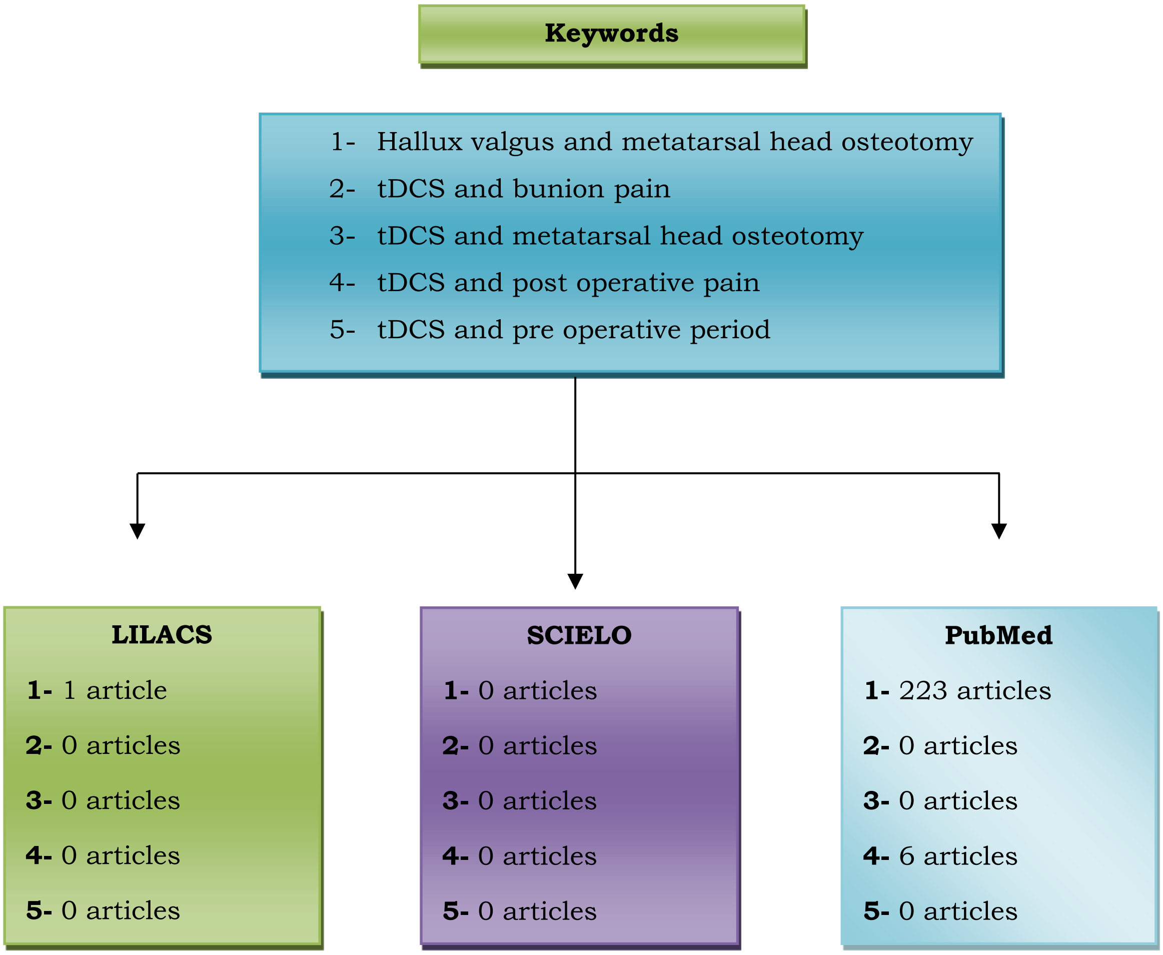

2.2 Historical and conceptual aspects

Hallux valgus is a common problem affecting the forefoot, in which the 1st MPJ progressively subluxes, with lateral deviation of the hallux and medial deviation of the first metatarsal [37]. It is often accompanied by painful medial prominence on the head of the first metatarsal, known as a bunion. As the deformity progresses, the lateral deviation of the hallux interferes in the alignment and dynamics of the smaller fingers, resulting in claw deformities, abnormal distribution of the plantar loads and development of plantar callosities. The pressure exerted by the medial prominence in the shoes can lead to the formation of adventitious bursitis, usually painful and inflamed. The cause of

the hallux valgus is not absolutely understood. There is some evidence that this condition is autosomal dominant. Other factors that may contribute are the use of constrictive shoes and structural factors of the foot.[37]

2.3 Therapeutic approach of metatarsal-phalangeal arthralgia

Some patients with hallux valgus tolerate the pain generated by the presence of the deformity when adjusting their shoes to a more favorable shape. Footwear with a wide and high anterior chamber may be the most effective for this purpose. Numerous devices with the purpose of protecting the deformity have already been made available in the market with the purpose of reducing the symptoms, making possible the conservative approach, but none of them is able to correct the deformity definitively. Thus, in order to achieve the cure of the deformity, surgical treatment is necessary.

The first reports of a distal osteotomy of the metatarsal head are from 1881, by Riverdin, who described a subcapital osteotomy with a wedge of subtraction for the correction of the hallux valgus [38]. Chevron osteotomy is one of the most common techniques for the surgical treatment of mild and moderate hallux valgus (Figure 2). No need for fixation was mentioned in his first reports. Thus, it was suggested that the shape of the osteotomy and the impaction of the spongy head on the axis of the first metatarsus would provide sufficient stability[39]. This technique is contraindicated in those patients who have arthrosis of the 1st MPJ of the affected foot and have metatarsal-cuneiform instability.

Figure 2. Chevron Technique Illustration

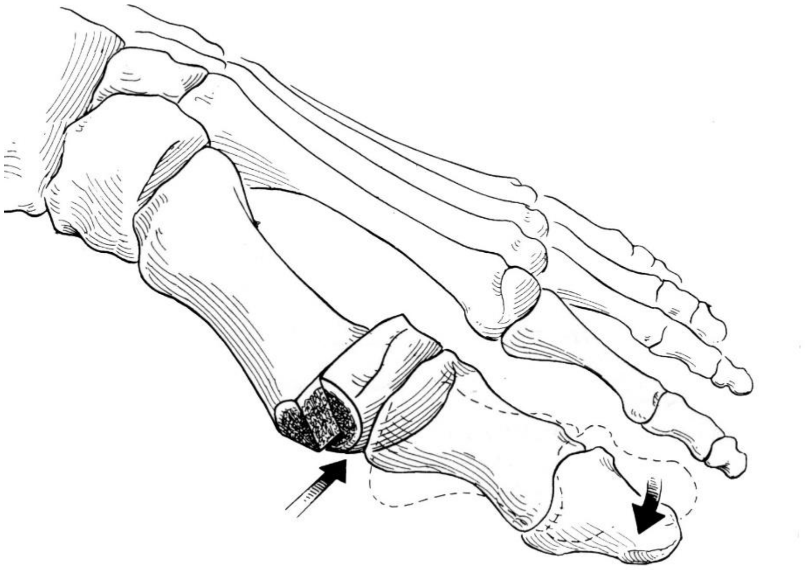

Later, to increase the indication for this technically simple osteotomy, were added internal fixation, association with release of the lateral structures of the metatarsal head and other concomitant osteotomies [40]. Optionally, in order to "potentiate" the correction of the hallux valgus, a second osteotomy, the Akin osteotomy, is associated with the proximal hallux phalangeal, subtracting a wedge with a medial base. (Figure 3)

Figure 3. Illustration of Akin's technique

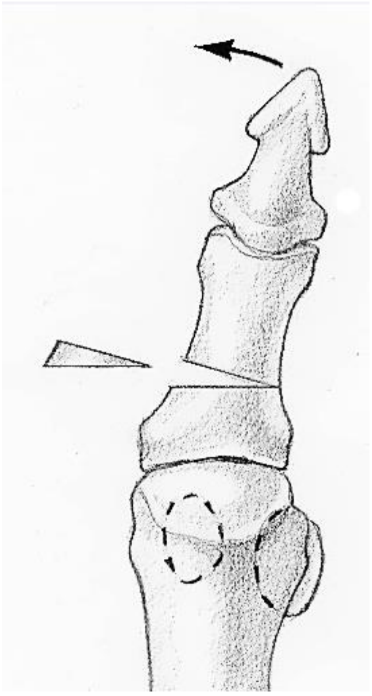

The Akin osteotomy, also performed with a single medial longitudinal incision, allows alignment on the hallux axis even when there is a marked lateral inclination of the head relative to the diaphysis of the first metatarsus. In these cases, the axis of the hallux is corrected spuriously, but the clinical result is largely satisfactory, both from the aesthetic and functional point of view. The combination of these techniques also allows correction of the hallux rotational deformity. During execution of the transverse osteotomy at the base of the proximal phalanx, it is possible to rotate the hallux in the opposite direction and correct excessive pronation[41].

Postoperative care and the recovery period of the combined techniques are similar to that of the isolated Chevron technique. In the study by Costa et al.[42] The combination of both techniques was used in the surgical treatment of hallux valgus in 29 patients (47 hallux valgus), which showed satisfactory clinical and functional results in 46 hallux valgus after a mean follow-up of 60 months.

2.4 Surgical technique

Both osteotomies (Chevron and Akin) are performed by the same incision, which is applied on the medial part of the forefoot, from the hallux proximal phalanx to the distal third of the first metatarsus. Planes careful dissection and excision of the spare joint capsule are then performed. This way, the medial metatarsal head exostosis, which must be removed in alignment with the medial border of the foot, becomes visible.

With exostosis removed, the Chevron osteotomy is performed distal to the metatarsal head, which can be fixed with the most diverse implants. In our service, we usually use cortical screws with a thickness of 2 mm for such anchoring.

In the most distal region of the incision, the proximal phalanx of the hallux is approached in a subperiosteal plane, protecting the flexor and extensor tendons long of the hallux. Using a oscillating saw, a wedge of medial base is removed with sufficient thickness to complete the correction of the hallux valgus, making the hallux completely aligned with the first radius. Subsequently, fixation with the same type of implant is performed.

In fact, greater care should be taken when suturing and retensioning the joint capsule of the first metatarsal. The excessive tension can generate mobility restriction and arthrosis, while insufficient tension can facilitate the recurrence of the deformity.[41-43]

2.5 Neuroplasticity - Process and markers

Patients with chronic pain undergo nervous system changes in the location and expression of ion channels, receptors and nerve synapses. Changes in the distribution and kinetics of neurotransmitters and neuromediators allow central or peripheral neurons to reach the threshold for depolarization earlier, generating ectopic discharges that amplify and activate neighboring cells. It may be inferred, then, that chronic pain is a state of constant facilitation of nerve conduction, when previously innocuous stimuli can be interpreted as pain (allodynia) or when the response to painful stimuli is not proportional to the intensity of aggression (hyperalgesia)[44]. This process involves mechanisms of neuroplasticity such as dendritic formation, synaptic remodeling, LTP, axonal development, neuritic extension, synaptogenesis and neurogenesis, by which the brain adapts and responds to a variety of internal and external stimuli [45]. Thus, chronic arthralgia of the first metatarsal-phalangeal joint induced by hallux valgus is a field that

can be explored through modulatory techniques that have significant and long-lasting effects.

Neural Growth Factor (NGF) was first identified as a survival factor for sensory and sympathetic neurons in the development of the nervous system. In adults, it is not necessary for neural survival, but it plays an important role in the generation of acute pain and hyperalgesia and in other chronic pain processes. The expression of NGF is intense in injured and inflamed tissues and triggers in nociceptive neurons the potentiation of pain signaling by several mechanisms. NGF is a founding member of the neurotrophin protein family structurally related to brain-derived neurotrophic factor (BDNF), neurotrophin 3 (NT-3) and NT-4. Another protein that acts as a neurotrophic factor evidencing central nervous system injury is S100 β, a marker that plays a role in energy metabolism, regulation of the cycle, growth, differentiation and cellular motility[46]. S100 β exerts trophic or toxic effects, depending on its concentration. At minimal concentrations, it stimulates neuronal growth, increases neuron survival during development and acts as a neurotrophic factor and neuronal survival protein, in response to glial activation secondary to injury. On the other hand, in high concentrations S100 β exacerbates neuroinflammation and neuronal dysfunction, with stimulation of proinflammatory cytokines and induction of apoptosis[47]. Studies have shown that this neurotrophic factor may be implicated in depression and in cognitive performance and pain [47-49]. BDNF is a neurotrophin abundantly expressed in adult brain and spinal cord, playing an important role in the regulation of inflammatory pain threshold and secondary hyperalgesia [50]. BDNF is not defined as an exclusive inducer of inhibition (GABA) or neuronal excitability (glutamate), since it exerts a modulatory role in activity-dependent neurotransmission [51]. Thus, this neurotrophin involved in the process of neuronal plasticity, participates in the process of long-lasting synaptic potentiation (LTP), a neuroplasticity mechanism that supports the pain memory process [52]. In addition, BDNF has diffuse effects on the monoaminergic neurotransmission system. It promotes sprouting of mature serotonergic neurons, increasing central serotonergic activity following infusion of this, modulating the activation of dorsal raphe nucleus neurons and serotonergic transporter function. BDNF also influences dopaminergic activity.

This set of evidence demonstrates that the establishment of chronic pain is mediated by multiple mechanisms that induce a series of medium and long-term adaptations, involving neural and endocrine modifications [53]. It should also be noted

that these factors can regulate or alter the release of glutamate and GABA in painful processes [54]. The synthesis of these neurotransmitters is caused by the isoenzyme glutamic acid decarboxylase (GAD) [55], which synthesizes from glutamate and pyridoxa-5'-phosphate (PLP) as cofactor, the neurotransmitter GABA [56].

2.6 Neuroplasticity and neurostimulation effect

To understand the effect of different therapies in the neuroplasticity process, we will use physiological parameters (pain-modulators), and neuroplasticity markers such as BDNF (neuronal plasticity). BDNF regulates the integrity and differentiation of neurons during development, including the process of neuronal plasticity. BDNF expression in the central nervous system is modified by diverse assumptions, such as stress, ischemia, hypoglycemia, depression and pain. BDNF has important synaptic effects: it increases the spontaneous frequency of action potentials in neurons, potentiates inhibitory and excitatory circuits, interferes in the neuromodulation of gabaergic, cholinergic, dopaminergic and noradrenergic inter-neurons.[57]. This neurotrophin acts on essential adaptive mechanisms, playing a crucial role in the process of long-lasting synaptic potentiation (LTP), a mechanism of fundamental neuroplasticity to trigger and sustain the process of pain memory. The increase of BDNF increases the LTP, while the reduction of its levels attenuates this phenomenon. Therefore, the relationship of serum levels with disease severity can confirm the systemic influence of this biomarker in states of sustained pain.

The potential advantages of non-invasive neuromodulatory therapies, besides being more effective and inexpensive, may constitute an additional therapeutic modality to be made available to society. However, it is important to evaluate its effects using evidence derived from the clinical effect combined with information from sources like plasma, psychophysical and electrophysiological markers. This study will add in the literature information on the composition of cerebral spinal fluid in patients submitted to neuromodulatory therapies.

2.7 Transcranial Direct Current Stimulation (tDCS) and metatarsal-phalangeal arthralgia

The tDCS is a low-cost, non-invasive, and easy-to-operate brain stimulation technique compared to other neuromodulation techniques. It consists of the application of a direct current on the scalp using electrodes that are surrounded by perforated sponges

moistened with saline or conductive gel rubber electrodes [58]. Its effects depend on the size, polarity (anodic or cathodic) and position of the electrodes, intensity of the applied current, duration of the stimulus and the properties of the tissue of the stimulated area. Anodic stimulation increases cortical excitability, while cathodal stimulation decreases it [18]. The mechanism of action of tDCS is multifactorial and induces physiological changes in different systems, located or distant from the focus of application. Some exploratory studies have identified to date different effects of tDCS on neurotransmission, neurochemical pathways and neurobiological markers such as the modulation of glutamatergic activity [59, 60], GABAergic [61, 62], dopaminergic [63-65], serotonergic [66] and cholinergic [67, 68], besides the effect on the permeability of different membrane channels such as sodium and calcium. Its benefit has already been demonstrated in several pathological conditions such as chronic pelvic pain [30], depression[69], stroke, Parkinson's disease [67]. Its immediate and long-lasting effects on an animal model of subacute pain related to chronic inflammation have also been demonstrated.[70] However, its use for the purpose of pain management in peri-operative periods still needs to be better studied. A single repetitive transcranial magnetic stimulation (rTMS) session was associated with reduction in opioid consumption in the postoperative period of gastric bypass[33]. Although tDCS did not show benefit in the postoperative period of lumbar spine surgeries [34], it has shown a reduction in opioid consumption in the postoperative period of total knee arthroplasty [35] and a single 20-minute session has already shown a reduction in the use of opioids in the postoperative period of endoscopic procedures, as well as demonstrating safety and minimal adverse effects [36]. The present study aims to evaluate the effect of tDCS applied in the preoperative period as a coadjuvant method for the control of postoperative pain of surgical correction of hallux valgus in patients with arthralgia of the 1st MPJ.

3. Justification

Considering the accumulation of evidence on the benefits of this technique, its rare adverse effects, its easy application in comparison to other neuromodulatory techniques, the prevalence of arthralgia in patients with hallux valgus, the social relevance of the impossibility of wearing shoes due to arthralgia and the possibility of inclusion of a new analgesia method for peri-operative periods, innovative studies that may provide advances in the therapeutic process of arthralgia of the 1st MPJ are justified. If the efficacy of tDCS in this period is confirmed, a great advance can be achieved in the

scenario of multimodal analgesia and reduction of suffering of the individuals submitted to surgical procedures. Interest in this pathology is based on the premise that this chronic pain condition constitutes a large part of the demand for specialized medical care in orthopedics.

4. Objective

4.1 Main objective

To evaluate the effect of tDCS compared to tDCS-sham in the perioperative pain control, rehabilitation and anxiety in patients with arthralgia of the 1st MPJ undergoing surgical treatment of hallux valgus.

4.2 Secondary objectives

- To evaluate the effect of tDCS compared to tDCS-sham in the treatment of post-operative pain of hallux valgus surgical correction in the following outcomes:

- 10 cm Numerical Pain Scale (NPS) pain levels in the first 8 postoperative days.

- To evaluate the peripheral sensitization of nociceptors through pattern algogenic stimuli induced by quantitative sensory test (QST).

- Function of the descendent modulator system to the heterotopic stimulus.

- Consumption of analgesics.

- Functional pain scale. (B-PCP:S)

- Levels of catastrophic thinking.

- Serum levels of BDNF and S100B.

- BDNF levels in the CSF after transcranial stimulation.

5. Materials and methods

5.1 Overall design: Clinical, randomized, double-blinded, parallel, placebo-sham-controlled trial.

5.2 Logistics: The recruitment, interventions and follow-up of the patients of the present research project will be carried out at the Hospital Independência de Porto Alegre, at the edge of the infirmary bed in patients hospitalized for surgical procedure. Laboratory tests will be performed at the Hospital de Clínicas, Porto Alegre. All participants will sign a free and informed consent form. The study will be registered in the Database -

Plataforma Brasil, clinicaltrials.gov and its assembly follows CONSORT (Consolidated Standards of Reporting Trials).

5.3 Sampling: Female patients submitted to surgical treatment of hallux valgus under a combined Chevron + Akin technique will be included.

5.3.1 Inclusion criteria: age above 18 years and younger than 70 years, female, indication of surgical treatment of hallux valgus for arthralgia of the 1st MPJ, able to understand and write the Portuguese language.

5.3.2 Exclusion criteria: patients with diabetic neuropathy, presence of contraindications to tDCS [58] (History of severe or frequent headache, chronic dermatological disorders, adverse reactions to previous treatment with tDCS, history of seizures, severe head injury with cranial anatomy, intracranial metal implants, or pacemaker), uncontroled psychiatric illness or uncooperative patients, history of neurological disease, oncology, ischemic heart disease, renal or hepatic impairment.

5.4 Sample size: The sample size was estimated considering as the primary outcome the cumulative mean of the worst daily pain scores in the VAS during the first 48 hours postoperatively. It is estimated that 28 patients will be required, which will be allocated randomly in 2 groups of 14 patients. Predicting possible losses and multiple outcomes, the sample size was increased for 40 patients (20 per group). The number of patients was defined to detect a reduction in pain scores, with tDCS, of at least 1.5 cm in VAS (0-10 cm) with a standard deviation of 0.9 cm. This difference between means has been considered clinically relevant in the context of treatment of acute postoperative pain in different scenarios [71, 72]. A chance of alpha error of 0.5 and beta error of 20% is accepted.

5.5 Randomization and blinding: Randomization will be done in blocks of four, dividing the patients into two groups: tDCS or tDCS-sham. The randomization table will be generated by randomization website (www.randomization.com). Randomization codes will be handled by an independent researcher who will be responsible for the application of the intervention and will be the only professional who will not be blinded. This professional will not participate in the selection, measurement or evaluation of outcomes. All patients and other professionals, including outcome assessors, will be blinded. Randomization codes will be placed in brown envelopes, sealed and initialed by the researcher responsible to avoid any possibility of violation. The envelopes will be

numbered externally to indicate the sequence previously defined in the randomization. Blinding will be maintained at all stages of the study.

5.6 Intervention

tDCS enforcement - The tDCS or sham stimulation (control group) will be applied to the skulls of the patients in 20 minute sessions performed the night before the surgical procedure and on the morning of the procedure day, totaling 2 sessions, as follows:

a) Active stimulation – it will be applied by a pair of sponge coated surface electrodes and soaked in saline solution. A constant current stimulator powered by battery will be used for this purpose. (tDCS device Soterix 1X1). Stimulation will be performed by placing the anodal stimulus electrode in the primary motor cortex (M1) – area corresponding to the C3 area (EEG 10/20 system) and the catodal electrode on the contralateral supraorbital area. The assembly will be the standardized in our laboratory, which follows that used by Fregni et al. [12, 73]. The active stimulation will be performed with 2 mA current.

b) Sham stimulation – In the sham stimulus group the electrodes will be placed in the same positions as those in the M1 anodal stimulation group, however, the stimulator will be programmed to shut down after 30 seconds of stimulation. Therefore patients will feel the sensation of initial stimulation, but will not receive current for the remaining period. This sham method has been shown to be efficient in blinding [74]

5.7 Outcomes

5.7.1 Primary outcomes

<u>Clinical parameters</u>:

A) Pain: - pain scores in NPS

- post operative analgesic medication consumption
- functional pain scale [75]

5.7.2 Secondary outcomes

<u>Clinical parameters</u>:

B) Pre operative anxiety: it will be measured with the refined version of the Trait and State Anxiety Scale (STAI), adapted to Brazilian Portuguese [76]. State anxiety

(transient state caused by a specific situation) will be assessed the night before the procedure (baseline period), and just before the surgical procedure.

C) Evaluation of the pain threshold and the descending pain modulator system

D) Demographic data and comorbidities

Laboratorial parameters:

E) BDNF and S100B levels: The collected sample of CSF will be aliquoted and stored at -70 ° C for further analysis. The whole blood sample will be collected and centrifuged, the serum will be aliquoted and stored at -70 ° C for further analysis. The samples will be stored by the Laboratory of Pain and Neuromodulation coordinated by Prof. Dr. Wolnei Caumo, collaborator of this project. BDNF and S100B will be measured by the ELISA technique according to commercial kit instructions.

5.8 Measurements of outcomes

Clinical parameters:

A) Pain – will be measured by:

- Numerical Pain Scale (NPS) 0-10 included in a standardized questionnaire whose zero corresponds to the absence of pain and 10, maximum pain. The assessment will be done in two daily scores: one score for the worst daily pain during walking and another score for the average daily pain at rest. During the tDCS sessions, a score will be assigned for the pre- and post-tDCS moments.

- Functional pain scale - the Brazilian Profile of Chronic Pain: Screen (B-PCP:S) will be used for rapid identification of the individual multidimensional pain experience. It consists of a pain severity ladder (four items, a possible scale of 0-30), an interference scale (six items, a possible scale of 0-36), and an emotional load scale (five items, possible scale of 0-25)[76]. The importance of these three dimensions (severity, interference, and emotional load) was recently highlighted by The Initiative on Methods, Measurement, and Pain Assessment in Clinical Trials. It will be applied in the basal period, and on the 8th postoperative day.

- Daily consumption of supplemental analgesics during the first seven postoperative days. Prescription in the preoperative period, immediate postoperative period and after hospital discharge will be standardized and daily dose, date and time of consumption will be recorded by standard questionnaire.

B) Preoperative anxiety will be measured with the refined version of the State and Trait Anxiety Index (STAI), adapted to Brazilian Portuguese [76]. State anxiety (transient state caused by a specific situation) will be assessed at baseline, and just before the surgical procedure.

C) Evaluation of pain threshold and pain descending modulatory systems

- Heat Pain Test or Quantitative Sensory Test (QST): A computerized version of the thermotest (Heat Pain Stimulator 1.1.10, Brazil developed by our group)[78] Will be used to determine the maximum tolerated temperature and the heat pain threshold on the volar side of the dominant forearm and the medial portion of the leg ipsilateral to the surgical site. The temperature starts at 32 ° C, the thermoswitch is heated at a rate of 1.0 ° C / sec to a maximum of 52 ° C when the temperature begins to decrease. The patient will push a button when she can no longer tolerate the increase in temperature. The pain threshold for heat is determined by the average of three evaluations. The QST Test will be applied the day before the surgical procedure (before the first intervention session), and at the end of the procedure day, after complete regression of sensory blockade induced by subarachnoid anesthesia. The application of the test in two different sites (forearm and leg) will allow the investigation and differentiation of central or peripheral sensitization of nociceptive receptors.

- CPM (conditioned pain modulation): it will be evaluated to access the descending modulatory system. For this purpose, a nociceptive stimulus is used, such as immersion of the non-dominant hand in the water at a temperature of zero up to 1,5°C concomitant with the thermal stimulus in the dominant forearm. The degree of inhibition that the heterotopic stimulus (hand in the water) causes on the threshold of thermal pain is measured in the dominant forearm. To do so, initially the average of three temperature measures in which the patient reports pain 6/10 following the standard of the QST test is determined. The contralateral hand is then immersed in the water at a temperature of zero to 1.5 ° C while the gradual thermal stimulus is applied to the dominant forearm until it reaches the temperature at which it was reported by the patient as 6/10 on the pain scale . The mean of three measurements with 15-minute rest intervals will be used as the measure of the conditioning pain score. The difference will be presented as a percentage of the mean score variation before and after immersion of the hand in the water. The CPM Test will be applied the day before the surgical procedure (before the first intervention

session), and at the end of the procedure day, after complete regression of sensory blockade induced by subarachnoid anesthesia.

Measurement of laboratory parameters outcomes

E) BDNF and S100B levels - Blood samples will be collected at three different times: the night before the procedure (before the first tDCS session), the day of surgery (just before the anesthetic procedure for the surgical procedure), and in the ambulatory return appointment (8th post-operative day). CSF collection will be performed during subarachnoid anesthesia in a single moment. The material will be prepared and conditioned in an appropriate environment immediately after collection.

5.9 Confounding factors:

- **Demographics and comorbidities**, as well as previous medication use will be assessed by standard questionnaire.

- The **depressive symptoms levels** will be measured by the Beck II Inventory [78]. Depressive symptoms will be measured at baseline.

- The **catastrophic thinking level** will be measured by the Catastrophic pain scale[79] in the basaline.

5.10 Anesthetic and surgical technique5.10.1 Preoperative medication – it will be prescribed medications and therapies previously in use by the patient, as well as analgesic medications if necessary (dipyrone EV 6/6h if pain, tramadol 50mg EV 6/6h if severe pain). No anxiolytic or analgesic medications will be prescribed in a fixed manner.

5.10.2 Anesthetic technique - Subarachnoid anesthesia for the surgical procedure will be performed with a needle for this purpose (Quincke 25G) and the medications administered in the subarachnoid space, after collection of cerebral spinal fluid (CSF), will be standardized: hyperbaric bupivacaine 12.5mg + morphine 80mcg. Immediately after subarachnoid anesthesia, patients will be lightly sedated with 50mcg of fentanyl and 1 to 5mg of midazolam depending on the individual patient need for comfort in the intraoperative period. Additional doses of propofol bolus or continuous propofol may be given in cases where the patient remains alert despite the sedatives administered.

5.10.3 Surgical technique – All patients will undergo the combined Chevron + Akin osteotomy technique after the establishment of subarachnoid anesthesia. The estimated duration of the procedure is one hour.

5.10.4 Postoperative medication - The prescription in the immediate postoperative period will be: dipyrone EV 1g 6/6h if pain, tramadol 50mg EV 6/6h if severe pain and morphine 3mg EV 3/3h if pain refractory to tramadol. After hospital discharge, the home oral prescription will be standardized: dipyrone 1g 6/6h if pain, tramadol 50mg 6/6h if severe pain.

5.11 Adverse effects – The occurrence of any adverse effects such as paresthesia, headache, dizziness, nausea, neck pain, burning, erythema or pain on the scalp, insomnia, abrupt mood changes and difficulty concentrating will be questioned at the end of each tDCS session.

5.12 Postoperative clinical follow-up – The patients will receive a medical visit once a day while hospitalized in order to check the amount of analgesics consumption and to evaluate the pain control by the NPS score. After hospital discharge (planned for the first postoperative day), patients will receive a daily phone call to answer pain scores and inform analgesic consumption until their ambulatory return appointment scheduled for the eighth postoperative day.

5.13 Risks – The possible discomforts related to the intervention's application are: small shocks, itching and redness in the region where the tDCS electrodes will be installed and, rarely, dizziness, headache and vertigo. Considering the practice of common spinal anesthesia in the care of patients submitted to surgical correction of hallux valgus, the following risks, although existing due to the presence of lumbar puncture for spinal anesthesia, were considered not exclusive to the research participant: post-lumbar puncture headache, lumbar puncture site discomfort, hypotension, infection, transient or permanent neurological sequelae.

5.14 Procedures sequence

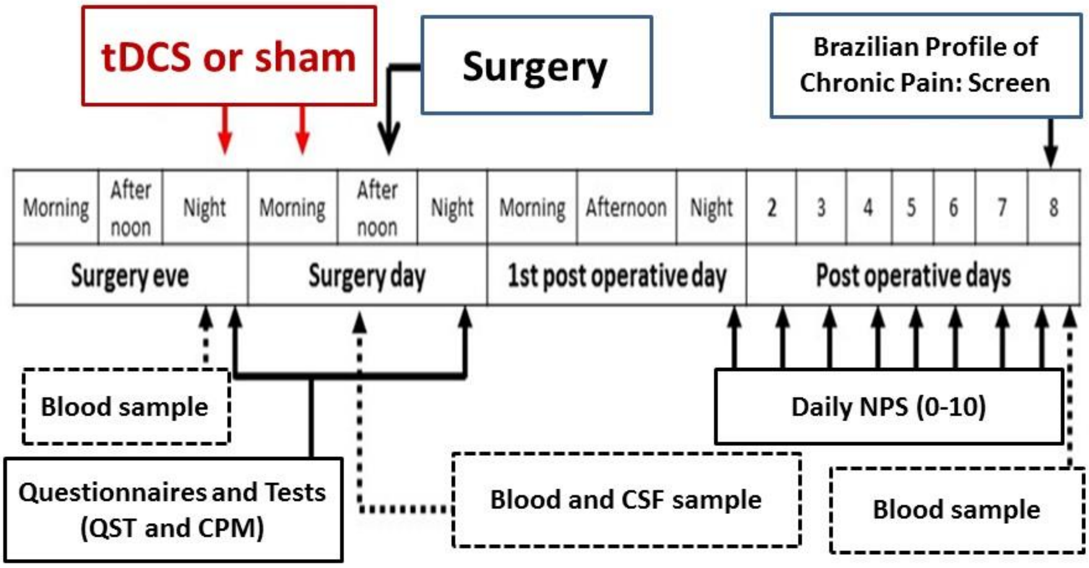

6. Data processing and statistical analysis

Descriptive analysis of data with mean, frequency or proportion shall be carried out. The difference between the groups for continuous variables will be evaluated by the t-test, and for categorical variables the qui-square test or the Fisher's exact test. After assessing the distribution of the outcome variables by the Skewness and Kurtosi tests, outcome variables that present a normal or approximately normal distribution will be compared between the 2 treatment groups by the Student t test. For outcome variables that do not present criteria for using parametric statistics, the effect between the treatment groups will be analyzed using the Wilcoxon-Mann-Whitney U test. ANOVA of repeted measures will be used to compare the measures taking into account the time (NPS) and the effect of the subject. If necessary, linear regression will be performed to adjust the confounding variables. The chance of error α acceptable for all analyzes will be 5%. The data will be analyzed in the program SPSS 20.0.

7. Ethical aspects

This protocol follows the conditions of resolution 466/12 of the National Health Council (NHC). After clarifying the purpose of the study, the patients will sign the informed consent form. This project will be submitted for consideration by the ethics committee of the Hospital de Clínicas of Porto Alegre.

8. Project feasibility

This project is within the research line of the Laboratory of Pain and Neuromodulation of Hospital de Clínicas of Porto Alegre. Therefore the techniques described are already known and are already being applied in different studies. The sample will consist of patients belonging to the Hospital Independência of Porto Alegre.

9. Quality control

The team will be trained to apply the evaluations. Weekly meetings will be held to discuss possible doubts arising during the implementation of the protocol. The instructions for all the steps and methods of measurement will be included in an instruction manual.

10. Detailed budget

Quantity	Description	Unity Value	Total Value
6	Sample Storage (monthly)	R$ 60,00	R$ 360,00
2	Tubes for blood sample – box with 100	R$ 35,00	R$ 70,00
50	Bottles for CSF samples	R$ 1,05	R$ 52,50
48	Appointments (40 + 20% = 48)	R$ 7,55	R$ 362,40
50	Envelopes - Randomization and questionnaires	R$ 0,50	R$ 25,00
500	Press - Copies of questionnaires	R$ 0,20	R$ 100,00
2	Yellow or colorless tip of 0-200ul pack with 1000	R$ 14,80	R$ 29,60
10	Tip p-1000 cap. 200-1000ul sterile racks of 96 units	R$ 0,13	R$1,30
10	Saline 250ml 0,9% 0,15meq/ml	R$ 0,79	R$ 7,90
1	Tube Eppendorf 1,5ml pack with 1000 units - Sterile	R$ 40,00	R$ 40,00
2	Tube tp Eppendorf 0,5ml pack with 500 units	R$ 30,00	R$ 60,00
1	human BDNF ELISA kit	R$ 3.100,00	R$ 3.100,00
**TOTAL**			**R$ 4208,70**

11. Schedule

	2014											
	Jan	Feb	Mar	Apr	Ma	Jun	Jul	Aug	Sept	Oct	Nov	Dec
Project elaboration								■	■			
Approval by the Ethics and Research Committee									■	■	■	
Team training and logistics									■	■	■	
Data collect											■	■
2015												
Data collect	■	■	■	■	■	■						
Database					■	■						
Statistical treatment							■	■				
Writing									■	■		

12. REFERENCES

1. PA, V., *Anatomia, patologia del pie*. 3rd ed1984.
2. Nery CAS, H.S., Xavier R, Pardini Jr AG, Barros Filho TEP, *Ortopedia e traumatologia – Princípios e prática*. 3rd ed2003.
3. Cavanagh, P.R., M.M. Rodgers, and A. Iiboshi, *Pressure distribution under symptom-free feet during barefoot standing.* Foot Ankle, 1987. **7**(5): p. 262-76.
4. Coughlin, M.J. and C.P. Jones, *Hallux valgus: demographics, etiology, and radiographic assessment.* Foot Ankle Int, 2007. **28**(7): p. 759-77.
5. Melzack, R., *Pain--an overview.* Acta Anaesthesiol Scand, 1999. **43**(9): p. 880-4.
6. Casey, K.L., *Forebrain mechanisms of nociception and pain: analysis through imaging.* Proc Natl Acad Sci U S A, 1999. **96**(14): p. 7668-74.
7. Tracey, I. and P.W. Mantyh, *The cerebral signature for pain perception and its modulation.* Neuron, 2007. **55**(3): p. 377-91.
8. Ploner, M. and A. Schnitzler, *[Cortical representation of pain].* Nervenarzt, 2004. **75**(10): p. 962-9.
9. Talbot, J.D., et al., *Multiple representations of pain in human cerebral cortex.* Science, 1991. **251**(4999): p. 1355-8.
10. Boros, K., et al., *Premotor transcranial direct current stimulation (tDCS) affects primary motor excitability in humans.* Eur J Neurosci, 2008. **27**(5): p. 1292-300.
11. Floel, A., et al., *Noninvasive brain stimulation improves language learning.* J Cogn Neurosci, 2008. **20**(8): p. 1415-22.
12. Fregni, F., et al., *A sham-controlled, phase II trial of transcranial direct current stimulation for the treatment of central pain in traumatic spinal cord injury.* Pain, 2006. **122**(1-2): p. 197-209.
13. Fregni, F., et al., *A randomized, sham-controlled, proof of principle study of transcranial direct current stimulation for the treatment of pain in fibromyalgia.* Arthritis Rheum, 2006. **54**(12): p. 3988-98.
14. Hummel, F. and L.G. Cohen, *Improvement of motor function with noninvasive cortical stimulation in a patient with chronic stroke.* Neurorehabil Neural Repair, 2005. **19**(1): p. 14-9.

15. Wagner, T., et al., *Transcranial direct current stimulation: a computer-based human model study.* Neuroimage, 2007. **35**(3): p. 1113-24.
16. Nitsche, M.A., et al., *Pharmacological modulation of cortical excitability shifts induced by transcranial direct current stimulation in humans.* J Physiol, 2003. **553**(Pt 1): p. 293-301.
17. Nitsche, M.A., et al., *Modulation of cortical excitability by weak direct current stimulation--technical, safety and functional aspects.* Suppl Clin Neurophysiol, 2003. **56**: p. 255-76.
18. Nitsche, M.A., et al., *Transcranial direct current stimulation: State of the art 2008.* Brain Stimul, 2008. **1**(3): p. 206-23.
19. Auvichayapat, P. and N. Auvichayapat, *Basic knowledge of transcranial direct current stimulation.* J Med Assoc Thai, 2011. **94**(4): p. 518-27.
20. Raghavan, S., S. Eldabe, and R. Strachan, *Novel trends in pain management – Neuromodulation.* Current Anaesthesia & Critical Care, 2008. **19**: p. 344-348.
21. Nitsche, M.A. and W. Paulus, *Excitability changes induced in the human motor cortex by weak transcranial direct current stimulation.* J Physiol, 2000. **527 Pt 3**: p. 633-9.
22. Utz, K.S., et al., *Electrified minds: transcranial direct current stimulation (tDCS) and galvanic vestibular stimulation (GVS) as methods of non-invasive brain stimulation in neuropsychology--a review of current data and future implications.* Neuropsychologia, 2010. **48**(10): p. 2789-810.
23. Liebetanz, D., et al., *After-effects of transcranial direct current stimulation (tDCS) on cortical spreading depression.* Neurosci Lett, 2006. **398**(1-2): p. 85-90.
24. Siebner, H.R., et al., *Preconditioning of low-frequency repetitive transcranial magnetic stimulation with transcranial direct current stimulation: evidence for homeostatic plasticity in the human motor cortex.* J Neurosci, 2004. **24**(13): p. 3379-85.
25. Nitsche, M.A. and W. Paulus, *Sustained excitability elevations induced by transcranial DC motor cortex stimulation in humans.* Neurology, 2001. **57**(10): p. 1899-901.
26. Garcia-Larrea, L., et al., *Electrical stimulation of motor cortex for pain control: a combined PET-scan and electrophysiological study.* Pain, 1999. **83**(2): p. 259-73.
27. Strafella, A.P., Y. Vanderwerf, and A.F. Sadikot, *Transcranial magnetic stimulation of the human motor cortex influences the neuronal activity of subthalamic nucleus.* Eur J Neurosci, 2004. **20**(8): p. 2245-9.
28. Arnold, L.M., *Biology and therapy of fibromyalgia. New therapies in fibromyalgia.* Arthritis Res Ther, 2006. **8**(4): p. 212.
29. Lefaucheur, J.P., *Transcranial magnetic stimulation in the management of pain.* Suppl Clin Neurophysiol, 2004. **57**: p. 737-48.
30. Fenton, B.W., et al., *A preliminary study of transcranial direct current stimulation for the treatment of refractory chronic pelvic pain.* Brain Stimul, 2009. **2**(2): p. 103-7.
31. Reidler, J.S., et al., *Effects of motor cortex modulation and descending inhibitory systems on pain thresholds in healthy subjects.* J Pain, 2012. **13**(5): p. 450-8.
32. Spezia Adachi, L.N., et al., *Reversal of chronic stress-induced pain by transcranial direct current stimulation (tDCS) in an animal model.* Brain Res, 2012. **1489**: p. 17-26.
33. Borckardt, J.J., et al., *Postoperative left prefrontal repetitive transcranial magnetic stimulation reduces patient-controlled analgesia use.* Anesthesiology, 2006. **105**(3): p. 557-62.
34. Dubois, P.E., et al., *Postoperative analgesic effect of transcranial direct current stimulation in lumbar spine surgery: a randomized control trial.* Clin J Pain, 2013. **29**(8): p. 696-701.
35. Borckardt, J.J., et al., *Transcranial direct current stimulation (tDCS) reduces postsurgical opioid consumption in total knee arthroplasty (TKA).* Clin J Pain, 2013. **29**(11): p. 925-8.

36. Borckardt, J.J., et al., *Feasibility, safety, and effectiveness of transcranial direct current stimulation for decreasing post-ERCP pain: a randomized, sham-controlled, pilot study.* Gastrointest Endosc, 2011. **73**(6): p. 1158-64.
37. Menz, H.B., et al., *Impact of hallux valgus severity on general and foot-specific health-related quality of life.* Arthritis Care Res (Hoboken), 2011. **63**(3): p. 396-404.
38. J, R., *De la deviation en dehors du gros orl (hálux valgus) et son traitement chirurgical.* Trans Int Med Cong, 1881.
39. Mann, R.A. and K.C. Donatto, *The chevron osteotomy: a clinical and radiographic analysis.* Foot Ankle Int, 1997. **18**(5): p. 255-61.
40. Schneider, W., et al., *Chevron osteotomy in hallux valgus. Ten-year results of 112 cases.* J Bone Joint Surg Br, 2004. **86**(7): p. 1016-20.
41. Tollison, M.E. and D.E. Baxter, *Combination chevron plus Akin osteotomy for hallux valgus: should age be a limiting factor?* Foot Ankle Int, 1997. **18**(8): p. 477-81.
42. RAL, C.M.C.P.F.G.F.R.S., *Correção do hálux valgo leve ou moderado utilizando as osteotomias de Akin e chevron combinadas.* Rev. Bras. Med., 2010(67): p. 11-16.
43. Trnka, H.J., et al., *The chevron osteotomy for correction of hallux valgus. Comparison of findings after two and five years of follow-up.* J Bone Joint Surg Am, 2000. **82-A**(10): p. 1373-8.
44. Rocha, A.P., et al., *Pain: current aspects on peripheral and central sensitization.* Rev Bras Anestesiol, 2007. **57**(1): p. 94-105.
45. Manji, H.K. and R.S. Duman, *Impairments of neuroplasticity and cellular resilience in severe mood disorders: implications for the development of novel therapeutics.* Psychopharmacol Bull, 2001. **35**(2): p. 5-49.
46. Marenholz, I., C.W. Heizmann, and G. Fritz, *S100 proteins in mouse and man: from evolution to function and pathology (including an update of the nomenclature).* Biochem Biophys Res Commun, 2004. **322**(4): p. 1111-22.
47. Tanga, F.Y., et al., *Role of astrocytic S100beta in behavioral hypersensitivity in rodent models of neuropathic pain.* Neuroscience, 2006. **140**(3): p. 1003-10.
48. Arts, B., et al., *S100 and impact of ECT on depression and cognition.* J ECT, 2006. **22**(3): p. 206-12.
49. Grabe, H.J., et al., *Neurotrophic factor S100 beta in major depression.* Neuropsychobiology, 2001. **44**(2): p. 88-90.
50. Zhou, Z., et al., *Brain-specific phosphorylation of MeCP2 regulates activity-dependent Bdnf transcription, dendritic growth, and spine maturation.* Neuron, 2006. **52**(2): p. 255-69.
51. Schinder, A.F. and M. Poo, *The neurotrophin hypothesis for synaptic plasticity.* Trends Neurosci, 2000. **23**(12): p. 639-45.
52. Kossel, A.H., et al., *A caged Ab reveals an immediate/instructive effect of BDNF during hippocampal synaptic potentiation.* Proc Natl Acad Sci U S A, 2001. **98**(25): p. 14702-7.
53. Grider, M.H., et al., *In situ expression of brain-derived neurotrophic factor or neurotrophin-3 promotes sprouting of cortical serotonergic axons following a neurotoxic lesion.* J Neurosci Res, 2005. **82**(3): p. 404-12.
54. Jovanovic, J.N., et al., *Synapsins as mediators of BDNF-enhanced neurotransmitter release.* Nat Neurosci, 2000. **3**(4): p. 323-9.
55. Wingo, W.J. and J. Awapara, *Decarboxylation of L-glutamic acid by brain.* J Biol Chem, 1950. **187**(1): p. 267-71.
56. Fenalti, G. and M.J. Rowley, *GAD65 as a prototypic autoantigen.* J Autoimmun, 2008. **31**(3): p. 228-32.
57. Shirayama, Y., et al., *Brain-derived neurotrophic factor produces antidepressant effects in behavioral models of depression.* J Neurosci, 2002. **22**(8): p. 3251-61.
58. DaSilva, A.F., et al., *Electrode positioning and montage in transcranial direct current stimulation.* J Vis Exp, 2011(51).

59. Stagg, C.J., et al., *Polarity-sensitive modulation of cortical neurotransmitters by transcranial stimulation.* J Neurosci, 2009. **29**(16): p. 5202-6.
60. Antal, A., W. Paulus, and M.A. Nitsche, *Electrical stimulation and visual network plasticity.* Restor Neurol Neurosci, 2011. **29**(6): p. 365-74.
61. Stagg, C.J. and M.A. Nitsche, *Physiological basis of transcranial direct current stimulation.* Neuroscientist, 2011. **17**(1): p. 37-53.
62. Nitsche, M.A., et al., *GABAergic modulation of DC stimulation-induced motor cortex excitability shifts in humans.* Eur J Neurosci, 2004. **19**(10): p. 2720-6.
63. Nitsche, M.A., et al., *Dopaminergic modulation of long-lasting direct current-induced cortical excitability changes in the human motor cortex.* Eur J Neurosci, 2006. **23**(6): p. 1651-7.
64. Nitsche, M.A., et al., *D1-receptor impact on neuroplasticity in humans.* J Neurosci, 2009. **29**(8): p. 2648-53.
65. Monte-Silva, K., et al., *Dosage-dependent non-linear effect of L-dopa on human motor cortex plasticity.* J Physiol, 2010. **588**(Pt 18): p. 3415-24.
66. Nitsche, M.A., et al., *Serotonin affects transcranial direct current-induced neuroplasticity in humans.* Biol Psychiatry, 2009. **66**(5): p. 503-8.
67. Medeiros, L.F., et al., *Neurobiological effects of transcranial direct current stimulation: a review.* Front Psychiatry, 2012. **3**: p. 110.
68. Thirugnanasambandam, N., et al., *Isometric contraction interferes with transcranial direct current stimulation (tDCS) induced plasticity: evidence of state-dependent neuromodulation in human motor cortex.* Restor Neurol Neurosci, 2011. **29**(5): p. 311-20.
69. Nitsche, M.A., et al., *Treatment of depression with transcranial direct current stimulation (tDCS): a review.* Exp Neurol, 2009. **219**(1): p. 14-9.
70. Laste, G., et al., *After-effects of consecutive sessions of transcranial direct current stimulation (tDCS) in a rat model of chronic inflammation.* Exp Brain Res, 2012. **221**(1): p. 75-83.
71. KAS, C., *Another decision to evaluate: choice of standardized mean difference effect size estimator*, in *Sixth International Cochrane Colloquium*1998: Baltimore, MD.
72. Brattwall, M., I. Turan, and J. Jakobsson, *Pain management after elective hallux valgus surgery: a prospective randomized double-blind study comparing etoricoxib and tramadol.* Anesth Analg, 2010. **111**(2): p. 544-9.
73. Valle, A., et al., *Efficacy of anodal transcranial direct current stimulation (tDCS) for the treatment of fibromyalgia: results of a randomized, sham-controlled longitudinal clinical trial.* J Pain Manag, 2009. **2**(3): p. 353-361.
74. Gandiga, P.C., F.C. Hummel, and L.G. Cohen, *Transcranial DC stimulation (tDCS): a tool for double-blind sham-controlled clinical studies in brain stimulation.* Clin Neurophysiol, 2006. **117**(4): p. 845-50.
75. Caumo, W., et al., *Cross-cultural adaptation and validation of the profile of chronic pain: screen for a Brazilian population.* Pain Med, 2013. **14**(1): p. 52-61.
76. Kaipper, M.B., et al., *Evaluation of the structure of Brazilian State-Trait Anxiety Inventory using a Rasch psychometric approach.* J Psychosom Res, 2010. **68**(3): p. 223-33.
77. Schestatsky, P., et al., *Transient decrease of sensory perception after thermoalgesic stimuli for quantitative sensory testing.* Muscle Nerve, 2007. **36**(4): p. 466-70.
78. Andrade, L., et al., *Psychometric properties of the Portuguese version of the State-Trait Anxiety Inventory applied to college students: factor analysis and relation to the Beck Depression Inventory.* Braz J Med Biol Res, 2001. **34**(3): p. 367-74.
79. Sehn, F., et al., *Cross-cultural adaptation and validation of the Brazilian Portuguese version of the pain catastrophizing scale.* Pain Med, 2012. **13**(11): p. 1425-35.

ATTACHMENT 1

FREE AND INFORMED CONSENT TERM

INSTITUTION: Hospital Independência and Hospital de Clínicas de Porto Alegre

Name of the participant:____

You are being invited to participate in the study "Effect of transcranial direct current stimulation (tDCS) in the preoperative period of surgical treatment of hallux valgus" in order to evaluate the effect of pain control by the application of low intensity stimuli applied to the head.

1. OBJECTIVES OF THE STUDY – You will be submitted, except in cases of refusal, to standard (spinal) anesthesia for surgical correction of hallux valgus regardless of participation in this study. This study aims to evaluate the effect of the application of a complementary method to standard anesthesia for the control of postoperative pain and preoperative anxiety: tDCS (which is a device applied to the head that emits an electric current).

2. EXPLANATION OF PROCEDURES - The aim of this study is to evaluate the effect of tDCS through questionnaires, CSF and blood tests, and other specific tests. You will have to answer some questions, fill out questionnaires and allow a blood sample (5mL) even before the tDCS application. After completing the questionnaires, specific tests will be done to evaluate how much you feel in pain or discomfort when applying a device that gradually heats up in your arm. The following day, during spinal anesthesia (which is applied independently of participation in the study), a further collection of minimal amount of blood (5mL) and CSF (1mL) will be done to evaluate the level of pain markers contained in these samples. CSF is the liquid that is in contact with the spinal cord and the brain and in all spinal anesthesia its flow through the needle inserted in the back is used to certify that the needle is inserted in the ideal place of application of the anesthetic medicine. With your participation in this study, 1mL of this liquid will be collected (quantity quickly produced and returned by the patient's body, without additional risk to those inherent in spinal anesthesia). After hospital discharge, you will fill out a standard questionnaire once a day to record how much you feel in pain. On the day of your outpatient return (8th day after surgery) you will bring the completed questionnaires to the appointment and the last blood collection will be done (5mL). Your participation is voluntary. If you agree, the questionnaires will be applied and we will start the study.

3. TREATMENTS - In this study, patients will be drawn in two groups. One group will receive a pain relief treatment that involves applying two wet electrodes through their head that will pass a weak electric current that can cause mild itching, tingling, and local redness in a 20-minute session. The other treatment group will be the placebo group, which will receive a treatment in which the equipment will not emit stimulation. Treatment sessions will be performed according to the protocol of this study, for a total of two sessions (one the night before surgery and one the morning of the day of surgery). The current is produced by an apparatus (figure below) produced for this purpose. Neither you nor the evaluator who will apply the questionnaires will know what treatment you received.

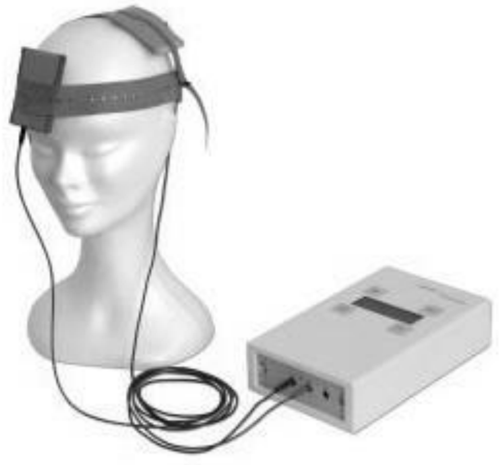

4. POSSIBLE RISKS E DISCONFORT - The possible discomforts related to the use of tDCS are: small shocks, itching and redness in the region where the tDCS electrodes will be installed and, rarely, dizziness, headache and vertigo. Some discomfort may also be felt during blood collection. There are also risks inherent to the practice of spinal anesthesia, which is applied even in patients not participating in the research: post-lumbar puncture headache, discomfort at the lumbar puncture site, pressure drop, infection, transient or permanent neurological sequelae.

5. POSSIBLE BENEFITS OF THE STUDY – Participation in the study has as a benefit to the participant a possible better control of pain and anxiety, and will contribute to increase the knowledge about the area studied. The results of this study may provide important information about the role of transcranial direct current stimulation in the control of postoperative pain. We may initiate the alternate use of a technique that may benefit other patients with pain pictures similar to yours.

6. REIMBURSEMENT OF EXPENSES - You will have no expenses with your participation in researching and performing the procedures involved. No type of payment is also foreseen for participation in the study.

7. RIGHT OF WITHDRAWAL - You may withdraw from attending at any time. Your decisions not to participate or leave the survey after it has begun will not affect the continuation of any care within the institution.

8. STUDY EXCLUSION - The investigator in charge can exclude you from the study, without your consent, when he deems it is necessary, for the best referral of your case or if you do not comply with the established schedule.

9. PRIVACY - All information obtained from this study may be published for scientific purposes, preserving the identification data.

10. CONTACT OF RESEARCHERS - If you have any questions, you may contact the researchers through the following telephone numbers: Dr. Wolnei Caumo (2nd floor of the HCPA Pain & Neuromodulation Laboratory - Room 2201E - 3359-8083) or with the Ethics and Research Committee of Hospital de Clínicas - telephone 3359-8304 - from 8am to 5pm.

11. CONSENT – This term of Free and Informed Consent will be provided one copy for you and one copy will be stored by the researcher, the two copies being signed all pages by both. I declare that I have read - or have been read - the above information before signing this form. I was given ample opportunity to ask questions, fully clarifying my doubts. By this instrument, I voluntarily take part of the present study.

Name of participant or legal substitute: ____

Signature of participant ou legal substitute: ____

Substitute's degree of kinship: ____

Name of researcher: ____

Signature of researcher: ____

Porto Alegre, ______of ________________of 20____.
