# Supplementary material for: Preoperative transcranial direct current stimulation: Exploration of a novel strategy to enhance neuroplasticity before surgery to control postoperative pain. A randomized sham-controlled study
Source: PLoS One. 2017 Nov 30;12(11):e0187013. doi: 10.1371/journal.pone.0187013 (PMC5708693; doi:10.1371/journal.pone.0187013)
Supplement: S4 File — (PDF) [file pone.0187013.s004.pdf]

UNIVERSIDADE FEDERAL DO RIO GRANDE DO SUL

FACULDADE DE MEDICINA

PROGRAMA DE PÓS-GRADUAÇÃO EM MEDICINA: CIÊNCIAS MÉDICAS

PROJETO

EFEITO DA ESTIMULAÇÃO TRANSCRANIANA POR CORRENTE CONTINUA (ETCC) NO PERÍODO PRÉ-OPERATÓRIO DE TRATAMENTO CIRÚRGICO DE HÁLUX VALGO

HUGO DANIEL WELTER RIBEIRO

Médico graduado pela Universidade Federal do Paraná – UFPR. Anestesiologista pelo Centro de Ensino em Anestesiologia e Medicina Perioperatória do Hospital de Clínicas de Porto Alegre

ORIENTADOR

Prof. Wolnei Caumo, M.D., PhD.

Porto Alegre - 2014

LISTA DE FIGURAS

Figura 1 - Estratégia de busca de referências bibliográficas sobre as bases que fundamentam os objetivos deste estudo 09

Figura 2. Ilustração da técnica de Chevron..11

Figura 3. Ilustração da técnica de Akin...11

LISTA DE ABREVIATURAS

1ªAMF = primeira articulação metatarsofalangeana

AMP = adenosina monofosfato

AOFAS = American Orthopaedic Foot and Ankle Society

BDNF = *brain derived neurotrophic factor* (fator neurotrófico derivado do cérebro)

B-PCP:S = *Brazilian Profile of Chronic Pain: Screen*

CNS = Conselho Nacional de Saúde

CONSORT = *Consolidated Standards of Reporting Trials*

CPM = *conditioned pain modulation* (modulação condicionada da dor)

EAV = escala análogo visual

EMTr = estimulação magnética transcraniana repetitiva

ETCC = estimulação transcraniana por corrente contínua

GABA – ácido gama-aminobutírico

GAD = ácido glutâmico descarboxilase

HCPA = Hospital de Clínicas de Porto Alegre

LTP = *long-term potention* (potenciação de longa duração)

LTD = *long-term depression* (depressão de longa duração)

NGF = Fator de Crescimento Neural

NMDA = N-metil D-Aspartato

PLP = piridoxa-5'fosfato

QST = *quantitative sensory test*

STAI = escala de ansiedade traço e estado

tDCS = *transcranial Direct Current Stimulation*

RESUMO

Introdução: As deformidades do antepé são importantes causas de dor e desconforto para os pés e acometem cerca de 80% da população geral, com predominância na população feminina. Entre as deformidades comuns do antepé de adolescentes e adultos encontra-se o hálux valgo. Para atingir a cura desta deformidade, faz-se necessário o tratamento cirúrgico. A estimulação transcraniana de corrente contínua (ETCC) é uma técnica não invasiva, que visa à modulação do sistema nervoso central para controle da dor, podendo vir a se tornar uma opção terapêutica da dor pós-operatória. Estudos experimentais demonstraram que a ETCC reverteu a hiperalgesia induzida pelo estresse crônico e pela dor inflamatória articular, reduziu o consumo de opióides no período pós-operatório de artroplastia total de joelho e uma única sessão de 20 minutos já demonstrou redução no consumo de opióides no período pós-operatório de procedimentos endoscópicos, além de demonstrar segurança e mínimos efeitos adversos. No entanto, ainda não foi explorado o efeito da ETCC na hiperalgesia pré-operatória de pacientes sensibilizados, na ansiedade e na reabilitação pós-operatória de pacientes submetidas à cirurgia para correção de halux valgus. Neste estudo optou-se pelo uso da ETCC para estimular o córtex cerebral por ter eficácia demonstrada em síndromes dolorosas, por ser uma técnica não invasiva, apresentar baixo custo, ser de fácil aplicação em comparação com outras técnicas de neuroestimulação e sobretudo, pelo seu potencial de contra-regular as alterações neuroplásticas mal-adaptativas associadas à dor crônica. **Objetivo:** Avaliar o efeito da ETCC comparada ao ETCC-sham no controle da dor (escores na escala análogo visual de dor, limiar de dor e sistema modulador descendente da dor), ansiedade peri-operatória, consumo de analgésicos no pós-operatório e na reabilitação de pacientes com artralgia da articulação metatarsofalangeana (1ªAMF) submetidas a tratamento cirúrgico de hálux valgus. **Método:** Ensaio clínico, randomizado, cego, em paralelo, controlado com placebo-sham que inclui 40 pacientes do sexo feminino, entre 18 e 70 anos, candidatas a tratamento cirúrgico de hálux valgus sob técnica combinada de Chevron + Akin por artralgia da 1ªAMF. As pacientes serão randomizadas e divididas em dois grupos que receberá duas sessões de 20 minutos de ETCC ou ETCC-sham no período pré-operatório. **Resultados esperados:** Esse estudo irá avaliar o efeito da ETCC como forma de tratamento da dor pós-operatória de pacientes com hálux valgo e da ansiedade peri-operatória. Caso seja comprovada a sua eficácia, essa técnica poderá se tornar uma alternativa de baixo custo, fácil acesso, segura e eficaz para tratamento dessas pacientes.

ABSTRACT

Introduction: Forefoot deformities are important cause of feet pain and discomfort and affect around 80% of general population, predominantly in female population. Among teenagers and adults´ common forefoot deformities is hallux valgus. To achieve the deformity heal, surgical treatment is needed. The transcranial direct current stimulation (tDCS) is a non invasive technic, that aims for the central nervous system modulation for pain control, which can become a therapeutic option for postoperative pain. Experimental studies have shown that tDCS has reverted the hyperalgesia induced by chronic stress and articular inflammatory pain, has reduced total knee arthroplasty postoperative opioid consumption and a single 20 minutes session has shown reduction in endoscopic procedures opioid consumption, safety and minimal adverse effects. However, the tDCS effect in preoperative hyperalgesia has not been explored yet in sensitized patients, neither in anxiety and postoperative rehabilitation of patients submitted to hallux valgus surgical correction. In this study, it was chosen to use tDCS to stimulate cerebral cortex due to its efficacy in painful syndromes, for being a non invasive, low cost and easy-to-apply technic in comparison to other neurostimulation technics and especially, for its potential to counter-regulate the mal adaptive neuroplastic alterations associated to chronic pain. **Objective:** to evaluate the effect of tDCS compared to tDCS-sham in the pain control (visual analog scale score, pain threshold and the descendent modulator system), perioperative anxiety, postoperative analgesic drug consumption and in the rehabilitation of patients with arthralgia of the first metatarsophalangeal articulation submitted to hallux valgus surgical correction. **Method:** it is a randomized, blinded, placebo-sham controlled clinical trial which includes 40 female patients, between 18 and 70 years old, candidates to hallux valgus surgical treatment by combined Chevron + Akin osteotomy due to arthralgia of the first metatarsophalangeal articulation. The patients will be randomized and divided into two groups that will be treated with two tDCS or tDCS-sham sessions of 20 minutes each in preoperative period. **Expected results:** This study will evaluate the effect of tDCS as a treatment option to postoperative pain and perioperative anxiety of patients submitted to hallux valgus surgical correction. In case of proven efficacy, this technic can become a low cost, easy access, safe and effective treatment option for these patients.

1. Introdução

As deformidades do antepé são importantes causas de dor e desconforto para os pés. Cerca de 80% da população apresenta, durante a vida, alguma forma de dor ou calosidade nos pés[1, 2] e a incidência é nitidamente maior na população feminina, sugerindo uma possível relação com o tipo de calçado feminino[1]. Quando existe falha no equilíbrio anatômico e funcional que se estabelece entre as diversas estruturas estáticas e dinâmicas do pé, ocorrem as metatarsalgias, cuja etiologia está relacionada com as alterações biomecânicas do pé em aproximadamente 92% dos casos[1, 2].

Durante a marcha, a carga sobre o pé se concentra principalmente nos três ossos metatarsais mediais. Em cima disso, o estudo realizado por Cavanagh e col.[3] utilizou métodos eletrônicos computadorizados, o qual de fato confirmou que as distribuições de carga em condições estáticas tem nítido predomínio nos metatarsos centrais, especialmente no 2º e 3º raios [2, 3].

Entre as deformidades comuns do antepé de adolescentes e adultos encontra-se o hálux valgo. Essa anormalidade pode causar dor sobre a eminência medial da cabeça do primeiro metatarso, geralmente relacionada ao uso de calçados constritivos, os quais induzem a formação de bursite pelo excesso de atrito com a eminência. Nesse tipo de patologia, a deformidade inicialmente pode ser flexível, tornando-se progressivamente mais rígida e dolorosa com o passar do tempo. Alguns portadores da deformidade podem adequar seus calçados para um formato mais favorável, reduzindo a sintomatologia e assim, tornando possível a abordagem conservadora do hálux valgo. Para esse fim, os calçados com uma câmara anterior larga e alta podem ser os mais eficazes. No mercado já existem inúmeros dispositivos com o intuito de proteger o joanete, porém nenhum deles é capaz de corrigir a deformidade de maneira definitiva. Dessa forma, para atingir a cura da deformidade, faz-se necessário o tratamento cirúrgico.

Um dos principais componentes de uma deformidade do hálux valgo é o tamanho da eminência medial, frequentemente relatada como o foco da dor e da intolerância ao uso de calçados. Sugere-se que a eminência medial não seja um novo crescimento, mas sim uma porção do metatarso que se torna exposta com o desvio lateral da falange proximal do pé acometido[4]. Casos de deformidades mais graves do hálux valgo podem resultar em dor e deformidades adicionais nos raios laterais àquele acometido. Assim, pode haver dor e calosidade plantar sobre as cabeças dos 2º e 3º metatarsos, as quais são

geradas pela perda funcional da primeira articulação metatarsofalangeana[4]. Após passar por longo período de sobrecarga, o complexo da placa plantar sobrejacente às cabeças dos metatarsos laterais pode entrar em falência, levando ao desequilíbrio entre às forças flexoras e extensoras dos dedos e à consequente formação de dedos em garra.

A artralgia da primeira articulação metatarsofalangeana (1ªAMF) se caracteriza por determinar grande sofrimento e cursa com expressiva carga emocional. Considerando que a dor crônica é definida como condição associada à neuroplasticidade mal adaptativa e não uma simples reação a determinados tipos de estímulos, tratamo-la como uma experiência complexa, um fenômeno multidimensional que inclui componentes cognitivos, sensório-discriminativos, emocionais e motivacionais, mediado por mecanismos diferentes e processado em uma extensa rede cortical [5, 6]. Os circuitos envolvidos no processamento da dor em níveis cortical e subcortical, assim como os mecanismos celulares e moleculares subjacentes a este processamento tiveram substancial avanço nas últimas décadas. Esta evolução do conhecimento dos mecanismos neurobiológicos, conjuminada ao conceito de que dor crônica é um processo que envolve neuroplasticidade mal-adaptativa, permitiu que, o cérebro em conjunto com a medula espinhal, fosse considerado sistema ativo que filtra, seleciona e modula as aferências nociceptivas. Além disso, com o avanço na tecnologia dos exames de imagem, foi possível identificar regiões constantemente ativadas no processo nociceptivo agudo, como córtex somatossensorial primário e secundário, córtex insular, córtex pré-frontal, cingulado anterior e tálamo [7-9].

Com base nesses conhecimentos, opções terapêuticas que visam à modulação do sistema nervoso central para controle da dor passaram a ser mais frequentemente estudadas. Uma das técnicas que tem sido estudada para o tratamento da dor crônica é a estimulação transcraniana de corrente contínua (ETCC) [10-17]. Esta técnica utiliza corrente contínua de baixa intensidade (1-2 mA) [18] direcionada ao escalpo via eletrodos cátodo e ânodo, capaz de produzir alterações na excitabilidade cortical, com longevidade dos efeitos dependente da duração da estimulação. Ela produz alterações tanto em áreas próximas aos eletrodos, como em áreas distantes que têm conexão com a área primariamente estimulada. [19] Seus efeitos são dependentes da polaridade: a estimulação anódica induz excitabilidade cortical e a estimulação catódica a reduz. Os efeitos são explicados pela modulação do potencial da membrana neuronal vinculado à área estimulada [20] e a redução da excitabilidade neuronal ocorre pela redução da

atividade espontânea celular [21]. Esse efeito perdura mesmo após o término da estimulação. A duração das alterações depende do tempo e da intensidade de estimulação [22] e estima-se que seja aditivo à repetição dos cursos de estimulação. O efeito duradouro da ETCC deve-se ao aumento do input pré-sináptico e *up-regulation* do tônus sináptico mediado pelo receptor NMDA e dependente da síntese de proteína, modificações na concentração de AMP cíclico intracelular e do influxo da corrente de cálcio intracelular. Estes processos fazem parte dos fenômenos de *long-term potention* (LTP) e *long-term depression* (LTD) [19, 21-24]. A LTP corresponde a um processo de facilitação do sistema nervoso enquanto a depressão de longa duração LTD enfraquece a transmissão sináptica. As mudanças de longa duração na excitabilidade se assemelham em longo prazo à potenciação ou à depressão da intensidade das sinapses glutamatérgicas [10, 16, 17, 21, 25].

Evidências sugerem que a aplicação da ETCC no córtex motor primário modula a dor através de efeitos corticais diretos nos núcleos talâmicos anterior e ventro-lateral, tálamo medial, giro cingulado anterior e tronco [26, 27]. Ensaios clínicos preliminares sugeriram que a ETCC anodal do córtex motor primário pode ser eficaz no tratamento de síndromes dolorosas crônicas tais como fibromialgia [28], lesão medular [12] e dor pélvica crônica [29, 30]. Além disso, a ETCC produziu aumento nos limiares de dor em modelo de dor experimental [31]. Em estudos experimentais com animais conduzidos pelo nosso grupo observou-se que a ETCC reverteu a hiperalgesia induzida pelo estresse crônico e pela dor inflamatória articular [32]. No entanto, o efeito da ETCC no estado hiperalgésico de pacientes com dor crônica, assim como o seu potencial efeito na ansiedade e na reabilitação pós-operatoria não foram explorados.

No contexto perioperatório, estudos de pequeno porte têm demonstrado o potencial benefício das técnicas de neuroestimulação. Como exemplo, temos o estudo que avaliou o efeito de uma única sessão de estimulação magnética transcraniana repetitiva (EMTr) em pacientes submetidos ao bypass gástrico, o qual demontrou que os pacientes tratados tiveram redução no consumo de opióides no período pós-operatório[33]. A redução no consumo de opióides com o uso da ETCC foi demonstrada em cirurgias da coluna lombar [34] e no pós-operatório de artroplastia total de joelho [35]. Uma única sessão de 20 minutos teve impacto na redução do consumo de opióides no pós-operatório de procedimentos endoscópicos [36]. A incidência de efeitos adversos da ETCC é baixa, e inclui: cefaléia, tontura, náusea, sensação de coceira, eritema ou irritação cutânea no local

de aplicação dos eletrodos [18]. No entanto, ainda não foi explorado o efeito da ETCC na hiperalgesia pré-operatória de pacientes sensibilizados, na ansiedade e na reabilitação pós-operatória de pacientes submetidas à cirurgia para correção de halux valgus. Neste estudo optou-se pelo uso da ETCC para estimular o córtex cerebral por ter eficácia demonstrada em síndromes dolorosas, por ser uma técnica não invasiva, apresentar baixo custo, ser de fácil aplicação em comparação com outras técnicas de neuroestimulação e, sobretudo, pelo seu potencial de contra-regular as alterações neuroplásticas mal-adaptativas associadas à dor crônica.

Considerando o acúmulo de evidências sobre os benefícios dessa técnica e baixo potencial de efeitos adversos, novas pesquisas devem ser apoiadas em vista da necessidade da obtenção de dados que proporcionem a sua correta indicação, sobretudo em pacientes sensibilizados pelo quadro álgico sustentado. Nossa hipótese é que a ETCC possa auxiliar no tratamento da dor pós-operatória (EAV, limiar de dor e CPM) de correções cirúrgicas de hálux valgo, na redução da ansiedade pré-operatória e no curso da reabilitação pós-operatória.

2. Revisão da Literatura

2.1 Estratégias para localizar e selecionar as informações

Nesta revisão da literatura, pretende-se abordar alguns aspectos sobre a relação entre a dor crônica de pacientes com hálux valgus, o seu tratamento cirúrgico, e a ETCC. A estratégia de busca envolveu as seguintes bases de dados: MEDLINE (site PubMed), LILACS, SCIELO, Biblioteca Digital Brasileira de Teses e Dissertações. Livros-texto de ortopedia também foram consultados.

As referências bibliográficas dos artigos identificados foram revisadas para localizar outras não contempladas na busca. No site PubMed, LILACS e SCIELO foram realizadas buscas através dos termos *hallux valgus*, *bunion pain*, *metatarsal head osteotomy, post operative pain, pre operative period* e *tDCS*. Em relação ao termo *hallux valgus* foram encontrados 3074 artigos no PUBMED, 108 no LILACS e 21 no SCIELO. Usando o termo *bunion pain* encontrou-se 4 artigos no PUBMED, 9 no LILACS e nenhum no SCIELO; o termo *metatarsal head osteotomy*, 321 artigos no PUBMED, nenhum no LILACS ou no SCIELO; o termo *post operative pain*, 72313 artigos no PUBMED, nenhum no LILACS e 166 no SCIELO; o termo *pre operative period*, 5156 artigos no

PUBMED, nenhum no LILACS e 206 no SCIELO e em relação a *tDCS* foram encontrados 1277 no PUBMED, 10 no LILACS e 07 no SCIELO.

Refinando-se a busca, com o cruzamento das palavras-chave *hallux valgus* e *metatarsal head osteotomy*, foi gerado um pool de 223 artigos no PUBMED, 1 artigo no LILACS e nenhum no SCIELO. Do cruzamento dos termos *tDCS* e *bunion pain*, dos termos *tDCS* e *metatarsal head osteotomy*, e dos termos *tDCS* e *pre operative period* não foram encontrados artigos em nenhum dos bancos de dados. Combinando *tDCS* e *post operative pain* obtivemos 6 artigos no PUBMED e nenhum nos outros bancos de dados. Do banco de teses, foram localizadas 19 teses com o tema tDCS. Para apresentar o tema, usamos a revisão sistemática esquematizada na figura 1.

Figura 1 - Estratégia de busca de referências bibliográficas sobre as bases que fundamentam os objetivos deste estudo.

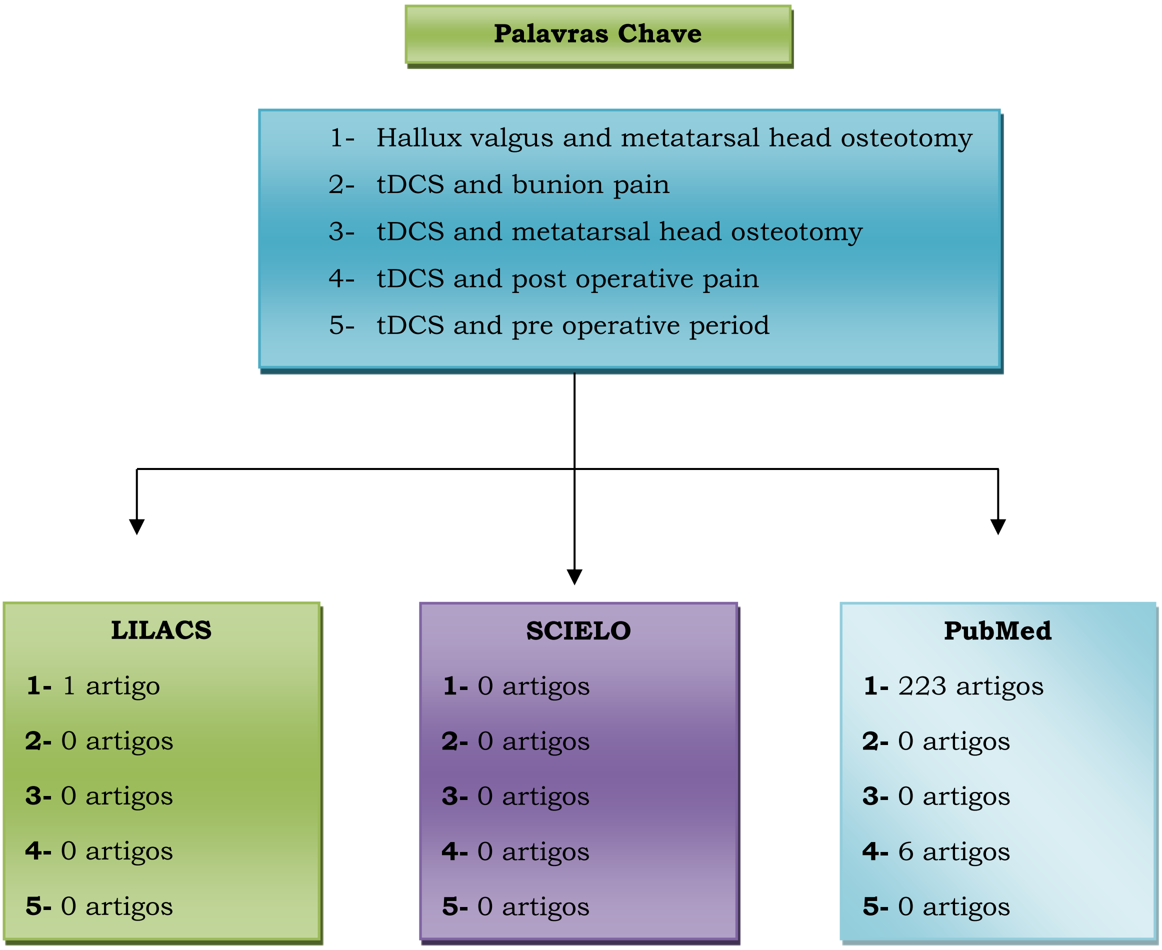

2.2 Aspectos históricos e conceituais

Hálux valgo é um problema comum que acomete o antepé, no qual a 1ªAMF subluxa progressivamente, com desvio lateral do hálux e desvio medial do primeiro metatarso [37]. Frequentemente é acompanhado de proeminência medial dolorosa na cabeça do primeiro metatarso, conhecida como joanete. Conforme a deformidade progride, o desvio lateral do hálux interfere no alinhamento e dinâmica dos dedos menores, resultando em deformidades em garra, distribuição anormal das cargas plantares e desenvolvimento de calosidades plantares. A pressão exercida pela proeminência medial nos calçados pode levar à formação de bursite adventícia, usualmente dolorosa e inflamada. A causa do hálux valgo não é absolutamente compreendida. Existem algumas evidências de que esta condição é autossômica dominante. Outros fatores que podem contribuir são o uso de sapatos constritivos e fatores estruturais do pé.[37]

2.3 Abordagem terapêutica da artralgia metatarso-falangeana

Alguns portadores de hálux valgo toleram a dor gerada pela presença da deformidade ao adequar seus calçados para um formato mais favorável. Os calçados com uma câmara anterior larga e alta podem ser os mais eficazes para esse fim. Inúmeros dispositivos com o intuito de proteção da deformidade já foram disponibilizados no mercado com o intuito de redução dos sintomas, tornando possível a abordagem conservadora, porém nenhum deles é capaz de corrigir a deformidade de maneira definitiva. Dessa forma, para atingir a cura da deformidade, faz-se necessário o tratamento cirúrgico.

Os primeiros relatos de uma osteotomia distal da cabeça do metatarso são de 1881, por Riverdin, o qual descreveu uma osteotomia subcapital com cunha de subtração para a correção do hálux valgo[38]. A osteotomia de Chevron é uma das técnicas mais comuns para o tratamento cirúrgico do hálux valgo leve e moderado (Figura 2). Em seus primeiros relatos não foi mencionada nenhuma necessidade de fixação. Assim, sugeriu-se que o formato da osteotomia e o impactação da cabeça esponjosa sobre o eixo do primeiro metatarso traria estabilidade suficiente[39]. Essa técnica é contraindicada naqueles pacientes que apresentam artrose da 1ªAMF do pé acometido e têm instabilidade metatarso-cuneiforme.

Figura 2. Ilustração da técnica de Chevron

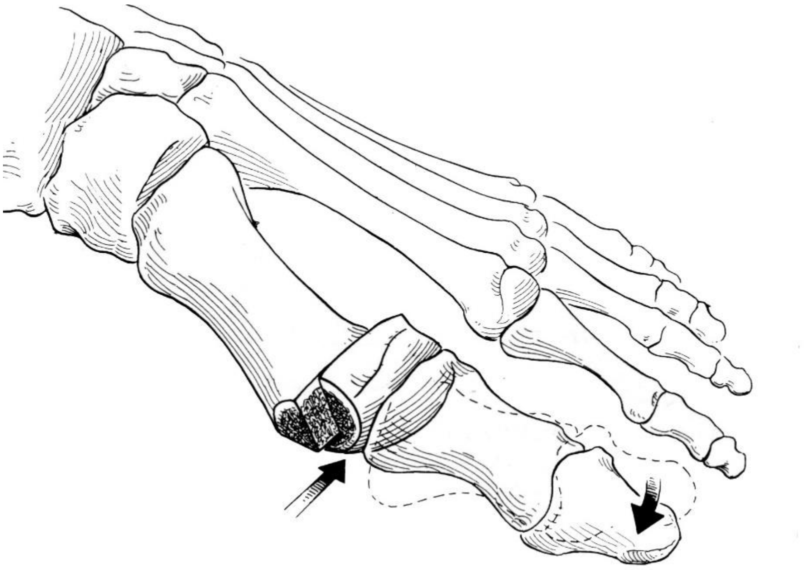

Posteriormente, para aumentar a indicação para esta osteotomia tecnicamente simples, foram adicionadas fixação interna, associação com liberação das estruturas laterais da cabeça metatarsal e até outras osteotomias realizadas concomitantemente[40]. Opcionalmente, com o intuito de "potencializar" a correção do hálux valgo, associa-se uma segunda osteotomia, a osteotomia de Akin, a qual é realizada na falange proximal do hálux, subtraindo-se uma cunha de base medial. (Figura 3)

Figura 3. Ilustração da técnica de Akin

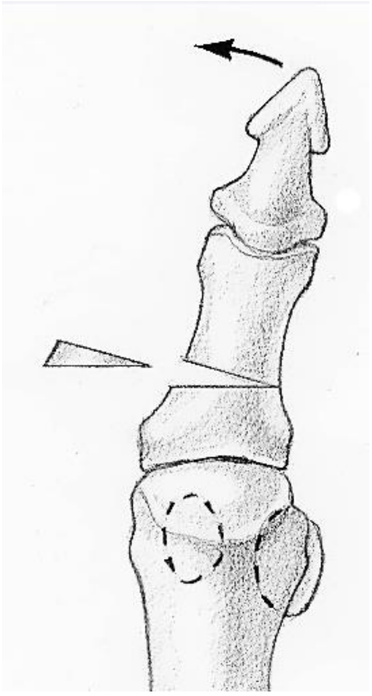

A osteotomia de Akin, também realizada com uma única incisão longitudinal medial, permite o alinhamento no eixo do hálux mesmo quando existe acentuada inclinação lateral da cabeça em relação à diáfise do primeiro metatarso. Nestes casos o eixo do hálux é corrigido de forma espúria, mas o resultado clínico é amplamente satisfatório, tanto do ponto de vista estético quanto funcional. A combinação dessas técnicas permite, também, a correção da deformidade rotacional do hálux. Durante a execução da osteotomia transversa na base da falange proximal é possível girar o hálux no sentido contrário e corrigir a pronação excessiva[41].

Os cuidados pós-operatórios e o período de recuperação das técnicas combinadas são similares ao da técnica de Chevron isolada. No estudo de Costa e col. [42] foi empregada a combinação de ambas as técnicas no tratamento cirúrgico do hálux valgo em 29 pacientes (47 hálux valgos), a qual mostrou resultado clínico-funcional satisfatório em 46 hálux valgos após tempo médio de seguimento de 60 meses.

2.4 Técnica Cirúrgica

Ambas osteotomias (Chevron e Akin) são realizadas pela mesma incisão, que deve ser aplicada no aspecto medial do antepé, desde a falange proximal do hálux até o terço distal do primeiro metatarso. Em seguida realiza-se cuidadosa dissecção dos planos e excisão da cápsula articular sobressalente. Dessa forma, torna-se visível a exostose medial da cabeça do metatarso, a qual deve ser removida em alinhamento com o bordo medial do pé.

Com a exostose removida, realiza-se a osteotomia de Chevron distal da cabeça metatarsal, que pode ser fixada com os mais diversos implantes. No nosso serviço, costumamos utilizar parafusos de tipo cortical com espessura de 2 milímetros para tal fixação.

Na região mais distal da incisão, aborda-se a falange proximal do hálux em um plano subperiosteal, tomando cuidado para proteger os tendões flexor e extensor longos do hálux. Utilizando serra oscilante, retira-se uma cunha de base medial com espessura suficiente para completar a correção do hálux valgo, tornando o hálux completamente alinhado com o primeiro raio. Posteriormente, procede-se a fixação com o mesmo tipo de implante.

Com efeito, deve-se tomar um cuidado maior ao suturar e retensionar a cápsula articular do primeiro metatarso. Afinal, a tensão excessiva pode gerar restrição da mobilidade e artrose, ao mesmo tempo em que a tensão insuficiente pode facilitar a recidiva da deformidade. [41-43]

2.5 Neuroplasticidade – Processo e marcadores

Pacientes portadores de dor crônica sofrem alterações do sistema nervoso quanto à localização e à expressão de canais iônicos, de receptores e de sinapses nervosas. As mudanças da distribuição e da cinética de neurotransmissores e de neuromediadores permitem que os neurônios centrais ou periféricos atinjam o limiar para despolarização mais precocemente, gerando descargas ectópicas que se amplificam e ativam células vizinhas. Pode-se inferir, então, que a dor crônica é um estado de constante facilitação da condução nervosa, quando estímulos que outrora inócuos podem ser interpretados como dor (alodínia) ou quando a resposta ao estímulo doloroso não é proporcional à intensidade da agressão (hiperalgesia) [44]. Este processo envolve mecanismos de neuroplasticidade como formação dendrítica, remodelação sináptica, *Long Term Potentiation* (LTP), desenvolvimento axonal, extensão neurítica, sinaptogênese e neurogênese, pelos quais o cérebro se adapta e responde a uma variedade de estímulos internos e externos [45]. Sendo assim, a artralgia crônica da primeira articulação metatarso-falangeana induzida por hálux valgo é um campo que pode ser explorado através de técnicas modulatórias que apresentam efeitos significativos e duradouros.

O Fator de Crescimento Neural (NGF) foi identificado primeiramente como um fator de sobrevida para neurônios sensoriais e simpáticos no desenvolvimento do sistema nervoso. Em adultos, não é necessário para a sobrevida neural, porém desempenha papel importante na geração de dor e hiperalgesia aguda e em outros processos de dor crônica. A expressão do NGF é intensa em tecidos lesionados e inflamados e desencadeia em neurônios nociceptivos a potencialização da sinalização da dor por vários mecanismos. O NGF é membro fundador da família de proteínas neurotrofinas relacionadas estruturalmente ao fator neurotrófico derivado do cérebro (BDNF), neurotrofina 3 (NT-3) e NT-4. Outra proteína que age como fator neurotrófico evidenciando lesão no SNC é a S100 β, um marcador que desempenha um papel no metabolismo energético, na regulação do ciclo, no crescimento, na diferenciação e na motilidade celular[46]. A S100 β exerce efeitos tróficos ou tóxicos, dependendo da sua concentração. Em mínimas concentrações, ela estimula o crescimento neuronal, incrementa a sobrevida dos

neurônios durante o desenvolvimento e age como fator neurotrófico e proteína de sobrevivência neuronal, em resposta à ativação glial secundária à lesão. Por outro lado, em altas concentrações a S100 β exacerba a neuroinflamação e a disfunção neuronal, com estímulo de citocinas pró-inflamatórias e indução de apoptose[47]. Estudos demonstraram que esse fator neurotrófico pode estar implicado na depressão e no desempenho cognitivo e na dor [47-49]. Já o BDNF é uma neurotrofina abundantemente expressa em cérebro adulto e em medula espinhal, desempenhando um importante papel na regulação do limiar de dor inflamatória e da hiperalgesia secundária[50]. O BDNF não é definido como um promotor exclusivo de inibição (GABA) ou excitabilidade (glutamato) neuronal, uma vez que exerce um papel modulador na neurotransmissão dependente de atividade [51]. Então, esta neurotrofina envolvida no processo de plasticidade neuronal, participa do processo de potenciação sináptica de longa duração (LTP), mecanismo de neuroplasticidade que sustenta o processo de memória dolorosa [52]. Além disso, o BDNF tem efeitos difusos no sistema de neurotransmissão monoaminérgica. Promove "sprouting" dos neurônios serotoninérgicos maduros, aumentando a atividade serotoninérgica central que se segue à infusão deste, modulando a ativação dos neurônios do núcleo dorsal da rafe e a função transportadora serotoninérgica. O BDNF também influencia a atividade dopaminérgica.

Esse conjunto de evidências demonstra que o estabelecimento de um quadro de dor crônica é mediado por múltiplos mecanismos que induzem uma série de adaptações de médio e longo prazo, envolvendo modificações neurais e endócrinas [53]. Cabe ressaltar também que esses fatores podem regular ou alterar a liberação de glutamato e GABA em processos dolorosos [54]. Sendo que, a síntese desses neurotransmissores são originadas pela isoenzima ácido glutâmico descarboxilase (GAD) [55], que sintetiza a partir do glutamato e piridoxa-5'fosfato (PLP) como cofator, o neurotransmissor GABA [56].

2.6 Neuroplasticidade e efeito da neuroestimulação

Para compreender o efeito das diferentes terapêuticas no processo de neuroplasticidade cerebral utilizaremos parâmetros fisiológicos (moduladores descendentes de dor), e marcadores de neuroplasticidade como o BDNF (plasticidade neuronal). O BDNF regula a integridade e diferenciação dos neurônios durante o desenvolvimento, incluindo o processo de plasticidade neuronal. A expressão do BDNF no SNC é modificada por acometimentos diversos, tais como estresse, isquemia,

hipoglicemia, depressão e dor. O BDNF tem efeitos sinápticos importantes: aumenta a freqüência espontânea de potenciais de ação nos neurônios, potencializa circuitos inibitórios e excitatórios, interfere na neuromodulação de inter-neurônios gabaérgicos, colinérgicos, dopaminérgicos e noradrenérgicos. [57]. Esta neurotrofina atua em mecanismos adaptativos essenciais, exercendo papel crucial no processo de potenciação sináptica de longa duração (LTP), mecanismo de neuroplasticidade fundamental para desencadear e sustentar o processo de memória da dor. O aumento de BDNF incrementa a LTP, enquanto que a redução de seus níveis atenua este fenômeno. Portanto a relação de níveis séricos com a severidade da doença pode confirmar a influência sistêmica desse biomarcador em estados de dor sustentada.

As potenciais vantagens das terapias neuromodulatórias não invasivas além de serem mais eficazes e apresentarem baixo custo, podem se constituir como modalidade terapêutica adicional a ser disponibilizada à sociedade. No entanto, é importante avaliar seus efeitos usando evidências derivadas do efeito clínico combinado com informações por meio de marcadores plasmáticos, psicofísicos e eletrofisiológicos. Este estudo acrescentará na literatura informações sobre a composição do líquido cefalorradiano em pacientes submetidos às terapias neuromodulatórias.

2.7 Estimulação Transcraniana por Corrente Contínua (ETCC) e artralgia metatarso-falangeana

A ETCC é uma técnica de estimulação cerebral de baixo custo, não-invasiva, e de fácil operação em comparação com outras técnicas de neuromodulação. Ela consiste na aplicação de uma corrente direta sobre o couro cabeludo utilizando eletrodos que são circundados por esponjas perfuradas umedecidas com solução salina ou eletrodos de borracha com gel condutor [58]. Seus efeitos dependem do tamanho, da polaridade (anódica ou catódica) e posição dos eletrodos, intensidade da corrente aplicada, duração do estímulo e das propriedades do tecido da área estimulada. A estimulação anódica aumenta a excitabilidade cortical, enquanto a estimulação catódica a diminui [18]. O mecanismo de ação da ETCC é multifatorial e induz alterações fisiológicas em diferentes sistemas, localizadas ou distantes do foco de aplicação. Alguns estudos exploratórios identificaram até o presente momento diferentes efeitos da ETCC sobre a neurotransmissão, vias neuroquímicas e marcadores neurobiológicos como a modulação da atividade glutamatérgica [59, 60], GABAérgica [61, 62], dopaminérgica [63-65],

serotonérgica [66] e colinérgica [67, 68], além do efeito sobre a permeabilidade de diferentes canais de membrana como sódio e cálcio. Seu benefício já foi demonstrado em várias condições patológicas como dor pélvica crônica [30], depressão[69], acidente vascular cerebral (AVC), doença de Parkinson [67]. Também foram demonstrados seus efeitos imediatos e duradouros em modelo animal de dor subaguda relacionada à inflamação crônica. [70] Todavia, seu uso com a finalidade de controle de dor em períodos peri-operatórios ainda precisa ser melhor estudado. Uma única sessão de estimulação magnética transcraniana repetitiva (EMTr) foi associada à redução no consumo de opióides no período pós-operatório de bypass gástrico[33]. Já a ETCC, apesar de não ter demonstrado benefício no período pós-operatório de cirurgias em coluna lombar [34], já demonstrou redução do consumo de opióides no período pós-operatório de artroplastia total de joelho [35] e uma única sessão de 20 minutos já demonstrou redução no consumo de opióides no período pós-operatório de procedimentos endoscópicos, além de demontrar segurança e mínimos efeitos adversos[36]. O presente estudo visa avaliar o efeito da ETCC aplicada no período pré-operatório como método coadjuvante no controle da dor pós-operatória de correção cirúrgica de hálux valgus em pacientes com artralgia da 1ªAMF.

3. Justificativa

Considerando o acúmulo de evidências sobre os benefícios dessa técnica, seus raros efeitos adversos, sua fácil aplicação em comparação com outras técnicas neuromodulatórias, a prevalência de artralgia em pacientes portadores de hálux valgo, a relevância social da impossibilidade de calçar sapatos devido à artralgia e a possibilidade de inclusão de um novo método de analgesia para períodos peri-operatórios, estudos inovadores que possam proporcionar avanços no processo terapêutico da artralgia da primeira articulação metatarso-falangeana são justificados. Caso a hipótese da eficácia da ETCC neste período seja confirmada, poder-se-á obter grande avanço no cenário da analgesia multimodal e redução de sofrimento dos indivíduos submetidos a procedimentos cirúrgicos. O interesse nesta patologia pauta-se na premissa de que esta condição dolorosa crônica constitui grande parcela da procura ao atendimento médico especializado em ortopedia.

4. Objetivo

4.1 Objetivo Principal

Avaliar o efeito da ETCC comparada ao ETCC-sham no controle da dor, reabilitação e ansiedade peri-operatória em pacientes com artralgia da 1ªAMF submetidas a tratamento cirúrgico de hálux valgus.

4.2 Objetivos Secundários

- Avaliar o efeito da ETCC comparado ao ETCC-sham no tratamento da dor pós-operatória de correções cirúrgicas de hálux valgo, nos seguintes desfechos:

- níveis de dor na escala numérica da dor (END) de 0-10 nos 8 primeiros dias pós-operatórios.

- Avaliar a sensibilização periférica de nociceptores através de estímulos algogênicos padronizados induzidos pelo teste sensorial quantitativo (QST, do inglês *quantitative sensory test*).

- Função do sistema modulador descendente ao estímulo heterotópico.

- Consumo de analgésicos.

- Escala funcional de dor.

- Níveis de pensamento catastrófico.

- Niveis séricos de BDNF e S100B.

- Niveis liquóricos de BDNF pós estimulação transcraniana.

5. Materiais e métodos

5.1 Delineamento geral: Ensaio clínico, randomizado, duplo-cego, em paralelo, controlado com placebo-sham.

5.2 Logística: O recrutamento, intervenções e acompanhamento dos pacientes do presente projeto de pesquisa será realizado no Hospital Independência de Porto Alegre, à beira do leito de enfermaria nos pacientes internados para procedimento cirúrgico. As análises laboratoriais serão realizadas no Hospital de Clínicas de Porto Alegre. Todos os participantes assinarão um termo de consentimento livre e esclarecido. O estudo será registrado na Base de Dados – Plataforma Brasil, clinicaltrials.gov e sua montagem segue o CONSORT (Consolidated Standards of Reporting Trials).**5.3 Amostragem:** Serão

incluídas pacientes do sexo feminino submetidas a tratamento cirúrgico de hálux valgus sob técnica combinada de Chevron + Akin.

5.3.1 Critérios de inclusão: idade acima de 18 anos e menores que 70 anos, sexo feminino, indicação de tratamento cirúrgico de hálux valgus por artralgia da 1ªAMF, capazes de compreender e escrever a língua portuguesa.

5.3.2 Critérios de exclusão: pacientes com neuropatia diabética, presença de contraindicações para o ETCC [58] (história de cefaléia intensa ou frequente, desordens dermatológicas crônicas, reações adversas a tratamento prévio com ETCC, história de crises convulsivas, trauma crânio encefálico grave com alteração da anatomia craniana, implantes metálicos intracranianos ou marcapasso), doença psiquiátricas não compensadas ou pacientes não cooperativas, história de doença neurológica, oncológica, cardiopatia isquêmica, insuficiências renal ou hepática.

5.4 Tamanho da amostra: O tamanho amostral foi estimado considerando como desfecho primário a média cumulativa dos escores de pior dor diária na EAV no curso das primeiras 48h de pós-operatório. Estima-se que serão necessários 28 pacientes, que serão alocados aleatoriamente em 2 grupos de 14. Prevendo-se possíveis perdas e os múltiplos desfechos, aumentou-se o tamanho amostral para 40 pacientes (20 por grupo). O número de pacientes foi definido para detectar uma redução nos escores de dor, com ETCC, de no mínimo 1,5cm na EAV (0-10 cm) com um desvio padrão de 0.9 cm. Essa diferença entre médias tem sido considerada clinicamente relevante no contexto de tratamento da dor aguda pós-operatória em diferentes cenários [71, 72]. Aceita-se uma chance de erro alfa de 0,5 e de erro beta de 20%.

5.5 Randomização e cegamento: A randomização será feita em blocos de quatro, dividindo as pacientes em dois grupos: ETCC ou ETCC-sham. A tabela de randomização será gerada por website de randomização (www.randomization.com). Os códigos da randomização ficarão aos cuidados de um pesquisador independente, que será o responsável pela aplicação da intervenção e será o único profissional que não será cegado. Este profissional não participará da seleção, aferição ou avaliação dos desfechos. Todas as pacientes e os demais profissionais, incluindo os avaliadores dos desfechos, serão cegados. Os códigos de randomização serão colocados em envelopes pardos, lacrados e rubricados pelo pesquisador responsável para evitar qualquer possibilidade de violação. Os envelopes serão numerados externamente para indicar a sequência previamente definida na randomização. O cegamento será mantido em todas as fases do estudo.

5.6 Intervenção

Aplicação da ETCC - A ETCC ou estimulação *sham* (grupo controle) serão aplicadas no crânio das pacientes em sessões de 20 minutos realizadas na noite anterior ao procedimento cirúrgico e na manhã do dia do procedimento, totalizando 2 sessões, da seguinte maneira:

a) Estimulação ativa - será aplicada por um par de eletrodos de superfície revestidos com esponja e embebidos em solução salina. Um estimulador de corrente constante movido à bateria será utilizado para esse fim (tDCS device Soterix 1X1). A estimulação será realizada colocando-se o eletrodo de estímulo anodal no córtex motor primário (M1) – área que corresponde à área C3 (EEG 10/20 system) e o catodal sobre a área supraorbital contralateral. A montagem será a padronizada em nosso laboratório, a qual segue a utilizada por Fregni e colaboradores [12, 73]. A estimulação ativa será realizada com corrente de 2 mA.

b) Estimulação sham – no grupo de estímulo sham os eletrodos serão colocados nas mesmas posições que os do grupo de estimulação anodal M1, no entanto, o estimulador será programado para se desligar após 30 segundos de estimulação. Portanto os pacientes sentirão a sensação de estimulação inicial, mas não receberão corrente pelo período restante. Este método sham foi demonstrado ser eficiente no que tange ao cegamento [74]

5.7 Desfechos

5.7.1 Desfechos primários

Parâmetros clínicos:A) Dor: - escore de dor na EAV

- consumo de analgésicos no pós-op
- escala funcional de dor [75]

5.7.2 Desfechos secundários

Parâmetros clínicos:

B) Ansiedade pré-operatória será medida com a versão refinada da escala de ansiedade traço e estado (STAI), adaptada para o português brasileiro [76]. Ansiedade estado (estado transitório causado por uma situação específica) será avaliada na noite anterior ao procedimento (período basal), e logo antes do procedimento cirúrgico.

C) Avaliação do limiar de dor e do sistema modulador descendente da dor

D) Dados demográficos e comorbidades

<u>Parâmetros laboratoriais</u>:

E) Níveis de BDNF, S100B

A amostra coletada de líquor será aliquotada e armazenada a -70°C para análises posteriores. A amostra de sangue total será coletada e centrifugada, o soro será aliquotado e armazenado a -70°C para análises posteriores. As amostras serão armazenadas pelo Laboratório de Dor e Neuromodulação coordenado pelo Prof. Dr. Wolnei Caumo, colaborador desse projeto. O BDNF e S100B serão mensurados pela técnica de ELISA conforme instruções de kit comercial.

5.8 Aferições dos desfechos

<u>Parâmetros clínicos</u>:

A) Dor – será aferida pelos seguintes meios:

<u>- Escala Numérica da dor (END) de 0-10</u> incluída em um questionário padronizado cujo zero corresponde à ausência de dor e 10, dor máxima. A aferição será feita em dois escores diários: um escore para a pior dor diária em movimento e outro escore para a média da dor diária em repouso. Durante as sessões de ETCC, serão atribuídos ainda, um escore para o momento pré e outro para o momento pós-ETCC.

<u>- Escala funcional de dor</u> - O Brazilian Profile of Chronic Pain: Screen (B-PCP:S) será usado para rápida identificação da experiência multidimensional individual da dor. Ele é constituído de uma escada de severidade da dor (quatro itens; escala possível de 0-30), uma escala de interferência (seis itens; escala possível de 0-36), e uma escala de carga emocional (cinco itens, escala possível de 0-25)[75]. A importância dessas três dimensões (severidade, interferência e carga emocional) foi recentemente ressaltada pela The Initiative on Methods, Measurement, and Pain Assessment in Clinical Trials. Ele será aplicado no período basal, e no 8º dia pós operatório.

- <u>Consumo diário de analgésicos</u> suplementares nos sete primeiros dias pós-operatórios. A prescrição no período pré-operatório, pós-operatório imediato e após alta hospitalar serão padronizados e serão registrados diariamente dose, data e horário de consumo por questionário padrão.

B) Ansiedade pré-operatória será medida com a versão refinada da escala de ansiedade traço e estado (STAI), adaptada para o português brasileiro [76]. Ansiedade estado

(estado transitório causado por uma situação específica) será avaliada no período basal, e logo antes do procedimento cirúrgico.

C) Avaliação do limiar de dor e dos sistemas moduladores descendentes da dor

- *Heat Pain Test ou Quantitative Sensory Test (QST)*: uma versão computadorizada do termoteste (Heat Pain Stimulator 1.1.10, Brazil desenvolvido pelo nosso grupo) [77] será usada para determinar a máxima temperatura tolerada e o limiar de dor ao calor no lado volar do antebraço dominante e na porção medial da perna ipsilateral ao sítio cirúrgico. A temperatura inicia em 32°C, o termodo é aquecido a uma taxa de 1.0 °C/seg até no máximo 52°C quando a temperatura começa a reduzir. A paciente apertará um botão quando não tolerar mais o aumento da temperatura. O limiar de dor ao calor é determinado pela média de três avaliações. O Teste QST será aplicado no dia que antecede o procedimento cirúrgico (antes da primeira sessão de intervenção), e ao final do dia do procedimento, após regressão completa do bloqueio sensitivo induzido pela anestesia subaracnóidea. A aplicação do teste em dois sítios diferentes (antebraço e perna) possibilitará a pesquisa e diferenciação de sensibilização central ou periférica dos receptores nociceptivos.

- CPM (conditioned pain modulation): será avaliado para acessar o sistema modulatório descendente. Para isso utiliza-se um estímulo tônico nociceptivo condicionante como imersão da mão não dominante na água à temperatura de zero até 1,5°C concomitante com o estímulo térmico no antebraço dominante e avalia-se o grau de inibição que aquele estímulo heterotópico (imersão da mão na água) provoca no limiar de dor térmica aferido no antebraço dominante. Para tanto, inicialmente determina-se a média de três aferições de temperatura na qual a paciente refere dor 6/10 seguindo o padrão do teste QST. Em seguida, a mão contra-lateral é imersa na água à temperatura de zero até 1,5°C enquanto o estímulo térmico gradual é aplicado no antebraço dominante até atingir a temperatura na qual foi referida pela paciente como 6/10 na escala de dor. A média de três aferições com intervalos de 15 minutos de repouso será usada como a medida do escore de dor condicionante. A diferença será apresentada em percentual entre as médias de dor antes e após a imersão da mão na água. O Teste CPM será aplicado no dia que antecede o procedimento cirúrgico (antes da primeira sessão de intervenção), e ao final do dia do procedimento, após regressão completa do bloqueio sensitivo induzido pela anestesia subaracnoidea.

Aferição de desfechos por parâmetros laboratoriais

E) Níveis de BDNF e S100B - A coleta das amostras de sangue acontecerá em três momentos distintos: na noite anterior ao procedimento (antes da primeira sessão de ETCC), no dia da cirurgia (logo antes do procedimento anestésico para o procedimento cirúrgico) e no retorno ambulatorial (8º dia pós-operatório). A coleta de líquor será realizada durante a anestesia subaracnoidea em um único momento. O material será preparado e acondicionado em meio e ambiente apropriados imediatamente após a coleta.

5.9 Potenciais confundidores:

- **Dados demográficos e comorbidades**, assim como uso crônico de medicamentos serão avaliados por questionário padrão.

- Os **níveis de sintomas depressivos** serão aferidos pelo Inventário de Beck II[78]. Os sintomas depressivos serão aferidos no período basal.

- **O nível de pensamento catastrófico** será aferido pela escala de pensamento Catastrófico de dor [79] no período basal.

5.10 Técnica anestésica e cirúrgica5.10.1 Medicação pré-operatória – Serão preescritos medicamentos e terapias já em uso prévio pela paciente, assim como medicamentos analgésico se necessários (dipirona EV 1g 6/6h se dor, tramadol 50mg EV 6/6h se dor forte). Não serão preescritos medicamentos ansiolíticos ou analgésicos de maneira fixa.

5.10.2 Técnica anestésica - A anestesia subaracnóidea para o procedimento cirúrgico será realizada com agulha própria para esse fim (Quincke 25G) e as medicações administradas no espaço subaracnóideo, logo após coleta do líquido cefalo-raquidiano (LCR), será padronizada: bupivacaína hiperbárica 12,5mg + morfina 80mcg. Logo após a anestesia subaracnóidea, as pacientes serão levemente sedadas com 50mcg de fentanil e de 1 a 5mg de midazolan conforme necessidade individual de cada paciente para conforto no transoperatório. Doses adicionais de propofol em bolus ou propofol administrado em bomba de infusão poderão ser administrados nos casos em que a paciente mantiver-se alerta apesar dos sedativos administrados.

5.10.3 Técnica cirúrgica – Todas as pacientes serão submetidas à técnica combinada de osteotomia de Chevron + Akin após o estabelecimento da anestesia subaracnóidea. A duração prevista do procedimento é de uma hora.

5.10.4 Medicação pós-operatória - A prescrição no período pós-operatório imediato será: dipirona EV 1g 6/6h se dor, tramadol 50mg EV 6/6h se dor forte e morfina 3mg EV 3/3h se dor refratária ao tramadol. Após alta hospitalar, a prescrição domiciliar será padronizada: dipirona 1g VO 6/6h se dor, tramadol 50mg VO 6/6h se dor forte.

5.11 Efeitos Adversos – ao final de cada sessão de ETCC será questionado a ocorrência de qualquer efeito adverso como parestesia, cefaléia, tontura, náusea, dor no pescoço, queimadura, eritema ou dor no couro cabeludo, insônia, alteração abrupta do humor e dificuldade de concentração.

5.12 Acompanhamento clínico pós-operatório – as pacientes receberão visita médica uma vez por dia enquanto internadas para conferência do consumo de analgésicos e avaliação do controle álgico pelo escore EAV. Após alta hospitalar (planejada para o primeiro dia pós-operatório) as pacientes preencherão questionário padronizado com escores de EAV uma vez por dia e trarão na consulta ambulatorial programada para o oitavo dia pós-operatório.

5.13 Riscos – Os possíveis desconfortos referentes à aplicação da intervenção do presente estudo são: pequenos choques, coceira e vermelhidão na região onde serão instalados os eletrodos da ETCC e, raramente, tonturas, dor de cabeça e vertigem. Considerando a prática da anestesia raquidiana comum no atendimento de pacientes submetidos à correção cirúrgica de hálux valgo, os seguintes riscos, apesar de existentes devido a presença de punção lombar para anestesia raquidiana, foram considerados não exclusivos do participante da pesquisa: cefaléia pós-punção lombar, desconforto em sítio de punção lombar, hipotensão, infecção, sequela neurológica transitória ou permanente.

5.14 Sequência de Procedimentos

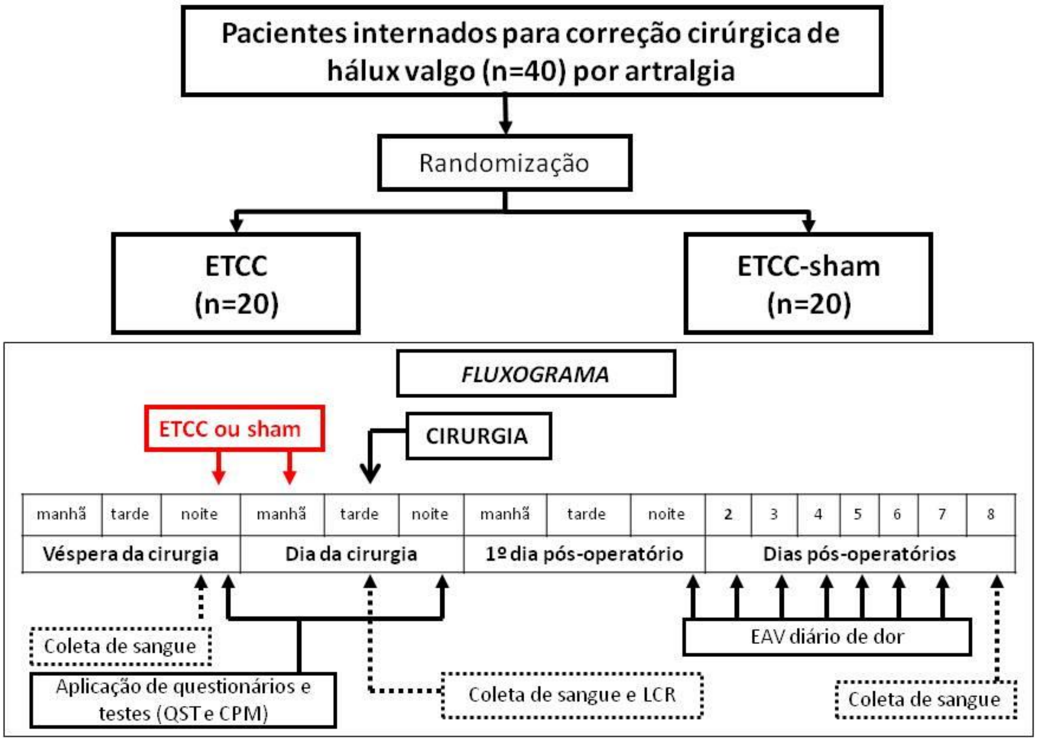

6. Processamento dos dados e análise estatística

Proceder-se-á à análise descritiva dos dados com média, frequência ou proporção. A diferença entre os grupos para variáveis contínuas será avaliada por meio do teste t, e para variáveis categóricas o teste de $\chi 2$ ou teste exato de Fisher. Após avaliar a distribuição das variáveis desfechos por meio dos testes de Skewness e Kurtosi, as variáveis desfecho que apresentarem distribuição normal ou aproximadamente normal serão comparadas entre os 2 grupos de tratamento pelo teste t de Student. Para as variáveis de desfecho que não apresentarem critério para uso de estatística paramétrica, o efeito entre os grupos de tratamento será analisado usando o teste de U de Wilcoxon-Mann-Whitney. ANOVA de medidas repetidas será usada para comparar as medidas levando em consideração o tempo (NPS) e o efeito do sujeito. Caso seja necessário, será realizada regressão linear para ajustar as variáveis confundidoras. A chance de erro α aceitável para todas as análises será de 5%. Os dados serão analisados no programa SPSS 12.0 e os gráficos no Programa SigmaPlot version 10.0.

7. Aspectos éticos

O presente protocolo segue as condições da resolução 466/12 do do Conselho Nacional de Saúde (CNS). Após o esclarecimento sobre os propósitos do estudo, as pacientes assinarão o termo de consentimento informado. O presente projeto será submetido a apreciação pelo comitê de ética do Hospital de Clínicas de Porto Alegre.

8. Exequibilidade do Projeto

Esse projeto está dentro da linha de pesquisa do Laboratório de Dor e Neuromodulação do Hospital de Clínicas de Porto Alegre. Portanto as técnicas descritas já são conhecidas e já estão sendo aplicadas em diferentes estudos. A amostra se constituirá de pacientes pertencentes ao Hospital Independência de Porto Alegre.

9. Controle de Qualidade

Será realizado treinamento da equipe para aplicação das avaliações. Serão realizadas reuniões semanais para discutir possíveis dúvidas surgidas durante a realização do protocolo. As instruções de todas as etapas e métodos de aferição constarão em um manual de instruções.

10. Orçamento detalhado

Quantidade	Descrição	Valor Unidade	Vlr. Total
6	Armazenamento de amostras (mensal)	R$ 60,00	R$ 360,00
2	Tubos de ensaio para coleta de sangue – caixa com 100	R$ 35,00	R$ 70,00
50	Frascos para coleta de LCR	R$ 1,05	R$ 52,50
48	Consultas (40 + 20% = 48)	R$ 7,55	R$ 362,40
50	Envelope - randomização e questionários	R$ 0,50	R$ 25,00
500	Impressão - cópias de questionários	R$ 0,20	R$ 100,00
2	Ponteira amarela ou incolor de 0-200ul s/ ranhura pacote com 1000	R$ 14,80	R$ 29,60
10	Ponteira p-1000 cap. 200-1000ul estéril racks de 96unid por peça	R$ 0,13	R$1,30
10	Soro Fisiológico 250ml 0,9% 0,15meq/ml	R$ 0,79	R$ 7,90
1	Tubo Eppendorf 1,5ml Pacote com 1000 unidades Estéril	R$ 40,00	R$ 40,00
2	Tubo tp Eppendorf 0,5ml pacote 500 un	R$ 30,00	R$ 60,00
1	kit ELISA BDNF humano	R$ 3.100,00	R$ 3.100,00
**TOTAL**			**R$ 4208,70**

11. Cronograma físico-financeiro

	2014											
	Jan	Fev	Mar	Abr	Mai	Jun	Jul	Ago	Set	Out	Nov	Dez
Elaboração do projeto												
Aprovação pela Comissão de Ética e Pesquisa												
Treinamento da equipe e logística												
Coleta de dados												
2015												
Coleta de dados												
Banco de dados												
Tratamento estatístico												
Redação												

12. REFERÊNCIAS

1. PA, V., *Anatomia, patologia del pie*. 3rd ed1984.
2. Nery CAS, H.S., Xavier R, Pardini Jr AG, Barros Filho TEP, *Ortopedia e traumatologia – Princípios e prática*. 3rd ed2003.
3. Cavanagh, P.R., M.M. Rodgers, and A. Iiboshi, *Pressure distribution under symptom-free feet during barefoot standing.* Foot Ankle, 1987. **7**(5): p. 262-76.
4. Coughlin, M.J. and C.P. Jones, *Hallux valgus: demographics, etiology, and radiographic assessment.* Foot Ankle Int, 2007. **28**(7): p. 759-77.
5. Melzack, R., *Pain--an overview.* Acta Anaesthesiol Scand, 1999. **43**(9): p. 880-4.
6. Casey, K.L., *Forebrain mechanisms of nociception and pain: analysis through imaging.* Proc Natl Acad Sci U S A, 1999. **96**(14): p. 7668-74.
7. Tracey, I. and P.W. Mantyh, *The cerebral signature for pain perception and its modulation.* Neuron, 2007. **55**(3): p. 377-91.
8. Ploner, M. and A. Schnitzler, *[Cortical representation of pain].* Nervenarzt, 2004. **75**(10): p. 962-9.
9. Talbot, J.D., et al., *Multiple representations of pain in human cerebral cortex.* Science, 1991. **251**(4999): p. 1355-8.
10. Boros, K., et al., *Premotor transcranial direct current stimulation (tDCS) affects primary motor excitability in humans.* Eur J Neurosci, 2008. **27**(5): p. 1292-300.
11. Floel, A., et al., *Noninvasive brain stimulation improves language learning.* J Cogn Neurosci, 2008. **20**(8): p. 1415-22.
12. Fregni, F., et al., *A sham-controlled, phase II trial of transcranial direct current stimulation for the treatment of central pain in traumatic spinal cord injury.* Pain, 2006. **122**(1-2): p. 197-209.
13. Fregni, F., et al., *A randomized, sham-controlled, proof of principle study of transcranial direct current stimulation for the treatment of pain in fibromyalgia.* Arthritis Rheum, 2006. **54**(12): p. 3988-98.
14. Hummel, F. and L.G. Cohen, *Improvement of motor function with noninvasive cortical stimulation in a patient with chronic stroke.* Neurorehabil Neural Repair, 2005. **19**(1): p. 14-9.

15. Wagner, T., et al., *Transcranial direct current stimulation: a computer-based human model study.* Neuroimage, 2007. **35**(3): p. 1113-24.
16. Nitsche, M.A., et al., *Pharmacological modulation of cortical excitability shifts induced by transcranial direct current stimulation in humans.* J Physiol, 2003. **553**(Pt 1): p. 293-301.
17. Nitsche, M.A., et al., *Modulation of cortical excitability by weak direct current stimulation--technical, safety and functional aspects.* Suppl Clin Neurophysiol, 2003. **56**: p. 255-76.
18. Nitsche, M.A., et al., *Transcranial direct current stimulation: State of the art 2008.* Brain Stimul, 2008. **1**(3): p. 206-23.
19. Auvichayapat, P. and N. Auvichayapat, *Basic knowledge of transcranial direct current stimulation.* J Med Assoc Thai, 2011. **94**(4): p. 518-27.
20. Raghavan, S., S. Eldabe, and R. Strachan, *Novel trends in pain management – Neuromodulation.* Current Anaesthesia & Critical Care, 2008. **19**: p. 344-348.
21. Nitsche, M.A. and W. Paulus, *Excitability changes induced in the human motor cortex by weak transcranial direct current stimulation.* J Physiol, 2000. **527 Pt 3**: p. 633-9.
22. Utz, K.S., et al., *Electrified minds: transcranial direct current stimulation (tDCS) and galvanic vestibular stimulation (GVS) as methods of non-invasive brain stimulation in neuropsychology--a review of current data and future implications.* Neuropsychologia, 2010. **48**(10): p. 2789-810.
23. Liebetanz, D., et al., *After-effects of transcranial direct current stimulation (tDCS) on cortical spreading depression.* Neurosci Lett, 2006. **398**(1-2): p. 85-90.
24. Siebner, H.R., et al., *Preconditioning of low-frequency repetitive transcranial magnetic stimulation with transcranial direct current stimulation: evidence for homeostatic plasticity in the human motor cortex.* J Neurosci, 2004. **24**(13): p. 3379-85.
25. Nitsche, M.A. and W. Paulus, *Sustained excitability elevations induced by transcranial DC motor cortex stimulation in humans.* Neurology, 2001. **57**(10): p. 1899-901.
26. Garcia-Larrea, L., et al., *Electrical stimulation of motor cortex for pain control: a combined PET-scan and electrophysiological study.* Pain, 1999. **83**(2): p. 259-73.
27. Strafella, A.P., Y. Vanderwerf, and A.F. Sadikot, *Transcranial magnetic stimulation of the human motor cortex influences the neuronal activity of subthalamic nucleus.* Eur J Neurosci, 2004. **20**(8): p. 2245-9.
28. Arnold, L.M., *Biology and therapy of fibromyalgia. New therapies in fibromyalgia.* Arthritis Res Ther, 2006. **8**(4): p. 212.
29. Lefaucheur, J.P., *Transcranial magnetic stimulation in the management of pain.* Suppl Clin Neurophysiol, 2004. **57**: p. 737-48.
30. Fenton, B.W., et al., *A preliminary study of transcranial direct current stimulation for the treatment of refractory chronic pelvic pain.* Brain Stimul, 2009. **2**(2): p. 103-7.
31. Reidler, J.S., et al., *Effects of motor cortex modulation and descending inhibitory systems on pain thresholds in healthy subjects.* J Pain, 2012. **13**(5): p. 450-8.
32. Spezia Adachi, L.N., et al., *Reversal of chronic stress-induced pain by transcranial direct current stimulation (tDCS) in an animal model.* Brain Res, 2012. **1489**: p. 17-26.
33. Borckardt, J.J., et al., *Postoperative left prefrontal repetitive transcranial magnetic stimulation reduces patient-controlled analgesia use.* Anesthesiology, 2006. **105**(3): p. 557-62.
34. Dubois, P.E., et al., *Postoperative analgesic effect of transcranial direct current stimulation in lumbar spine surgery: a randomized control trial.* Clin J Pain, 2013. **29**(8): p. 696-701.
35. Borckardt, J.J., et al., *Transcranial direct current stimulation (tDCS) reduces postsurgical opioid consumption in total knee arthroplasty (TKA).* Clin J Pain, 2013. **29**(11): p. 925-8.

36. Borckardt, J.J., et al., *Feasibility, safety, and effectiveness of transcranial direct current stimulation for decreasing post-ERCP pain: a randomized, sham-controlled, pilot study.* Gastrointest Endosc, 2011. **73**(6): p. 1158-64.
37. Menz, H.B., et al., *Impact of hallux valgus severity on general and foot-specific health-related quality of life.* Arthritis Care Res (Hoboken), 2011. **63**(3): p. 396-404.
38. J, R., *De la deviation en dehors du gros orl (hálux valgus) et son traitement chirurgical.* Trans Int Med Cong, 1881.
39. Mann, R.A. and K.C. Donatto, *The chevron osteotomy: a clinical and radiographic analysis.* Foot Ankle Int, 1997. **18**(5): p. 255-61.
40. Schneider, W., et al., *Chevron osteotomy in hallux valgus. Ten-year results of 112 cases.* J Bone Joint Surg Br, 2004. **86**(7): p. 1016-20.
41. Tollison, M.E. and D.E. Baxter, *Combination chevron plus Akin osteotomy for hallux valgus: should age be a limiting factor?* Foot Ankle Int, 1997. **18**(8): p. 477-81.
42. RAL, C.M.C.P.F.G.F.R.S., *Correção do hálux valgo leve ou moderado utilizando as osteotomias de Akin e chevron combinadas.* Rev. Bras. Med., 2010(67): p. 11-16.
43. Trnka, H.J., et al., *The chevron osteotomy for correction of hallux valgus. Comparison of findings after two and five years of follow-up.* J Bone Joint Surg Am, 2000. **82-A**(10): p. 1373-8.
44. Rocha, A.P., et al., *Pain: current aspects on peripheral and central sensitization.* Rev Bras Anestesiol, 2007. **57**(1): p. 94-105.
45. Manji, H.K. and R.S. Duman, *Impairments of neuroplasticity and cellular resilience in severe mood disorders: implications for the development of novel therapeutics.* Psychopharmacol Bull, 2001. **35**(2): p. 5-49.
46. Marenholz, I., C.W. Heizmann, and G. Fritz, *S100 proteins in mouse and man: from evolution to function and pathology (including an update of the nomenclature).* Biochem Biophys Res Commun, 2004. **322**(4): p. 1111-22.
47. Tanga, F.Y., et al., *Role of astrocytic S100beta in behavioral hypersensitivity in rodent models of neuropathic pain.* Neuroscience, 2006. **140**(3): p. 1003-10.
48. Arts, B., et al., *S100 and impact of ECT on depression and cognition.* J ECT, 2006. **22**(3): p. 206-12.
49. Grabe, H.J., et al., *Neurotrophic factor S100 beta in major depression.* Neuropsychobiology, 2001. **44**(2): p. 88-90.
50. Zhou, Z., et al., *Brain-specific phosphorylation of MeCP2 regulates activity-dependent Bdnf transcription, dendritic growth, and spine maturation.* Neuron, 2006. **52**(2): p. 255-69.
51. Schinder, A.F. and M. Poo, *The neurotrophin hypothesis for synaptic plasticity.* Trends Neurosci, 2000. **23**(12): p. 639-45.
52. Kossel, A.H., et al., *A caged Ab reveals an immediate/instructive effect of BDNF during hippocampal synaptic potentiation.* Proc Natl Acad Sci U S A, 2001. **98**(25): p. 14702-7.
53. Grider, M.H., et al., *In situ expression of brain-derived neurotrophic factor or neurotrophin-3 promotes sprouting of cortical serotonergic axons following a neurotoxic lesion.* J Neurosci Res, 2005. **82**(3): p. 404-12.
54. Jovanovic, J.N., et al., *Synapsins as mediators of BDNF-enhanced neurotransmitter release.* Nat Neurosci, 2000. **3**(4): p. 323-9.
55. Wingo, W.J. and J. Awapara, *Decarboxylation of L-glutamic acid by brain.* J Biol Chem, 1950. **187**(1): p. 267-71.
56. Fenalti, G. and M.J. Rowley, *GAD65 as a prototypic autoantigen.* J Autoimmun, 2008. **31**(3): p. 228-32.
57. Shirayama, Y., et al., *Brain-derived neurotrophic factor produces antidepressant effects in behavioral models of depression.* J Neurosci, 2002. **22**(8): p. 3251-61.
58. DaSilva, A.F., et al., *Electrode positioning and montage in transcranial direct current stimulation.* J Vis Exp, 2011(51).

59. Stagg, C.J., et al., *Polarity-sensitive modulation of cortical neurotransmitters by transcranial stimulation.* J Neurosci, 2009. **29**(16): p. 5202-6.
60. Antal, A., W. Paulus, and M.A. Nitsche, *Electrical stimulation and visual network plasticity.* Restor Neurol Neurosci, 2011. **29**(6): p. 365-74.
61. Stagg, C.J. and M.A. Nitsche, *Physiological basis of transcranial direct current stimulation.* Neuroscientist, 2011. **17**(1): p. 37-53.
62. Nitsche, M.A., et al., *GABAergic modulation of DC stimulation-induced motor cortex excitability shifts in humans.* Eur J Neurosci, 2004. **19**(10): p. 2720-6.
63. Nitsche, M.A., et al., *Dopaminergic modulation of long-lasting direct current-induced cortical excitability changes in the human motor cortex.* Eur J Neurosci, 2006. **23**(6): p. 1651-7.
64. Nitsche, M.A., et al., *D1-receptor impact on neuroplasticity in humans.* J Neurosci, 2009. **29**(8): p. 2648-53.
65. Monte-Silva, K., et al., *Dosage-dependent non-linear effect of L-dopa on human motor cortex plasticity.* J Physiol, 2010. **588**(Pt 18): p. 3415-24.
66. Nitsche, M.A., et al., *Serotonin affects transcranial direct current-induced neuroplasticity in humans.* Biol Psychiatry, 2009. **66**(5): p. 503-8.
67. Medeiros, L.F., et al., *Neurobiological effects of transcranial direct current stimulation: a review.* Front Psychiatry, 2012. **3**: p. 110.
68. Thirugnanasambandam, N., et al., *Isometric contraction interferes with transcranial direct current stimulation (tDCS) induced plasticity: evidence of state-dependent neuromodulation in human motor cortex.* Restor Neurol Neurosci, 2011. **29**(5): p. 311-20.
69. Nitsche, M.A., et al., *Treatment of depression with transcranial direct current stimulation (tDCS): a review.* Exp Neurol, 2009. **219**(1): p. 14-9.
70. Laste, G., et al., *After-effects of consecutive sessions of transcranial direct current stimulation (tDCS) in a rat model of chronic inflammation.* Exp Brain Res, 2012. **221**(1): p. 75-83.
71. KAS, C., *Another decision to evaluate: choice of standardized mean difference effect size estimator*, in *Sixth International Cochrane Colloquium*1998: Baltimore, MD.
72. Brattwall, M., I. Turan, and J. Jakobsson, *Pain management after elective hallux valgus surgery: a prospective randomized double-blind study comparing etoricoxib and tramadol.* Anesth Analg, 2010. **111**(2): p. 544-9.
73. Valle, A., et al., *Efficacy of anodal transcranial direct current stimulation (tDCS) for the treatment of fibromyalgia: results of a randomized, sham-controlled longitudinal clinical trial.* J Pain Manag, 2009. **2**(3): p. 353-361.
74. Gandiga, P.C., F.C. Hummel, and L.G. Cohen, *Transcranial DC stimulation (tDCS): a tool for double-blind sham-controlled clinical studies in brain stimulation.* Clin Neurophysiol, 2006. **117**(4): p. 845-50.
75. Caumo, W., et al., *Cross-cultural adaptation and validation of the profile of chronic pain: screen for a Brazilian population.* Pain Med, 2013. **14**(1): p. 52-61.
76. Kaipper, M.B., et al., *Evaluation of the structure of Brazilian State-Trait Anxiety Inventory using a Rasch psychometric approach.* J Psychosom Res, 2010. **68**(3): p. 223-33.
77. Schestatsky, P., et al., *Transient decrease of sensory perception after thermoalgesic stimuli for quantitative sensory testing.* Muscle Nerve, 2007. **36**(4): p. 466-70.
78. Andrade, L., et al., *Psychometric properties of the Portuguese version of the State-Trait Anxiety Inventory applied to college students: factor analysis and relation to the Beck Depression Inventory.* Braz J Med Biol Res, 2001. **34**(3): p. 367-74.
79. Sehn, F., et al., *Cross-cultural adaptation and validation of the Brazilian Portuguese version of the pain catastrophizing scale.* Pain Med, 2012. **13**(11): p. 1425-35.

ANEXO 1

TERMO DE CONSENTIMENTO LIVRE E ESCLARECIDO

INSTITUIÇÃO: Hospital Independência e Hospital de Clínicas de Porto Alegre

Nome da participante:____

A Sra está sendo convidada a participar do estudo "Efeito da estimulação transcraniana por corrente contínua (ETCC) no período pré-operatório de tratamento cirúrgico de hálux valgo" que avalia o efeito do tratamento da dor por meio de estímulos de baixa intensidade aplicados na cabeça.

1. OBJETIVOS DESTE ESTUDO – A senhora será submetida, exceto em casos de recusa, à anestesia padrão (raquidiana) para correção cirúrgica de hálux valgo independentemente da participação neste estudo. Esse estudo tem como objetivo avaliar o efeito da aplicação de um método complementar a anestesia padrão para controle de dor pós-opetatória e ansiedade pré-operatória: a ETCC (que é um aparelho aplicado na cabeça que emite uma corrente elétrica).

2. EXPLICAÇÃO DOS PROCEDIMENTOS - Pretende-se avaliar o efeito da ETCC por meio de questionários, exames de sangue, líquor e testes específicos. A senhora terá que responder a algumas perguntas, preencher questionários e permitir uma coleta de sangue (5mL) ainda antes da aplicação da ETCC. Após o preenchimento dos questionários, serão feitos testes específicos para avaliar o quanto a Sra sente de dor ou desconforto ao aplicar um aparelho que esquenta gradativamente no seu braço. No dia seguinte, durante a anestesia raquidiana (que é aplicada independentemente da participação no estudo) será feita mais uma coleta de mínima quantidade de sangue (5mL) e líquor (1mL) para avaliar o nível de marcadores de dor contidas nessas amostras. O líquor é o líquido que está em contato com a medula espinhal e o cérebro e, em todas as anestesias raquidianas, o seu escoamento pela agulha inserida nas costas é utilizado para certificação de que a agulha está inserida no local ideal de aplicação do medicamento anestésico. Com a participação nesse estudo, será coletado 1mL desse líquido (quantidade rapidamente produzida e reposta pelo organismo do paciente, sem risco adicional aos inerentes da anestesia raquidiana). Após alta hospitalar, a Sra preencherá questionário padrão uma vez por dia a fim de registrar o quanto a senhora sente de dor. No dia do seu retorno ambulatorial (8º dia após cirurgia) a Sra trará os questionários preenchidos em casa e será feita a última coleta de sangue (5mL). A sua participação é voluntária. Se concordar, serão aplicados os questionários e daremos início ao estudo.

3. TRATAMENTOS - Neste estudo, as pacientes serão sorteadas em dois grupos. Um grupo irá receber um tratamento para alívio da dor, que consiste na aplicação na sua cabeça de dois eletrodos molhados através dos quais passará uma corrente elétrica fraca que pode causar uma leve coceira, formigamento e vermelhidão local em uma sessão que dura 20 minutos. O outro grupo de tratamento será o grupo placebo, o qual receberá um tratamento em que os equipamentos não emitirão estímulo. As sessões do tratamento serão realizadas conforme o protocolo deste estudo, no total de duas sessões (uma na noite anterior à cirurgia e uma na manhã do dia da cirurgia). A corrente é produzida por um aparelho (figura abaixo) produzido para este fim. Nem a Sra, nem o avaliador que lhe aplicará os questionários saberão qual tratamento a Sra recebeu.

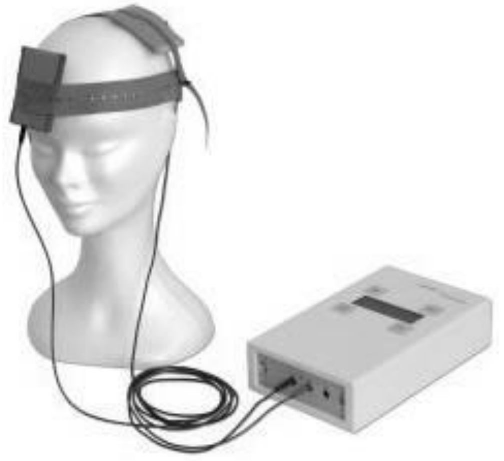

4. POSSÍVEIS RISCOS E DESCONFORTOS - Os possíveis desconfortos referentes à aplicação da ETCC são: pequenos choques, coceira e vermelhidão na região onde serão instalados os eletrodos da ETCC e, raramente, tonturas, dor de cabeça e vertigem. Algum desconforto poderá ser sentido também durante as coletas de sangue. Existe ainda, riscos inerentes à prática da anestesia raquidiana, que é aplicada mesmo em pacientes não participantes da pesquisa: dor de cabeça pós-punção lombar, desconforto em sítio de punção lombar, queda da pressão, infecção, sequela neurológica transitória ou permanente.

5. POSSÍVEIS BENEFÍCIOS DESTE ESTUDO – A participação no estudo tem como benefício à participante um possível melhor controle da dor e ansiedade, e ainda, contribuirá para o aumento do conhecimento sobre o assunto estudado. Os resultados desse estudo poderão trazer informações importantes sobre o papel da estimulação transcraniana por corrente direta no controle da dor pós-operatória. Poderemos iniciar o uso alternativo de uma técnica que poderá beneficiar outros pacientes com quadros de dor semelhantes ao seu.

6. RESSARCIMENTO DE DESPESAS - A Sra não terá despesas com a sua participação na pesquisa e realização dos procedimentos envolvidos. Também não está previsto nenhum tipo de pagamento pela participação no estudo.

7. DIREITO DE DESISTÊNCIA - A senhora pode desistir de participar a qualquer momento. Suas decisões de não participar ou de deixar a pesquisa depois de iniciada, não afetará a continuação de qualquer atendimento dentro da intituição.

8. EXCLUSÃO DO ESTUDO - O investigador responsável poderá excluí-la do estudo, sem o seu consentimento, quando julgar necessário, para o melhor encaminhamento do seu caso ou se a senhora não cumprir o programa estabelecido.

9. PRIVACIDADE - Todas as informações obtidas deste estudo poderão ser publicadas com finalidade científica, preservando os dados de identificação.

10. CONTATO DOS PESQUISADORES - Caso a Sra tenha alguma dúvida, poderá entrar em contato com os pesquisadores através dos telefones: Dr. Wolnei Caumo (2º andar do HCPA Laboratório de Dor & Neuromodulação - sala 2201E – telefone 3359-8083) ou ainda com o Comitê de Ética e Pesquisa do Hospital de Clínicas - telefone 3359-8304 – das 8h às 17h.

11. CONSENTIMENTO – Este termo de Consentimento Livre e Esclarecido será fornecido uma via para a Sra e uma via será arquivada pelo pesquisador, sendo as duas vias assinadas e rubricadas todas as páginas por ambos. Declaro ter lido - ou me foram lidas - as informações acima antes de assinar este formulário. Foi-me dada ampla oportunidade de fazer perguntas, esclarecendo plenamente minhas dúvidas. Por este instrumento, tomo parte, voluntariamente, do presente estudo.

Nome da participante ou representante legal: ____

Assinatura da participante ou representante legal: ____

Grau de parentesco do representante: ____

Nome do pesquisador: ____

Assinatura do pesquisador: ____

Porto Alegre, ______de ________________de 20____.
